# Glycoside hydrolase from the GH76 family indicates that marine Salegentibacter sp. Hel_I_6 consumes alpha-mannan from fungi

**DOI:** 10.1038/s41396-022-01223-w

**Published:** 2022-04-12

**Authors:** Vipul Solanki, Karen Krüger, Conor J. Crawford, Alonso Pardo-Vargas, José Danglad-Flores, Kim Le Mai Hoang, Leeann Klassen, D. Wade Abbott, Peter H. Seeberger, Rudolf I. Amann, Hanno Teeling, Jan-Hendrik Hehemann

**Affiliations:** 1 Max Planck Institute for Marine Microbiology, Celsiusstraße 1, 28359 Bremen, Germany; 2 Max Planck Institute of Colloids and Interfaces, Am Mühlenberg 1, 14476 Potsdam, Germany; 3 Lethbridge Research and Development Centre, Agriculture and Agri-Food Canada, 5403 1st Avenue South, Lethbridge, AB T1J 4B1 Canada; 4 University of Bremen, Center for Marine Environmental Sciences, MARUM, Leobener Straße 8, 28359 Bremen, Germany; 5 Present Address: GlycoUniverse GmbH & Co KGaA, Am Mühlenberg 11, 14476 Potsdam, Germany

**Keywords:** Metagenomics, Microbial ecology, Structural biology, Fungal ecology, Molecular ecology

## Abstract

Microbial glycan degradation is essential to global carbon cycling. The marine bacterium *Salegentibacter* sp. Hel_I_6 (*Bacteroidota*) isolated from seawater off Helgoland island (North Sea) contains an α-mannan inducible gene cluster with a GH76 family endo-α-1,6-mannanase (ShGH76). This cluster is related to genetic loci employed by human gut bacteria to digest fungal α-mannan. Metagenomes from the Hel_I_6 isolation site revealed increasing GH76 gene frequencies in free-living bacteria during microalgae blooms, suggesting degradation of α-1,6-mannans from fungi. Recombinant ShGH76 protein activity assays with yeast α-mannan and synthetic oligomannans showed endo-α-1,6-mannanase activity. Resolved structures of apo-ShGH76 (2.0 Å) and of mutants co-crystalized with fungal mannan-mimicking α-1,6-mannotetrose (1.90 Å) and α-1,6-mannotriose (1.47 Å) retained the canonical (α/α)_6_ fold, despite low identities with sequences of known GH76 structures (GH76s from gut bacteria: <27%). The apo-form active site differed from those known from gut bacteria, and co-crystallizations revealed a kinked oligomannan conformation. Co-crystallizations also revealed precise molecular-scale interactions of ShGH76 with fungal mannan-mimicking oligomannans, indicating adaptation to this particular type of substrate. Our data hence suggest presence of yet unknown fungal α-1,6-mannans in marine ecosystems, in particular during microalgal blooms.

## Introduction

Photosynthetic primary production fixes about 105 Gt of carbon annually and is carried out in about equal amounts by terrestrial plants and marine algae [1], with uni- to pluricellular planktonic microalgae accounting for about 40% of the marine primary production [2]. Primary production by planktonic microalgae is less constant than by macroalgae and can culminate during massive algae blooms. Such blooms are transient and highly dynamic events, with often multiple alga species blooming in short succession. During such blooms algae produce substantial quantities of organic molecules that in turn trigger blooms of heterotrophic bacteria and various zooplankton. Many factors contribute to algae bloom termination, such as nutrient depletion, predation (e.g., by copepods), and viral and parasitic infections. The latter include parasitic flagellates [3], oomycetes [4] and marine fungi [5]. However, the role that such parasites, and in particular marine fungi, play in controlling algae blooms and thus marine primary production is not well understood and possibly underestimated [6].

A substantial fraction of the biomass that is turned over and re-mineralized during algae blooms consists of polysaccharides. Polysaccharides are major constituents of phytoplankton and macroalgae [7] as well as of dissolved (DOM) and particulate organic matter (POM) [8]. To investigate the breakdown of marine polysaccharides remains challenging due to their often complex structures, which result from variations in monomer composition, monomer linkage types, polymer lengths, branching patterns and the addition of functional groups such as e.g., sulfate, methyl, methoxy, or acetyl groups [9]. The direct measurement of marine polysaccharides in situ by spectrophotometric methods is hampered by methodological challenges that arise from molecular properties such as solubility, charge, and size [10, 11]. More indirect measurements using monoclonal antibodies is also challenging [12, 13] and require isolation of pure polysaccharides as suitable epitopes for antibody production. Microarrayed antibodies were for example recently used to elucidate the composition of sinking POM, also known as marine snow [14]. An alternative indirect yet more successful approach is to study the genetic and enzymatic machineries that mediate the decomposition of marine polysaccharides in marine heterotrophic bacteria. The strategy of applying polysaccharide-degrading enzymes from marine bacteria to quantify marine polysaccharides, such as for example algal laminarin, has proven to be more efficient than chemical hydrolysis followed by monomer analysis [15].

Metagenome studies of bacterioplankton at times of spring algae blooms in the southern North Sea have shown high abundances of carbohydrate-active enzymes (CAZymes) for polysaccharide degradation [16, 17]. The genomes of *Bacteroidota* in particular that have been isolated from this site are often enriched in genetic clusters of putative CAZymes that target specific polysaccharides [18–21]. These gene clusters have been termed polysaccharide utilization loci (PULs) [22, 23]. Sequence information from PULs have enabled heterologous cloning and recombinant expression of crucial CAZymes, resulting in detailed biochemical and functional characterizations. So far, this approach has been particular successful for human gut bacteria. Examples are the discovery of CAZyme lateral gene transfer between marine and human gut bacteria [24], and the elucidation of the degradation pathways for plant xyloglucan [25, 26], yeast α-mannan [27], and plant pectins, such as rhamnogalacturonan II [28]. A recent example for marine bacteria is a study on the degradation of the sulfated green algal polysaccharide ulvan [29].

The marine bacteroidete, *Salegentibacter* sp. Hel_1_6, was sampled from seawater in the southern North Sea off Helgoland island [30] and its genome features PULs that are inducible by α- and β-mannan, respectively [31]. Mannan degradation has been extensively studied in the human gut bacterium *Bacteroides thetaiotaomicron* VPI-5482^T^ for yeast (*Saccharomyces cerevisiae*) α-mannan [27], and in *Bacteroides* ATCC 8483 for carob (*Ceratonia siliqua*) β-mannan [32], but rarely in any marine bacterium, even though various types of mannans are known from marine organisms. For instance*, Flavimarina* sp. Hel_I_48 and *Arenibacter palladensis* MAR_2009_79 both features PULs that target mannan or mannose-rich substrates [31]. In a recent study a sulfated glucuronomannan was purified from the marine diatom *Phaeodactylum tricornutum* and was shown to have a linear α-1,3-mannan backbone [33]. Likewise, a sulfated α-1,3-linked D-mannan has been described in the red seaweed *Nothogenia fastigiate* [34], and mixed-linkage α-1,3-/β-d mannans were discovered in the marine green alga *Codium fragile* [35]. Marine fungi are also known to produce diverse mannans, including α-1,6-linked mannans [36, 37].

The mannan monomer mannose in general plays an important role in the marine carbon cycle. Bioinformatic PUL analyses of North Sea *Bacteroidota* genomes have shown that about a third contain CAZymes suggesting degradation of mannose-rich substrates, in particular GH92 family exo-α-mannosidases [19]. The latter are also known to partake in the degradation of genuine α-mannans [27], and have been found to be expressed in a previous metaproteome study of bacterioplankton during a North Sea spring algae bloom [38] (supplement). GH92 family genes were also frequently identified in bacterioplankton metagenomes during four consecutive years of North Sea spring blooms [17], as well as in the open North Atlantic [39]. These observations indicate substantial levels of substrate-driven selection of marine bacteria by mannose-rich algal polysaccharides. However, the role of genuine α-mannans in this selection process is as yet unclear. The α-mannan-inducible PUL of *Salegentibacter* sp. Hel_I_6 encodes multiple GH92 family exo-mannosidase, but its key enzyme is a sole GH76 family endo-α-1,6-mannanase (henceforth ShGH76). This suggests that the PUL’s target substrate has an α-1,6-linked backbone.

In this study we investigate ShGH76 from the α-1,6-mannan inducible PUL of *Salegentibacter* sp. Hel_I_6. This PUL is of particular interest as it is partially syntenic to an α-1,6-mannan-specific PUL in the well-studied human gut bacteroidete *B. thetaiotaomicron* VPI-5482^T^, yet most of the encoded enzymes are only distantly related to functionally characterized homologs or have completely unknown functions [31]. We cloned, expressed, and purified ShGH76, demonstrated its endo-α-1,6-mannanase activity, and subsequently solved the crystal structure of the ShGH76 apo-form at 2.0 Å resolution and of inactive mutants with bound α-1,6-mannotetrose (Man4) and α-1,6-mannotriose (Man3) at 1.90 Å and 1.47 Å resolution, respectively. We discuss differences with previously solved GH76 structures, and put our results into an environmental context based on (i) comparative analyses of GH76-containing PULs of yet known *Salegentibacter* species, (ii) metagenome time series of bloom-associated bacteria from the Hel_I_6 sampling site, and (iii) analyses of metagenomic GH76 sequence diversity. Our results support the view that diverse and readily degradable α-1,6-mannans are produced during algae blooms that are distinct from α-1,6-mannans targeted by human gut *Bacteroidota*. Such α-1,6-mannans are unknown to marine algae, but known to occur in marine fungi.

## Materials and methods

### Sequence data

*Salegentibacter* sp. Hel_I_6 was isolated during spring from a surface seawater sample at the island Helgoland (54° 11’ 03” N, 7° 54’ 00” E) in the southern North Sea [30]. Its genome was sequenced at the Department of Energy Joint Genome Institute (DOE-JGI, Berkeley, CA, USA) in the framework of the Community Sequencing Project No. 998 COGITO as described previously [19]. Metagenome sequences used in this study were obtained from surface seawater at the same sampling site during the years 2010, 2011 and 2012 [16, 17], 2016 (ENA project PRJEB28156), and 2018 (ENA project PRJEB38290) using fractional filtration (0.2–3 µm, 3–10 µm, >10 µm). Three additional sediment metagenomes were included that were obtained at Helgoland in 2016 (NCBI SRA: SRX9088755, SRX9088749, SRX9088778). An overview is provided in Supplementary Table S1.

### Metagenome analysis

Quality filtering and read trimming were performed as described previously [16]. Metagenomes from 0.2 to 3 µm filter fractions and sediments were assembled using metaSPAdes v3.10-v3.12 [40]. Metagenomes from 3–10 µm and >10 µm filter fractions were assembled using MEGAHIT v1.2.9 [41]. Details are provided in Supplementary Table S1. Contigs below 2.5 kbp (0.2–3 µm), 1.5 kbp (3–10 µm, >10 µm) and 1 kbp (sediment) were excluded from further analyses.

Prodigal v2.6.3 [42] was used for gene prediction (–p meta option). Polysaccharide degradation genes were annotated as described previously [16] with dbCAN v8 [43] and CAZy [44] as of 2019-07-31. Sequences of predicted GH76 and GH92 genes were both clustered at 99% identity using CD-HIT v.4.8.1 [45]. Reads from all metagenomes were mapped to all cluster representatives, and the resulting SAM files were filtered as described previously [16]. Relative GH76 and GH92 gene abundances were calculated as reads per kilobase per million [RPKM = (number_of_mapped_reads_on_gene * 1 000 000) / (length_of_gene_in_kbp * sum_of_reads)].

### Salegentibacter PUL comparisons

All available 24 sequenced *Salegentibacter* strains as of April 2021 on NCBI website were searched for GH76-containing putative α-1,6-mannan-targeting PULs (Supplementary Table S2). Five species (six strains) had such PULs, which were aligned using clinker v.0.0.20 [46].

### Phylogenetic tree construction

Eighty three GH76 family protein sequences (64 from metagenome data, eleven from the CAZy database, and eight from five GH76-containing *Salegentibacter* species) (Supplementary Table S3) were aligned using MUSCLE (default parameters) in MEGA X v. 10.1.18 [47, 48], resulting in an alignment of 1 206 positions. Seed trees for Maximum Likelihood searches were obtained by applying Neighbor-Join and BioNJ algorithms to a matrix of pairwise distances estimated by the JTT model, and then selecting those with superior log likelihood values. Phylogeny was finally inferred using Maximum Likelihood with the JTT matrix-based model. All analyses were conducted in MEGA X and trees were visualized using iTOL [49].

### Construct design and site-directed mutagenesis

An N-terminal 6x-histidine (NTH) tagged construct of ShGH76 was designed for Ni-NTA (nitrilotriacetic acid) based immobilized metal-ion affinity chromatography purification (IMAC, see below). The synthetic gene was cloned into the pET28a(+) vector using the NheI and XhoI restriction sites (GenScript pvt Ltd., Piscataway, NJ, USA). The WT plasmid was used as template to engineer ShGH76 mutants by site-directed mutagenesis [50]. Six mutants were constructed (D136A, D137A, D136A-D137A, D136E, D137E and D136E-D137E). Similarly, NTH tagged constructs of six GH92s were cloned into the pET28a(+) vector using combinations of the NdeI/NheI and XhoI/NotI restriction sites. The primers used to perform mutagenesis and GH92 cloning are listed in Supplementary Table S4. All clones in this study were verified by DNA sequencing.

### Heterologous protein expression and purification by IMAC and size exclusion chromatography

*Escherichia coli* BL21DE3 cells harboring pET28a-GH76 were cultured in 1 L lysogeny broth (LB) medium supplemented with 50 µg/mL kanamycin at 37 °C until the mid-exponential phase (OD_600nm_ 0.6–0.8). Recombinant gene expression was induced by addition of 0.3 mM (final concentration) isopropyl β-D-1-thiogalactopyranoside (IPTG) and further incubation at 16 °C for ~16 h.

In case of the ShGH76 clones, cells were harvested by centrifugation and stored at −20 °C. Cell lysis was conducted chemically as described previously [51]. Frozen cell pellets were resuspended in 20 mL sucrose solution (25% w/v sucrose, 50 mM Tris HCl, pH 8.0). Lysozyme was added at a concentration of 1 mg/mL and the cells were subsequently incubated for 10 min. at room temperature with stirring 40 mL of deoxycholate solution (1% w/v deoxycholate, 1% w/v Triton X-100, and 100 mM NaCl) was added followed by MgCl_2_ to a final concentration of 1 mM and DNase to a concentration of 1 mg/mL. The resulting lysate was centrifuged at 16,000 × *g* for 45 min at 4 °C. For purification, clarified lysate was applied to a 5 mL prepacked IMAC column (GE Healthcare Life Sciences, Marlborough, MA, USA) equilibrated in buffer A (20 mM Tris-HCl, pH 8 and 500 mM NaCl) using an ÄKTA start FPLC (fast protein liquid chromatography) system (Cytiva, Marlborough, MA, USA). The column was washed with buffer A and the His-tagged protein was eluted using a gradient of imidazole to 500 mM in Buffer A. The purified protein was concentrated using a stirred cell ultrafiltration device with a 10 kDa membrane and subsequently further purified using size exclusion chromatography [using HiPrep Sephacryl S200 HR column (Cytiva, Marlborough, MA, USA)] in 20 mM Tris-HCl, pH 8 with 250 mM NaCl. Finally, the protein was concentrated to 20 mg/mL prior to further experiments as determined by absorbance at 280 nm using the extinction coefficient of 2.083 for ShGH76 [52].

In case of the GH92 clones, cells were harvested by centrifugation and resuspended in 20 mM Tris-HCl, pH 8 with 250 mM NaCl buffer. The cells were lysed by sonication (Bandelin sonopuls, Berlin, Germany) for 10 min (alternating 10 s of 60% amplitude with 20 s break). The resulting lysate was centrifuged (16,000 × *g*, 45 min, 4 °C). Further purification was performed as mentioned above. The elution fractions were concentrated and buffer-exchanged with 20 mM Tris-HCl (pH 8) with 250 mM NaCl buffer using an ultrafiltration device with a 50 kDa membrane (4 °C, 3500 g). All six purified GH92 proteins were used for activity assays.

### Chemical synthesis of linear α-1,6-mannooligosaccharides

Linear α-1,6-mannooligosaccharides ranging from mannobiose (Man2) to mannoheptose (Man7) were synthesized and provided by the Peter H. Seeberger’s research group at the Max Planck Institute of Colloids and Interfaces (Potsdam, Germany). Building blocks for the synthesis were purchased from GlycoUniverse (Potsdam, Germany). Automated syntheses were performed on a home-built synthesizer developed at the Max Planck Institute of Colloids and Interfaces (Golm, Germany) [53]. Further details on chemical synthesis are provided in the Supplementary Text.

### Crystallization, data collection, structure solution and refinement

An ShGH76^WT^ solution of 52 mg/mL was used for initial crystallization attempts. Crystallization was performed in three drop 96-well crystallization plates in sitting drop format using commercial crystallization screens. Plates were incubated at 16 °C. Start of crystal formation was observed within 2–3 days. Diffraction quality crystals were obtained using 0.2 M magnesium chloride hexahydrate, 0.1 M sodium acetate pH 5.0 and 20% PEG 6000. Diffraction data was collected on EMBL beamline P14. A total of 3600 images were obtained (exposure: 0.1 s; oscillation: 0.1°). Measured reflection intensities were indexed, integrated and scaled using XDS [54, 55]. The ShGH76^WT^ structure was solved to 2.0 Å using molecular replacement with the *Listeria innocua* protein Lin0763 (PDB ID: 3K7X) as reference in PHASER [56]. For automatic model building, the ARP/wARP server was used [57]. Refinement of the initial model was done in PHENIX.REFINE [58], and further iterative rounds of manual model building were carried out using COOT [59].

For mutants, 5 mg/mL proteins were used together with 0.5 mg/mL of Man2-Man7 oligosaccharides in three well crystallization plates using commercial crystallization screens. First crystals of the double D136A and D137A mutant (ShGH76^Ala^) appeared after 2 months in 1.6 M tri-sodium citrate, whereas crystals of the double D136N and D137N mutant (ShGH76^Asn^) appeared after 5 months in 14% v/v 2-propanol, 30% v/v glycerol, 70 mM sodium acetate pH 4.6 and 140 mM calcium chloride. Crystals were only obtained with Man3 and Man4 oligosaccharides. Diffraction data was collected on beamline P11 at the German Electron Synchrotron DESY (Hamburg, Germany). A total of 3600 images were obtained (exposure: 0.1 s; oscillation: 0.1°). Data were processed using iMOSFLM [60] and scaled using SCALA. The structures of ShGH76^Ala^ and ShGH76^Asn^ were solved at 1.90 Å and 1.47 Å resolution, respectively, using molecular replacement with the ShGH76^WT^ as reference in PHASER. For automatic model building, PHENIX.AUTOBUILD was used [61]. The initial model was refined using PHENIX.REFINE and further iterative rounds of the manual model building were carried out using COOT. The ligands Man4 and Man3 were fitted using COOT, and ligand refinement was performed in REFMAC5 [62, 63].

Both models and structure factors for ShGH76^WT^ and the ShGH76^Ala^ and ShGH76^Asn^ mutants were deposited in the Protein Data Bank (PDB) with accessions 6SHD, 6SHM and 6Y8F, respectively. Corresponding data-processing and refinement statistics are summarized in Supplementary Table S5. The structural comparison of ShGH76 and the six known family members were performed using the PyMOL v.2.3.2 (Schrödinger, New York, NY, USA).

### Determination of enzyme activity by high-performance anion-exchange chromatography- pulsed amperometric detection (HPAEC-PAD)

Purified N-terminal 6x-histidine tagged ShGH76^WT^ and ShGH76^Ala^ proteins were used in HPAEC-PAD experiments together with synthetic α-1,6-mannooligosaccharides ranging from Man2 to Man7 (Supplementary Fig. S1). A detailed protocol is provided in the Supplementary Text.

### Substrate-binding analysis by acrylamide gel electrophoresis (AGE)

Acrylamide gels (12%) were prepared containing 1% w/v polysaccharide and without polysaccharide as control. We tested AGE with yeast α-mannan. Gels were loaded with either 1.25 µg of ShGH76^WT^, ShGH76^Ala^ mutant, bovine serum albumin (BSA), and α-mannan-specific SusD-like protein from *Salegentibacter* sp. Hel_I_6. Heterologously expressed SusD protein has been shown to bind to *S. cerevisiae* α-mannan in a previous study [64]. Migration velocities were quantified between the control and polysaccharide containing gels. AGE was performed for 2 h at a voltage of 80 V on ice. Proteins were visualized by staining with 0.1% w/v Coomassie brilliant Blue R-250.

### ShGH76 mutant dot-blots

In order to assess binding of ShGH76 mutants not only to synthetic α-1,6-mannooligosaccharides, but also to natural α-1,6-linked mannans, we used dot-blots with natural and modified (linear or debranched) yeast α-mannan [27, 65]. A detailed protocol is provided in the Supplementary Text.

### Fluorophore-assisted carbohydrate electrophoresis (FACE) of digestion products

ShGH76 enzyme substrate reactions were set up with a final concentration of 10 µg/mL ShGH76^WT^ and 0.5 mg/mL polysaccharide substrate (linear yeast α-mannan and yeast α-mannan dissolved in MilliQ water). For the six GH92 proteins, 100 µL reactions were prepared with final concentrations of 50 µg/mL protein and 6 mg/mL yeast α-mannan. Reactions were incubated at 37 °C on a heating block, and aliquots were taken after 1, 2, 5, 10, 20, 30 min and 16 h. These aliquots were analyzed by FACE as follows: 200 µL of each aliquot were dried in a speed-vacuum centrifuge and the oligosaccharides were labeled with 8-aminonaphthalene-1,3,6-trisulfonate (ANTS) in a protocol adapted from Starr and Masada [66]. For derivatization with ANTS, the pellet was redissolved in 2 µL of ANTS solution (0.15 M ANTS in acetic acid/water (3:17, v/v)). Then 5 µL of 1 M sodium cyanoborohydride in dimethyl sulfoxide (DMSO) was added, and the mixture was incubated for 16 h at 37 °C. The samples were analyzed on a 29% polyacrylamide running gel with a 10% stacking gel and a BioRad gel system (BioRad, Hercules, CA, USA). Gels were run for half an hour at 100 V followed by 300 V for 1 h at 4 °C or on ice.

## Results

### ShGH76 homologs occur in 25% of all sequenced Salegentibacter strains

As yet 24 *Salegentibacter* strains (taken from GenBank NCBI) have been sequenced, originating from diverse habitats such as temperate and arctic marine seawater, marine sediments, solar salterns, wastewater plants, and diverse marine vertebrates (sea urchins, holothurians and sponges). Six of these strains (25%) representing five species feature GH76 genes (Supplementary Table S2) [67–73].

**Fig. 1:**
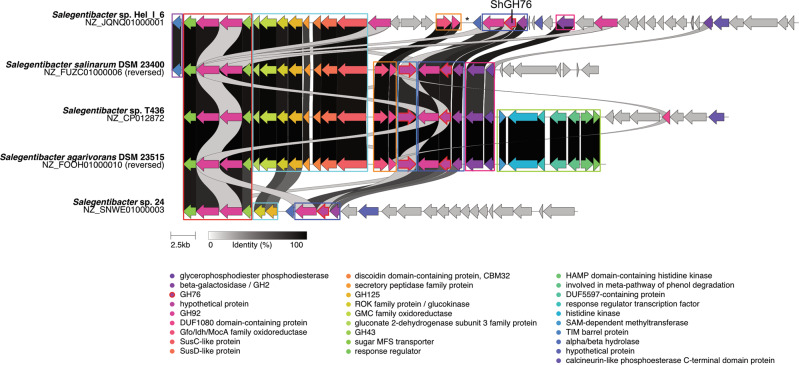
Gene synteny of GH76-containing PULs in *Salegentibacter* strains. The asterisk marks remnants of a former transposase gene.

*Salegentibacter* GH76 genes reside in PUL-like structures that feature both a high proportion of syntenic gene modules as well as considerable variations (Fig. 1). Genetic variations likely represent adaptations toward targeted substrates, possibly by modifications within small modules of functionally related genes. Some changes might have resulted in a loss of function, as for example in *S*. sp. strain 24, where the PUL is rather short and even lacks the SusC/D pair. Three of the five GH76-containing *Salegentibacter* species contain two GH76 genes, strain Hel_I_6 contains only one of the two. In place of the second GH76 gene are remnants of a transposase gene, which are known to be involved in gene loss [74]. The most similar PUL to that of strain Hel_I_6 is present in *S. salinarum* DSM 23400. The latter also contains two GH76 genes, one of which contains an additional esterase domain, indicating a functional speciation. This is supported by a comparison of the ShGH76^WT^ structure with Phyre2 [75] modeled structures of the two *S. salinarum* homologs, with one homolog being substantially more similar to ShGH76^WT^ than the other (sequence identities: 91% for SsGH76 SKB63511 and 55% for SsGH76 SKB63521). The overall folds of ShGH76^WT^ and SKB63511 are similar, but the active site of the latter does not seem capable of housing Man4 due to steric clashes observed of the modeled substrate with active site residues (Supplementary Fig. S2).

GH76 speciation, PUL compositional variations and distinct habitats indicate that GH76-containing *Salegentibacter* strains do not target identical α-mannan substrates. There is no evident association between habitat and presence of GH76-containing PULs. For example, strains *S. salarius* DSM 23401 and *S. salinarum* DSM 23 400 both stem from solar salterns, but only the latter features a ShGH76 homolog. The only consensus is that all sediment strains known so far lack ShGH76 homologs (Supplementary Table S2).

### GH76 family genes occur in free-living bacteria found in marine algae blooms

**Fig. 2:**
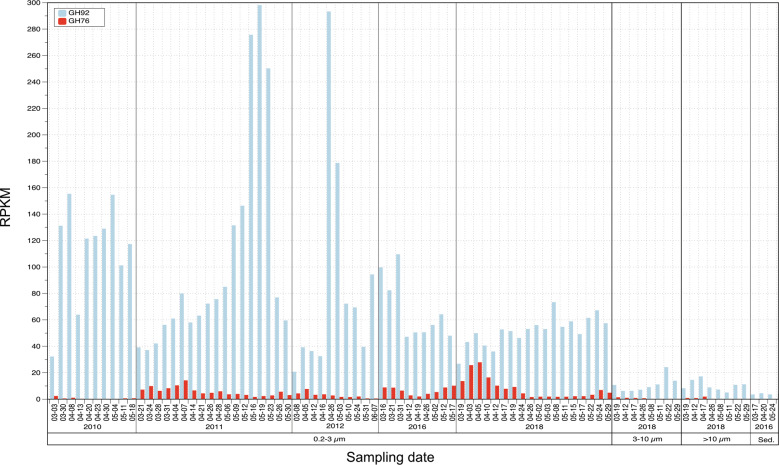
Frequencies of GH76 and GH92 genes in metagenomes sampled from the *Salegentibacter* sp. Hel_I_6 isolation site during spring phytoplankton blooms in the years 2010, 2011, 2012, 2016 and 2018. Samples were taken for free-living bacteria (0.2–3 µm), two size fractions of particle-attached bacteria (3–10 µm; >10 µm) and from sediments (Sed.). Frequencies are expressed as reads per kilobase million (RPKM).

We analyzed gene frequencies of the putative GH76 family endo-α-1,6-mannanase in metagenomes that we obtained from the *Salegentibacter* sp. Hel_I_6 isolation site during spring phytoplankton blooms in 2010–2012, 2016 and 2018. In order to gauge if the observed changes were meaningful, we also analyzed GH92 gene frequencies as a point of reference. GH92 exo-α-mannosidases are involved in the degradation of mannans and other mannose-rich substrates. Metagenomes were obtained from different water fractions (0.2–3 µm, 3–10 µm, >10 µm) and sediment, allowing to compare free-living (0.2–3 µm), particle-attached bacteria (3–10 µm and >10 µm) and sediment bacteria. While GH76 frequencies were substantially lower than GH92 frequencies in all metagenomes (Fig. 2), they increased in the free-living fractions during blooms in March to April in 2011, 2012, 2016 and most notably 2018, where they peaked at more than half of the GH92 frequencies. This increase in GH76 genes indicates that target α-1,6-mannans were present during these blooms. In contrast, the particle-attached fractions featured only few GH76 genes, and the sediments were almost devoid of such genes.

### GH76 sequences are diverse

**Fig. 3:**
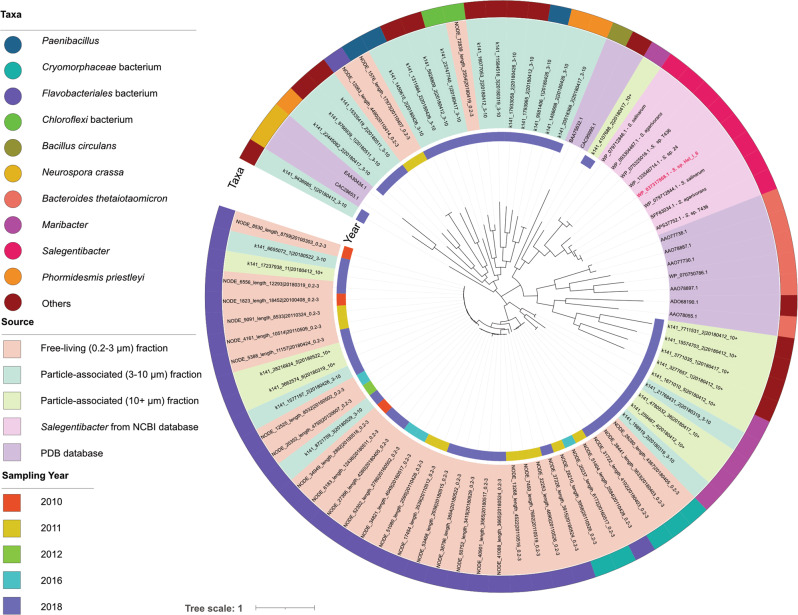
Phylogenetic tree of GH76 protein sequences from different genomes and metagenomes: 8 from *Salegentibacter* species, 11 from the CAZyme database, 64 from the Hel_I_6 sampling site near the North Sea island Helgoland. The tree was inferred by using the Maximum Likelihood method with the JTT matrix-based model. The tree with the highest log likelihood (-42104.84) is shown. The percentage of trees in which the associated taxa clustered together is shown next to the branches. There were a total of 1,206 positions in the final dataset. Evolutionary analyses were conducted in MEGA X. The bootstrap value is 500.

Phylogenetic analysis of 83 bacterial GH76 protein sequences from metagenomes and genomes revealed pronounced clustering (Fig. 3). *Salegentibacter* sequences from different habitats clustered (without habitat-specific sub-clustering), as did sequences from the different planktonic filter fractions. Most similar to *Salegentibacter* GH76s was a *Maribacter* sequence from the >10µm fraction (57% sequence similarity), whereas no sequence from the 0.2–3 µm fraction clustered with the *Salegentibacter* sequences (<30% sequence similarity). Similarity of GH76 to homologs from human gut bacteria was below 27%.

### The Salegentibacter sp. Hel_I_6 α-mannan PUL consists of many genes

**Fig. 4:**
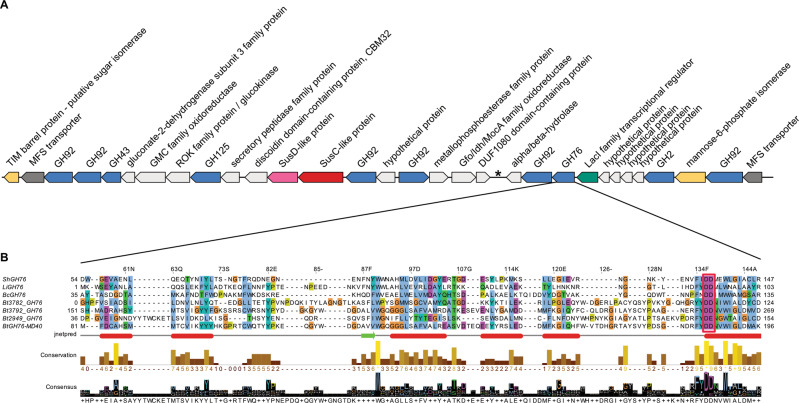
ShGH76 is the only key endo-acting enzyme in the Hel_I_6 α-mannan PUL. **A** α-Mannan PUL of *Salegentibacter* sp. Hel_I_6. The asterisk marks remnants of a transposase gene. **B** 3D structure-guided multiple sequence alignment of the *Salegentibacter* sp. Hel_I_6 GH76 (ShGH76) with homologs. The conserved two catalytic aspartate residues (DD) are highlighted by a red box. LiGH76: *Listeria innocua* GH76; BcGH76: *Bacillus circulans* GH76; BTGH76-MD40, BT3782_GH76, BT3792_GH76 and BT2949_GH76: *Bacteroides thetaiotaomicron* GH76s.

The α-mannan inducible PUL of *Salegentibacter* sp. Hel_I_6 comprises about 30 genes including glycoside hydrolase genes of families GH92 (6x), GH43 (1x), GH125 (1x), GH2 (1x) and GH76 (1x) (Fig. 4A). The GH43 and GH2 genes are most closely related to the α-l-arabinofuranosidases of *B. thetaiotaomicron* VPI-5482^T^ (BT3675, GH43) [28] and *Thermotoga thermarum* DSM 5069 (TtAFase, GH2) [76]. Known members of the GH92 family are exo-acting α-mannosidases [77], and members of the GH125 family are also exo-acting α-mannosidases with a specificity for α-1,6-linked non-reducing terminal mannose residues [78]. *B. thetaiotaomicron* VPI-5482^T^ codes for four α-mannan-related GH92 family proteins (BT3784, BT2629, BT3858, and BT3773) that share 63%, 62%, 31%, and 41% sequence similarity with the most similar of the six GH92 family proteins of strain Hel_I_6 (Supplementary Table S6). Likewise, *B. thetaiotaomicron* VPI-5482^T^ codes for two GH125 family proteins (BT2632 and BT3781) that share 62% and 58% sequence similarity with the sole GH125 family protein of strain Hel_I_6. ShGH76 is the only endo-acting enzyme in the Hel_I_6 α-mannan PUL and thus the only enzyme that can cleave the backbone of larger α-1,6-linked mannans into smaller α-mannan oligosaccharides.

### ShGH76 has endo-α-1,6-mannanase activity

**Fig. 5:**
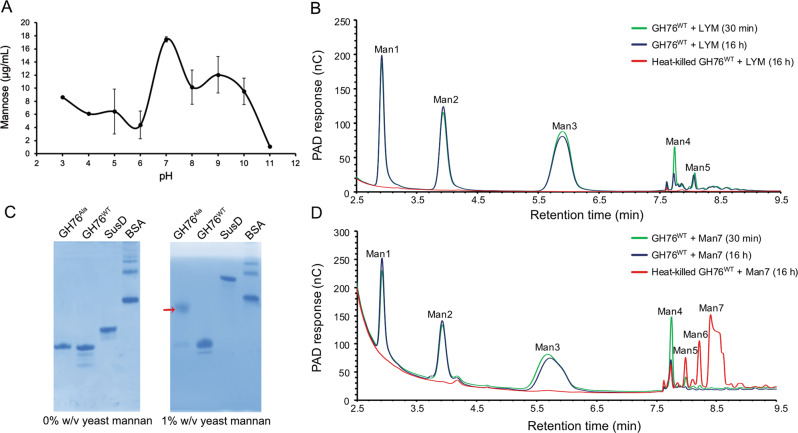
ShGH76 exhibits endo-mannanase activity on linear yeast α-mannan and linear mannooligosaccharides. **A** Activity of ShGH76^WT^ on yeast α-mannan from pH 3.0-11.0 using a PAHBAH assay; error bars represent S.D. over at least three replicates. **B** HPAEC-PAD chromatograms of linear yeast α-mannan (LYM) incubated with ShGH76^WT^ for 30 min and after 16 h. The heat-killed ShGH76^WT^ is control. The peaks were labeled with corresponding products formed. **C** Affinity gel electrophoresis suggesting yeast α-mannan binding by the ShGH76^Ala^ mutant (arrow). **D** HPAEC-PAD chromatograms of mannoheptose (Man7) in the presence of the ShGH76^WT^. Peaks are labeled with corresponding products. Man1: mannose, Man2: α-1,6-linked-mannobiose, Man3: α-1,6-linked-mannotriose, Man4: α-1,6-linked-mannotetrose, Man5: α-1,6-linked-mannopentose, Man6: α-1,6-linked-mannohexose, and Man7: α-1,6-linked-mannoheptose.

Purified ShGH76^WT^ exhibited endo-α-1,6-mannanase activity in HPAEC-PAD analyses, with an optimum at pH 7.0 (Fig. 5A). ShGH76^WT^ digested linear α-1,6-mannan, i.e., mannan consisting only of an α-1,6-linked mannan backbone (obtained from a mutant strain of yeast [65]). Products comprised mannose (Man1), mannobiose (Man2), Mannotriose (Man3), mannotetrose (Man4), and mannopentaose (Man5) (Fig. 5B), as was confirmed by HPLC with synthetic oligosaccharide references (Supplementary Fig. S1). We also analyzed hydrolysis of synthetic Man2 to Man7 α-1,6-mannooligosaccharides (Supplementary Fig. S9). As is exemplarily shown for Man7 (Fig. 5D), ShGH76^WT^ hydrolyzed α-1,6-mannooligosaccharides almost completely into shorter oligomers down to monomeric mannose. In contrast, ShGH76^WT^ digested branched yeast α-mannan only slowly or even incompletely into Man1, Man2, and Man3 (Supplementary Fig. S8A). In corresponding FACE, only faint bands of digestion products were obtained after 30 min and even after 16 h (Supplementary Fig. S3A, right). Digestion of linear yeast α-mannan was much faster as FACE showed a characteristic ladder-like pattern of digestion products already in the initial phase of the enzyme-substrate reaction (Supplementary Fig. S3A, left).

Preference of ShGH76 for linear α-1,6-mannan was confirmed in kinetic experiments (Supplementary Fig. S8B). The *K*_*M*_ value (0.36 ± 0.07 mg/mL) suggests that ShGH76 has a higher affinity towards linear yeast α-1,6-mannan than human gut bacterial GH76 homologs (BT2623: 1.9 ± 0.4 mg/mL; BT3782: 0.43 ± 0.2 mg/mL; BT3792: 2.4 ± 1.1 mg/mL; supplementary data of [27]). These results suggest that ShGH76 requires removal of the branches before it can effectively digest unmodified, branched yeast α-mannan. GH92 exo-mannosidases are known to cleave off side-chains during yeast α-mannan degradation in *B. thetaiotaomicron* [27]. We tested all six ShGH92s for exo-mannosidase activity on yeast α-mannan. This could be confirmed for three of these, where the FACE gel showed release of mannose monomers (Supplementary Fig. S8C).

On overall, ShGH76 exhibited endo-α-1,6-mannanase activity with a preference for linear α-1,6-mannans. Three of the ShGH92 proteins furthermore showed exo-mannanase activities, corroborating the yeast α-mannan degrading potential of the target PUL in *Salegentibacter* sp. Hel_I_6.

### ShGH76 has two aspartates as core catalytic residues

Two adjacent aspartate residues are usually involved in substrate hydrolysis through a retaining mechanism as reported for the BT3792 GH76 of *B. thetaiotaomicron* VPI-5482^T^ [27]. Similarly, BcGH76 from *Bacillus circulans* TN-31 features D124 and D125 as catalytic residues, one acting as nucleophile and the other general acid/base, as has been shown by X-ray analysis of substrate complexes [79]. An exception is the GH76 homolog BT2949 from *B. thetaiotaomicron* VPI-5482^T^ where the second catalytic residue is a glutamate [80]. Multiple sequence alignment identified the aspartates D136 and D137 as catalytic residues in ShGH76 (Fig. 4B). A detailed description of the catalytic site is provided in the Supplementary Text.

### ShGH76 has a preference for linear α-1,6-mannans

We generated two double mutants, D136A/D137A (ShGH76^Ala^) and D136N/D137N (ShGH76^Asn^). Tests with ShGH76^Ala^ showed that it could indeed not hydrolyze Man7 (Fig. 5B, Bottom panel). However, ShGH76^Ala^ could still bind yeast α-mannan according to affinity PAGE analysis (Fig. 5C), whereas dot-blot assays did not show sufficient binding to yeast α-mannan (Supplementary Fig. S3B). ShGH76^Ala^ has apparently a low affinity or binding specificity for unmodified branched yeast α-mannan. We hence repeated the dot-blot assays with structurally modified (linear or debranched) yeast α-mannans (Supplementary Fig. S3C). Both ShGH76^Ala^ and ShGH76^Asn^ showed binding to linear yeast α-mannan, with ShGH76^Ala^ exhibiting higher affinity than ShGH76^Asn^, as assessed by blot color intensities.

### The ShGH76 active site is distinct from gut bacteria homologs

**Fig. 6:**
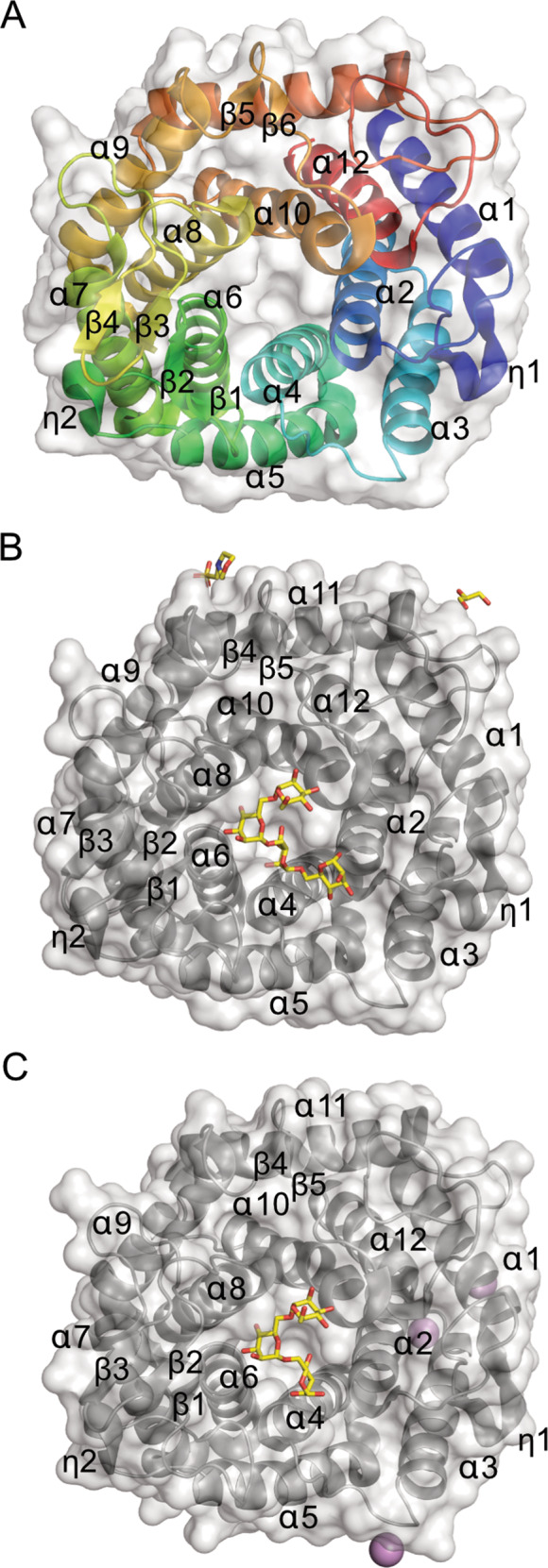
X-ray crystal structures of ShGH76 variants indicated classical single-domain (α/α)6-barrel fold. **A** X-ray crystal structure of ShGH76^WT^ displayed in cartoon format with a rainbow color scheme (NT = blue; CT = red) showing single-domain (α/α)_6_ fold viewed along the barrel axis with the active site in the center. **B** Overview of the ShGH76^Ala^ mutant structure in complex with mannotetrose (Man4: yellow stick) in cartoon format with a gray color showing single-domain (α/α)_6_ fold viewed along barrel axis having active site in the center. MES and glycerol are also indicated. **C** 3D-crystal structure of the ShGH76^Asn^ mutant in complex with mannotriose (Man3: yellow stick) displayed in cartoon format with gray color showing single-domain (α/α)_6_ fold viewed along the barrel axis with active site in the center. Three Ca^2+^ ions are shown as magenta spheres.

We solved the crystal structures of ShGH76^WT^ (apo-form), ShGH76^Asn^ (with Man3) and ShGH76^Ala^ (with Man4) at 2.0 Å, 1.47 Å and 1.9 Å resolution, respectively (Fig. 6A–C). The final R/R_free_ of ShGH76^WT^, ShGH76^Ala^ and ShGH76^Asn^ structures are 15.2/20.3, 11.8/17.2 and 16.2/19.9. There are no Ramachandran outliers in the models. The ShGH76^WT^ protein formed crystals belonging to the I 121 space group (Hermann-Mauguin notation) with three chains in an asymmetric unit (ASU), whereas the mutant protein ShGH76^Ala^ crystallized in the *P*22_1_2_1_ space group with one chain in an ASU, and the mutant ShGH76^Asn^ crystallized in the P12_1_1 space group with one chain in an ASU. In the apo-form, in total twelve α-helices and six β-strands were present, whereas five β-strands were present in both substrate-bound mutant forms.

**Fig. 7:**
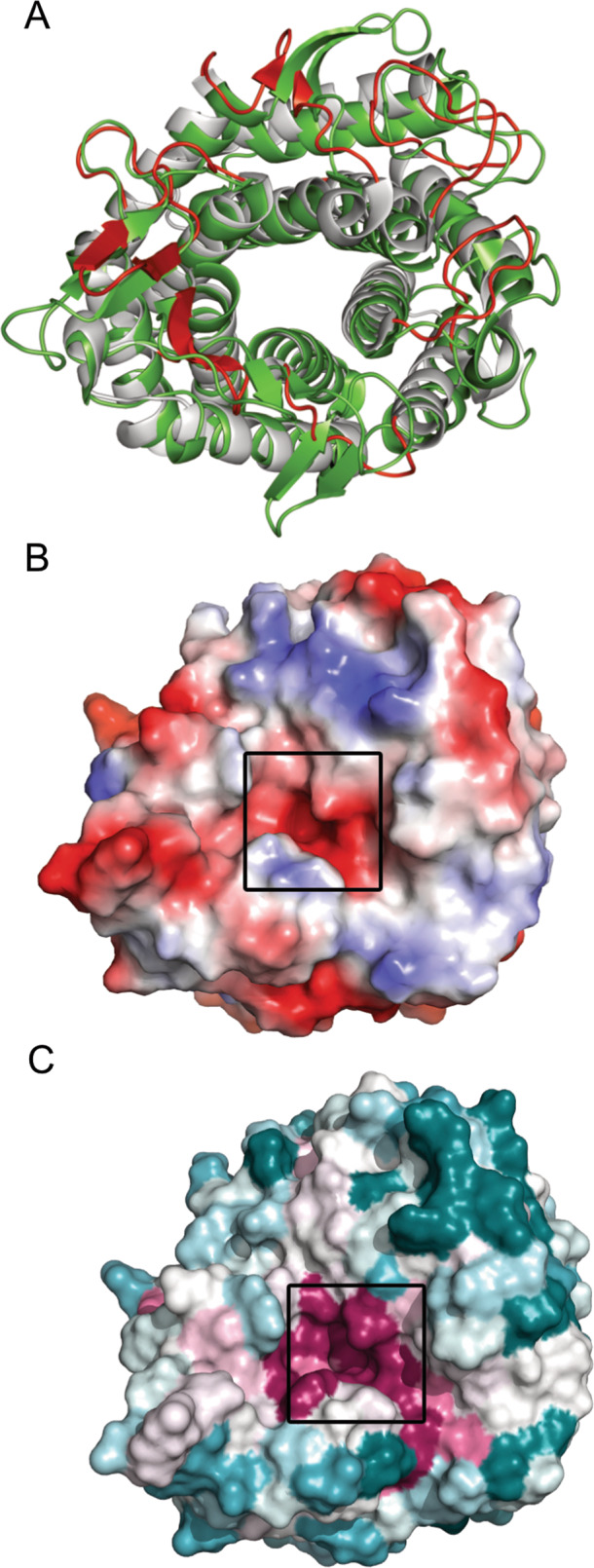
Highly conserved and negatively charged ShGH76 active site remains intact upon ligand binding. **A** Structural superimposition of ShGH76^WT^ (gray) and BT3792 (green, PDB ID: 4C1S) showing major variations in secondary structures highlighted in red color. **B** Electrostatic surface potential representation of ShGH76^WT^ showing the predominantly negatively charged active site (red: negatively charged; blue: positively charged). **C** Surface view of ShGH76^WT^ showing the highly conserved (magenta) active site pocket. Color intensity indicates conservation strength. The black box indicates the active site.

ShGH76^WT^ consists of three non-identical peptide chains (A, B and C), with root mean square deviations (r.m.s.d.) of 0.331 Å, 0.246 Å and 0.176 Å for chains pairs A/B; A/C; and chains B/C across matching C^α^ positions (further crystallographic data and refinement statistics of all three proteins are provided in Supplementary Table S5). The atomic models of the monomers of all three ShGH76 variants showed continuous peptide chains containing residues Asp54-Glu391. The residues 1–53 including the N-terminal 6x-histidine tag were not observed in the electron density maps of any of the structures. Despite a low sequence identity of <37%, the overall 3D structure of ShGH76 appeared well conserved with other known GH76 structures [PDB entries 4bok (*B. circulans*, unpublished work), 3k7x (*Listeria innocua*, unpublished work), 4mu9 (*B. thetaiotaomicron*, unpublished work), 4c1s [27] (*B. thetaiotaomicron*), 4v1s [80] (*B. thetaiotaomicron*), and 6u4z [65] (*B. thetaiotaomicron*)] (Supplementary Table S7). Structural and sequence alignments show that the catalytic residues Asp136 and Asp137 are highly conserved among these GH76 family members (Supplementary Fig. S4). Indeed, a structural comparison of ShGH76 and the six known family members produced respective r.m.s.d. values of 1.72, 1.41, 1.96, 1.68, 2.11, and 1.71 Å mapped across 305, 310, 301, 301, 301, and 304 C^α^ positions in each case (Supplementary Table S7). These r.ms.d value differences are mainly due to the secondary structure connecting flexible loops as observed in Fig. 7A. The tertiary structures of all three ShGH76 variants indicated a single-domain protein with a classical (α/α)_6_-barrel fold, with six α-helices forming a core that is surrounded by another six α-helices (Fig. 6A–C). A similar structural fold has been described for GH76 homologs from the human gut bacterium *B. thetaiotaomicron* VPI-5482^T^ [27, 80] and the soil bacterium *Bacillus circulans* TN-31 [79].

The ShGH76^WT^ active site pocket is predominantly negatively charged (Fig. 7B) - a trait that seems conserved based on surface electrostatic potential analyses with ConSurf (Fig. 7C). Besides negatively charged amino acids, the ShGH76 active site features seven aromatic amino acids (Trp90, Phe134, Trp140, Trp182, Phe256, Tyr258, Phe313). These are conserved among the investigated GH76 homologs (Supplementary Fig. S5A, B). Superimposition of bound Man5 from BcGH76 [79] suggests that Man5 does not fit the cavity of ShGH76^WT^ due to a steric clash of the Trp254 of ShGH76^WT^ (Supplementary Fig. S5C). Despite repeated attempts to co-crystallize with Man5, the crystals of the double mutants ShGH76^Ala^ (D136A-D137A) and ShGH76^Asn^ (D136N-D137N) were only obtained with bound Man3 and Man4 (Fig. 6B, C).

**Fig. 8:**
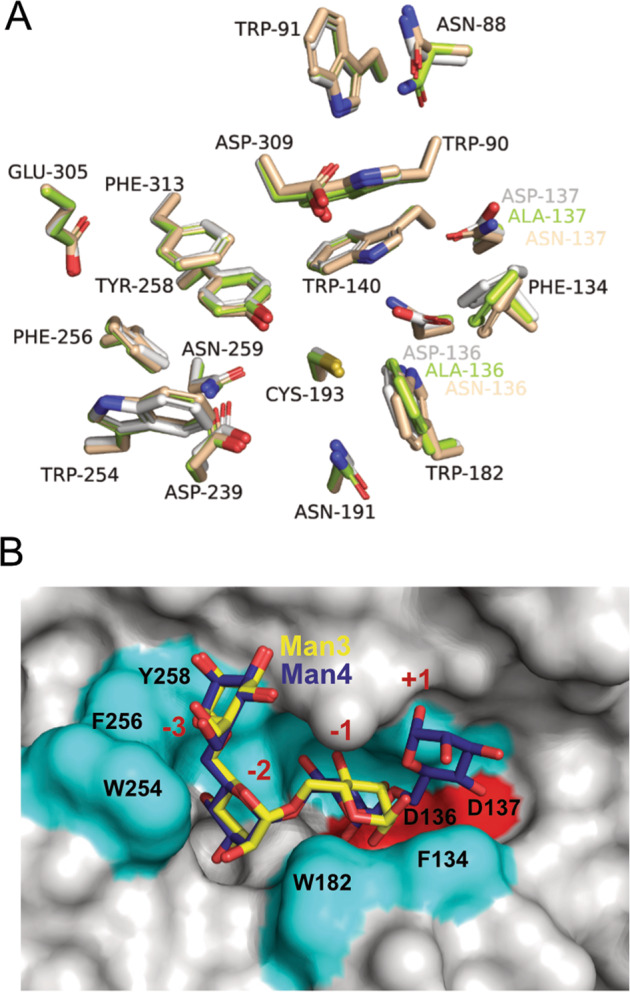
Ligand adopted a kinked conformation in the ShGH76 active site. **A** Superimposition of active site forming residues of all three ShGH76 structures, ShGH76^WT^ (gray), ShGH76^Ala^ (green) and ShGH76^Asn^ (wheat). **B** Surface-view representation of ShGH76^WT^ showing the ligand pocket and catalytic residues (red). The aromatic residues lining the active site are shown in cyan. The modeled Man3 (yellow) and Man4 (blue) are shown in stick representation.

During initial modeling, the electron density of the substrates was visible, so Man4 and Man3 could easily fit into the electron density (Supplementary Fig. S5D, E). The terminal mannose of the Man3 and Man4 ligands adopted a kinked conformation so as to occupy the active site, which has not been shown in GH76 structures (Fig. 8B). Superimposition of ShGH76^WT^ with ShGH76^Ala^ and ShGH76^Asn^ resulted in variations of 0.23 Å and 0.21 r.m.s.d, respectively, indicating minor loop movements on the protein surface but no global structural change (Supplementary Fig. S6A) as well as intact active site cores upon ligand binding (Supplementary Fig. S6B).

## Discussion

Metagenome and metaproteome studies have provided ample indications that bloom-associated bacteria in the southern North Sea degrade mannan-rich substrates [16, 17]. GH92 family exo-α-mannosidase genes are particularly frequent in North Sea *Bacteroidota*, and GH76 family endo-α-1,6-mannanase genes, while less frequent, have also been found in putative α-mannan-targeting PULs [19]. This is corroborated by the metagenome analyses in this study showing increases of GH92 and GH76 gene frequencies during North Sea spring algae blooms. Exo-acting GH92 family α-mannosidases can either cleave terminal mannose residues from side-chains of heterogeneous polysaccharides or from the backbones of genuine α-1,6-mannans. The latter, however, are more effectively cleaved by GH76 family endo-α-1,6-mannanases. Prevalence of GH92 over GH76 (Fig. 2) suggests that many glycans during blooms are decorated with mannose-containing side chains, but do not necessarily have α-1,6-mannan backbones. GH76 gene frequencies were highest in metagenomes from free-living bacteria. Less GH76 genes were found in metagenomes from particle-attached bacteria and none in sediment metagenomes. Likewise, sequenced *Salegentibacter* strains from sediments known to date do not possess GH76 genes. Thus, the GH76 target α-1,6-mannans seem to be readily degradable and rather short-lived after they are released to the water column.

ShGH76 shares only 19–37% sequence similarity with other known GH76 members and <27% sequence similarity with those from human gut bacteria (Supplementary Table S7). Despite this rather distant evolutionary relationship, all share the same overall quaternary structure, conserved active site motif, and α-1,6-mannanase activity. By comparison, ShGH76 sequence similarity to North Sea metagenome homologs was 57% to the closest sequence from the particle-associated and <30% to sequences from the free-living bacterioplankton fraction. A 50% sequence identity cutoff was recently used to define a glycoside hydrolase subfamily in which members share activity on the same substrate [81], but as illustrated for ShGH76 and its human gut bacteria homologs, the threshold for having the same function can be lower. Since the GH76 family is not diverse in terms of functions (α-1,6-mannanase and α-glucosidase are the only know functions) it is therefore reasonable to assume that a substantial fraction of the metagenomic GH76 enzymes function as endo-α-1,6-mannanases.

Bacterioplankton GH76 gene frequencies rise during North Sea spring algae blooms (Fig. 2). However, unlike fungi, marine algae are not known to possess mannans with α-1,6-linked backbones, but rather with α-1,3-backbones [33]. Distinct algae lineages in general are characterized by distinct glycans, such as ulvans (green algae), porphyrans and carrageenans (red algae), or laminarin and alginate (brown algae) [82]. Brown algae (*Phaeophyceae*) and diatoms (*Bacillariophyta*) belong to the *Stramenopiles* (heterokonts). Both contain laminarin, but diatoms are not known to possess alginate, even though a recent study indicates they might [83]. Lineage-specific glycans highlight that considerable recombinational barriers prevent their biosynthesis genes from being exchanged via lateral gene transfer. Presence of α-1,6-mannans in algae would hence be unexpected.

Provided there are no additional marine organisms for which it is not yet known that they do contain α-1,6-mannans, this leaves fungi as the most probable source. Helgoland is situated almost 50 km off the German coast, which is why α-1,6-mannans from terrestrial fungi are unlikely to reach there. Helgoland itself is a small, rocky, sparsely vegetated island without any major river outflow that is also unlikely to leak substantial amounts of fungal α-1,6-mannans to the surrounding sea. Hence marine fungi are the most likely candidates. These fungi have led a shadowy existence for a long time and research was sparse, but meanwhile more than 1900 species are known (https://marinefungi.org).

*Chytridiomycota* [84, 85], *Ascomycota* [86], *Basidiomycota*, and *Rozellomycota s.l*. are among the prominent clades of marine fungi that occur at Helgoland during phytoplankton blooms [87]. These are usually low in abundance in marine systems [88, 89], but can peak at times [90]. Such fluctuations result from changing environmental conditions [91], as in tidal mixing, wind forcing, sediment resuspension and periodic transport of water masses with riverine inputs [92]. Pelagic fungi thus represent a taxonomically diverse group of plankton (mycoplankton) in coastal areas that can process considerable amounts of biomass [93]. It has therefore recently been proposed to consider mycoplankton as a non-negligible active part of coastal microbial communities [87].

Opportunistic and saprophytic marine fungi can profit from the wealth of substrates released during algae blooms [94]. Likewise, parasitic clades such as *Chytridiomycota* can mass infect marine algae during blooms events, and have been suggested to play an important role in controlling the dynamic of phytoplankton populations [5]. For example, *Opisthosporidia* fungal diatom parasites are well known to occur in the North Sea [95]. Such population control is also exerted by fungal-like parasitic oomycetes [6] such as *Miracula helgolandica* [4, 96], which belong to the heterokonts and are not known to possess α-1,6-mannans.

Pelagic yeasts (*Basidomycota*) have been reported at Helgoland and showed seasonal variations with peak values in autumn during a three year observation period [97]. Labeled-substrate experiments provided initial evidences of assimilation of algal-derived particulate organic carbon by marine yeast [94, 98]. Considering the ubiquity of marine fungi and their preference for algal biomass, fungal α-1,6-mannans are expected at Helgoland island, particularly during phytoplankton blooms.

The organization of the ShGH76 PUL of strain Hel_I_6 is remotely similar to PULs of the human gut *B. thetaiotaomicron* that digest yeast and plant α-mannans. However, even GH76-containing PULs in different *Salegentibacter* species exhibit considerable variations, indicating that they do not target the exact same substrate. Thus, while ShGH76 does have a similar structure and similar mode of function as the GH76 enzymes of human gut bacteria, it likely targets differently structured α-1,6-mannans, which is also supported by the different accessory glycoside hydrolases in the ShGH76 PUL. Co-crystallizations with Man3 and Man4 show also a kinked position of the terminal non-reducing mannose residue that is unknown for any other GH76 so far (Fig. 8B). We speculate that this facilitates hydrolysis. Based on crystallographic data, we furthermore hypothesize that this bend allows for the enzyme to accommodate and hydrolyze longer substrates, even though we could not crystallize a ShGH76 mutant Man5 complex.

In *B. thetaiotaomicron* VPI-5482^T^, yeast α-mannan degradation is initiated by an extracellular GH99 family endo-α-1,2-mannosidase that debranches α-1,2-side chains from the α-1,6-mannan backbone [27]. A GH92 (BT2199) is then require to provide full access for two distinct extracellular GH76 enzymes to the α-1,6-mannan backbone, which cut it into transportable oligosaccharides [27]. Like BT2199 the GH92s present in strain Hel_I_6 could also prune the yeast mannan side chains, thereby facilitating the extracellular endo-α-1,6-mannanase GH76 to work on the mannan backbone. This multitude of initial products is then further digested by GH92, GH38, GH76 and GH125 family enzymes in the periplasm [27]. The mannan PUL of *Salegentibacter* by comparison has no GH99 homolog and only a single GH76 that prefers undecorated α-1,6-mannans but also functions on α-1,6-mannans with a low degree of branching (Supplementary Fig. S8B). Two of its six GH92 genes are predicted to be either extracellular or associated with flagella or fimbriae. One GH92 is located adjacent to the endo-α-1,6-mannanase GH76 encoding gene, suggesting a functional similarity to *B. thetaiotaomicron* VPI-5482^T^ [27].

ShGH76 is the first marine representative in the GH76 family of protein structures, and our data suggest that it targets marine fungal α-1,6-mannans. Turnover of such fungal organic matter does play a role in the marine carbon cycle, yet is currently not well understood. The structure and function of ShGH76 presented in this study thus provide a stepping stone towards a deeper understanding of this neglected part of the marine carbon cycle.

## Supplementary information

Supplementary information file

Figure S1

Figure S2

Figure S3

Figure S4

Figure S5

Figure S6

Figure S7

Figure S8

Figure S9

Table S1
