# Supplementary material for: Glycoside hydrolase from the GH76 family indicates that marine Salegentibacter sp. Hel_I_6 consumes alpha-mannan from fungi
Source: ISME J. 2022 Apr 12;16(7):1818–30. doi: 10.1038/s41396-022-01223-w (PMC9213526; doi:10.1038/s41396-022-01223-w)
Supplement: Supplementary file 1 — Supplementary information file [file 41396_2022_1223_MOESM1_ESM.pdf]

Supplementary Text

Glycoside hydrolase from the GH76 family indicates that marine *Salegentibacter* sp. Hel_I_6 consumes alpha-mannan from fungi

Vipul Solanki[1], Karen Krüger[1], Conor J. Crawford[2], Alonso Pardo-Vargas[2], José Danglad-Flores[2], Kim Le Mai Hoang[2,5], Leeann Klassen[3], D. Wade Abbott[3], Peter H. Seeberger[2], Rudolf I. Amann[1], Hanno Teeling[1*], Jan-Hendrik Hehemann[1,4*]

[1] Max Planck Institute for Marine Microbiology, Celsiusstraße 1, 28359 Bremen, Germany

[2] Max Planck Institute of Colloids and Interfaces, Am Mühlenberg 1, 14476 Potsdam, Germany

[3] Lethbridge Research and Development Centre, Agriculture and Agri-Food Canada, 5403 1st Avenue South, Lethbridge, Alberta, T1J 4B1, Canada

[4] University of Bremen, Center for Marine Environmental Sciences, MARUM, Leobener Straße 8, 28359 Bremen, Germany

[5] Present address: GlycoUniverse GmbH & Co KGaA, Am Mühlenberg 11, 14476 Potsdam, Germany

[*]corresponding authors

Jan-Hendrik Hehemann, e-mail: jheheman@mpi-bremen.de, Max Planck Institute for Marine Microbiology, Celsiusstrasse 1, 28359 Bremen, phone: +49 421 218 65775

Hanno Teeling, e-mail: hteeling@mpi-bremen.de, Max Planck Institute for Marine Microbiology, Celsiusstrasse 1, 28359 Bremen, phone: +49 421 2028 9760

This supporting information PDF file contains:

Supplementary Methods and Results with references

Supplementary Figures S1 – S9

Supplementary Tables S1 – S8

The Supplementary Table S1 is available as separate Microsoft Excel file.

Supplementary Methods

Synthesis of linear α-1,6-mannooligosaccharides

Table of Contents

Experimental Section 4

1. *General Materials and Methods* 4
2. *Preparation of Stock Solutions* 6
3. *Modules for Automated Synthesis* 6
4. *Post-synthesizer Manipulations* 8
5. *Oligosaccharide Deprotection* 9
6. *Purification* 9

Compound Characterisation 11

NMR Data 25

Experimental Section

1. *General Materials and Methods*

Reagent grade chemicals were used for the synthesis of linear α-1,6-mannooligosaccharide synthesis unless noted otherwise. All building blocks used were purchased from GlycoUniverse (Potsdam, Germany). Automated syntheses were performed on a home-built synthesizer developed at the Max Planck Institute of Colloids and Interfaces (Golm, Germany). Merrifield resin LL (100-200 mesh, Novabiochem™) was modified and used as solid support. Analytical thin-layer chromatography (TLC) was performed on Merck silica gel 60 F254 plates (0.25 mm). Compounds were visualized by UV irradiation or dipping the plate in a p-anisaldehyde (PAA) solution. Flash column chromatography was carried out by using forced flow of the indicated solvent on Fluka silica gel 60 M (0.04–0.063 mm). Analysis and purification by normal and reverse phase HPLC was performed using an Agilent 1200 series instrument. Products were lyophilized using a Christ Alpha 2-4 LD plus freeze dryer. ^{1}H-, ^{13}C- and HSQC-NMR spectra were recorded on Varian 400-MR (400 MHz), Varian 600-MR (600 MHz), or Bruker Biospin AVANCE700 (700 MHz) spectrometers. Spectra were recorded in $CDCl_3$ by using the solvent residual peak chemical shift as the internal standard ($CDCl_3$: 7.26 ppm ^{1}H, 77.0 ppm ^{13}C) or in D_2O using the solvent as the internal standard in ^{1}H-NMR (D_2O: 4.79 ppm ^{1}H) and a D_6-acetone spike as the internal standard in ^{13}C-NMR (acetone in D_2O: 30.89 ppm ^{13}C) unless otherwise stated. High resolution mass spectra were obtained using a 6210 ESI-TOF mass spectrometer (Agilent) and a MALDI-TOF Autoflex™ (Bruker). MALDI and ESI mass spectra were run on IonSpec Ultima instruments.

Solvents used for dissolving building block and preparing the activator, TMSOTf and capping solutions were taken from an anhydrous solvent system (jcmeyer-solvent systems). Other solvents used were HPLC grade. The building blocks were co-evaporated three times with toluene

and dried 2 h under high vacuum before use. Activator, deprotection, acidic wash, capping and building block solutions were freshly prepared and kept under argon during the automation run. All yields of products obtained by AGA were calculated based on resin loading. Resin loading was determined by performing one glycosylation (Module C) with ten equivalents of building block followed by DBU promoted Fmoc-cleavage and determination of dibenzofulvene production by measuring its UV absorbance.

2. *Preparation of Stock Solutions*

- Building block: building block was dissolved in 1 mL dichloromethane (DCM).
- Activator solution: Recrystallized NIS (1.56 g) was dissolved in 60 mL of a 2:1 mixture of anhydrous DCM and anhydrous dioxane. Then trifluoromethanesulfonic acid (67 µL) was added. The solution was kept at 0 °C for the duration of the automation run.
- Fmoc deprotection solution: A solution of 20% piperidine in dimethylformamide (DMF) (v/v) was prepared.
- TMSOTf solution: Trimethylsilyl trifluoromethanesulfonate (TMSOTf) (0.9 mL) was added to DCM (90 mL).
- Capping solution: A solution of 10% acetic anhydride (Ac_2O) and 2% methanesulfonic acid (MsOH) in anhydrous DCM (v/v) was prepared.

3. *Modules for Automated Synthesis*

Module A — Resin Preparation for Synthesis (20 min)

All automated syntheses were performed on 45 mg of resin with a loading of 0.30 mmol/g scale. Resin was placed in the reaction vessel and swollen in DCM for 20 min while the reaction vessel temperature adjusted to -20 °C prior to synthesis. During this time, all reagent lines required for the synthesis were washed and primed. Before the first glycosylation, the resin was washed with the DMF, tetrahydrofuran (THF), and DCM (three times each with 2 mL for 25 s). This step was conducted as first step in every synthesis.

Module B —Acidic Wash with TMSOTf Solution (3 min)

The resin was swollen in 2 mL DCM and the temperature of the reaction vessel was adjusted to -20 °C. Upon reaching the temperature, TMSOTf solution (1 mL) was added drop wise to the reaction vessel. After bubbling for 3 min, the acidic solution was drained, and the resin was washed with 2 mL DCM for 25 s.

Module C —Thioglycoside Glycosylation (20-60 min)

The building block solution (6.5 eq. of BB in 1 mL of DCM per glycosylation) was delivered to the reaction vessel. After the set temperature (T1, -20 °C) was reached, the reaction was started by drop wise addition of the activator solution (1.0 mL, excess), the reagents were incubated for 8 min. The glycosylation was performed by increasing the temperature (4 °C/min) to -10 °C for 10-20 min (depending on oligosaccharide length). After completion of the reaction, the solution was drained and the resin was washed with DCM, DCM:dioxane (1:2, 3 mL for 20 s) and DCM (twice, each with 2 mL for 25 s).

Module D —Capping (15 min)

The resin was washed with DMF (twice with 2 mL for 25 s) and the temperature of the reaction vessel was adjusted to 25 °C. Pyridine solution 2 mL (10% in DMF) was delivered into the reaction vessel. After 1 min, the reaction solution was drained, and the resin washed with DCM (three times with 3 mL for 25 s). The capping solution 4 mL was delivered into the reaction vessel (the temperature adjusted to 25 °C). After 8 min, the reaction solution was drained, and the resin washed with DCM (thrice with 3 mL for 25 s).

Module E — Fmoc Deprotection (1 min)

The resin was washed with DMF (thrice with 2 mL for 25 s) and the temperature of the reaction vessel was adjusted to 60 °C. Fmoc deprotection solution (2 mL) was delivered into the reaction vessel. After 1 min, the reaction solution was drained, and the resin washed with DMF (three times with 3 mL for 25 s) and DCM (five times each with 2 mL for 25 s). The temperature of the reaction vessel was reduced to -20 °C for the next module.

4. *Post-synthesizer Manipulations*

Cleavage from Solid Support

After automated synthesis, the oligosaccharides were cleaved from the solid support using a continuous-flow photo reactor. The Vapourtec E-Series UV-150 Photoreactor Flow Chemistry System with mercury lamp was employed. The resin, suspended in dichoromethane, was loaded into a plastic syringe. The suspension was pumped using a syringe pump (PHD2000, Harvard Apparatus) at 1 mL/min through a 20 mL reactor, constructed of 1/8 inch O.D. FEP tubing. The temperature of the photoreactor was maintained at 20 °C. For selected cleavages, the mercury lamp was replaced by a LED 365 nm UV lamp.

5. *Oligosaccharide Deprotection*

Module G — Methanolysis

The protected oligosaccharide was dissolved in MeOH:DCM (1.5 mL,1:1). NaOMe in MeOH (0.1 mL of 0.5M solution) was added to the solution and stirred at room temperature. After 12 h, the solution was neutralized with Amberlite IR-120 (H+ form) resin, filtered and concentrated *in vacuo.* The crude compound was used for hydrogenolysis without further purification.

Module H — Hydrogenolysis with Pd/C

The crude compound obtained from *Module G* was dissolved in 2 mL of THF:^{t}BuOH:H_2O (60:10:30) and 200mg 5% Pd/C (Strem Chemicals) was added. The reaction was stirred in a hydrogen balloon (1, 2).

6. *Purification*

Solvent was evaporated in vacuo and the crude products were dissolved in 1:1 mixture of hexane and ethyl acetate and analysed using analytical HPLC (DAD1F, 280 nm). Pure compounds were afforded by preparative HPLC (Agilent 1200 Series spectrometer).

Method A (YMC-Diol-300 column, 150 x 4.6 mm) flow rate of 1.0 mL / min with Hex – 20% EtOAc as eluent [isocratic 20% EtOAc (5 min), linear gradient to 55% EtOAc (45 min), linear gradient to 100% EtOAc (5 min)].

Method B (YMC-Diol-300 column, 150 x 20 mm) flow rate of 15 mL / min with Hex – 20% EtOAc as eluent [isocratic 20% EtOAc (5 min), linear gradient to 55% EtOAc (45 min), linear gradient to 100% EtOAc (5 min)].

Method C (Synergi Hydro RP18 column, 250 x 4.6 mm) flow rate of 1.0 mL / min with H2O (0.1% formic acid) as eluents [isocratic (5 min), linear gradient to 10% ACN (30 min), linear gradient to 100% ACN (5 min)].

Method D (Synergi Hydro RP18 column, 250 x 10 mm) flow rate of 4.0 mL / min with H2O (0.1% formic acid) as eluents [isocratic (5 min), linear gradient to 10% ACN (30 min), linear gradient to 100% ACN (5 min)].

Method E (YMC-Diol-300 column, 150 x 4.6 mm) flow rate of 1.0 mL / min with Hex – 35% EtOAc as eluents [isocratic 35% EtOAc (5 min), linear gradient to 60% EtOAc (5 min), linear gradient to 60% EtOAc (30 min), linear gradient to 100% EtOAc (5 min)].

Compound Characterisation

α-D-Mannopyranosyl-(1→6)-D-mannopyranose (1)

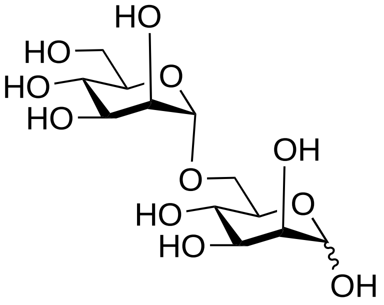

Cycles	Module	Conditions
	A. Resin Preparation	
	B. Acidic wash with TMSOTf solution	
**2**	C. Thioglycoside Glycosylation	**BB1** 6.5 eq., -20 °C, for 8 min, -10 °C for10 min
	D. Capping	
	E. Fmoc Deprotection	

Cleavage from solid support as described in the post-synthesizer manipulation section, followed by purification using preparative HPLC (Method B) afforded the protected derivative of **1** (5 mg, 31%). Deprotection as described in Module G and H, followed by purification using preparative HPLC (Method D) afforded compound **1** as a mixture of α and β isomers (1.0 mg, 3.2 μmol, 60% over two steps).

RP-HPLC of 1 (ELSD trace, Method E, tR = 16.1 min)

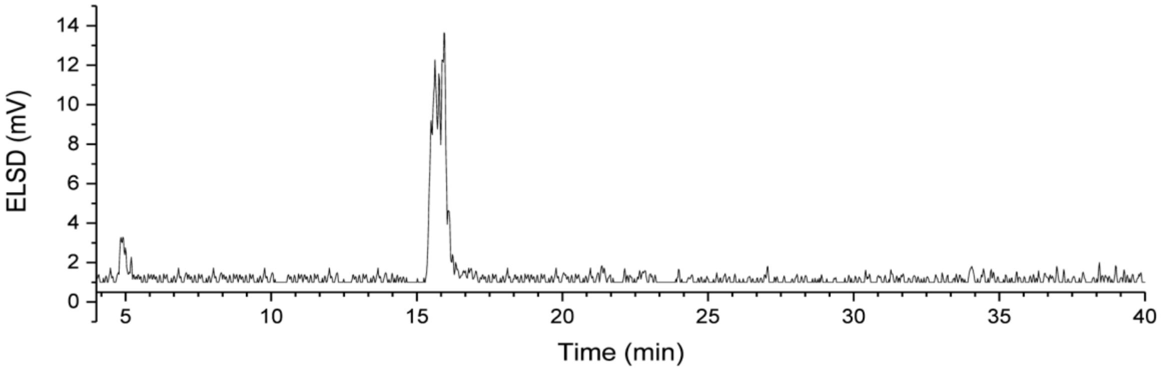

^{1}H NMR (700 MHz, D_2O) δ 8.59 (dd, *J* = 2.4, 1.2 Hz, 1H), 5.30 (d, *J* = 2.3 Hz, 1H), 5.06 – 5.02 (m, 3H), 4.13 (qt, *J* = 4.6, 2.7 Hz, 3H), 4.11 – 4.06 (m, 4H), 4.06 – 3.98 (m, 10H), 3.98 – 3.94 (m, 1H), 3.94 – 3.91 (m, 3H), 3.91 – 3.87 (m, 5H), 3.87 – 3.76 (m, 10H), 3.75 – 3.67 (m, 1H), 3.67 – 3.64 (m, 1H). **^{13}C NMR** (176 MHz, D_2O) δ 99.9, 99.7, 94.3, 93.9, 74.4, 73.3, 72.8, 72.8, 71.2, 71.0, 70.7, 70.6, 70.5, 70.1, 70.0, 69.3, 69.1, 68.7, 66.9, 66.8, 66.6, 66.0, 63.3, 61.0. **HRMS** $[M+Na]^+$ *m/z* Calcd for $C_{12}H_{22}O_{11}Na$, 365.1054; found, 365.1110.

α-D-Mannopyranosyl-(1→6)-α-D-mannopyranosyl-(1→6)-D-mannopyranose (2)

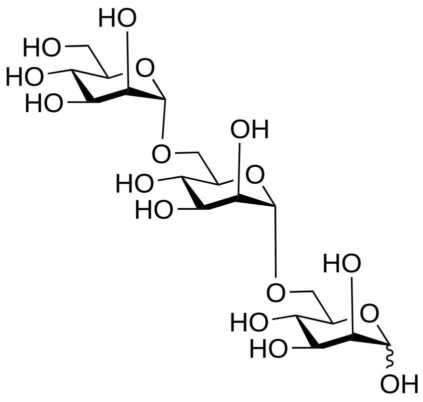

Cycles	Module	Conditions
	A. Resin Preparation	
3	B. Acidic wash with TMSOTf solution	
	C. Thioglycoside Glycosylation	**BB1** 6.5 eq., -20 °C, for 8 min, -10 °C for 10 min
	D. Capping	
	E. Fmoc Deprotection	

Cleavage from solid support as described in post-synthesizer manipulations section, followed by purification using preparative HPLC (Method B) afforded the protected derivative of **2** (5 mg, 31%). Deprotection as described in Module G and H, followed by purification using preparative HPLC (Method D) afforded compound **2** as a mixture of α and β isomers (2.0 mg, 6.3 µmol, 40% over two steps).

RP-HPLC of 2 (ELSD trace, Method E, tR = 18.5 min)

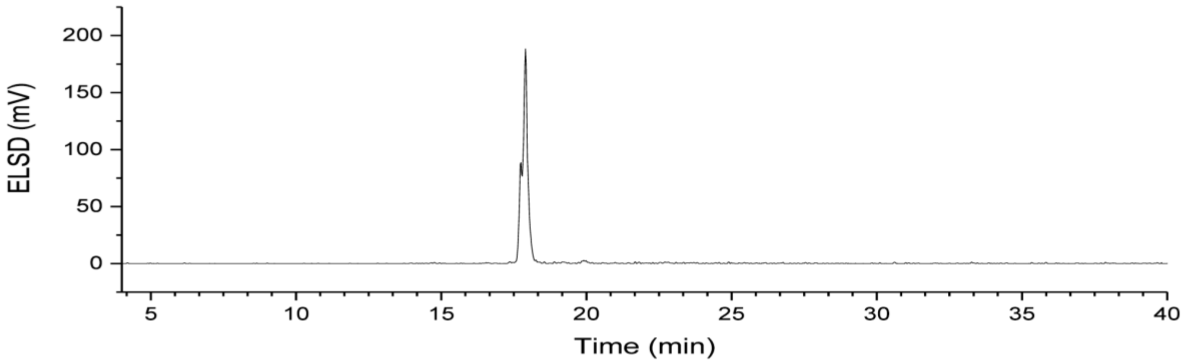

^{1}H NMR (700 MHz, D_2O) δ 5.18 (s, 1H), 4.92 (s, 2H), 4.91 (s, 1H), 4.89 (s, 2H), 4.03 – 3.95 (m, 7H), 3.97 – 3.93 (m, 4H), 3.93 – 3.87 (m, 11H), 3.87 – 3.83 (m, 17H), 3.82 – 3.79 (m, 5H), 3.79

– 3.76 (m, 5H), 3.77 – 3.74 (m, 7H), 3.74 – 3.71 (m, 9H), 3.70 – 3.69 (m, 1H), 3.68 – 3.64 (m, 6H). **^{13}C NMR** (176 MHz, D_2O) δ 100.0, 99.5, 72.8, 70.9, 70.7, 70.1, 69.3, 69.1, 69.0, 68.7, 66.9, 66.8, 65.8, 63.3, 61.0. **HRMS** $[M+Na]^+$ *m/z* Calcd for $C_{18}H_{32}O_{16}Na$, 527.1599; found, 527.1583.

α-D-Mannopyranosyl-(1→6)-α-D-mannopyranosyl-(1→6)-α-D-mannopyranosyl-(1→6)-D-mannopyranose (3)

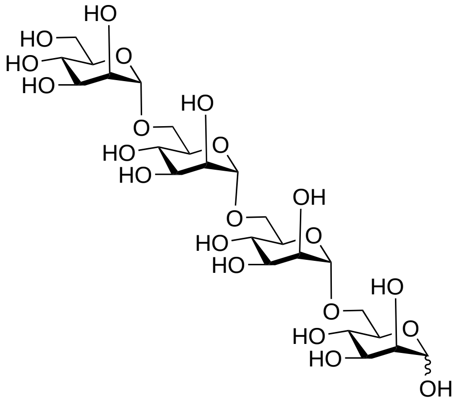

Cycles	Module	Conditions
	A. Resin Preparation	
4	B. Acidic wash with TMSOTf solution	
	C. Thioglycoside Glycosylation	**BB1** 6.5 eq., -20 °C, for 8 min, -10 °C for 10 min
	D. Capping	
	E. Fmoc Deprotection	

Cleavage from solid support as described in post-synthesizer manipulations section, followed by purification using preparative HPLC (Method B) afforded the protected derivative of **3** (5 mg, 31%). Deprotection as described in Module G and H, followed by purification using preparative HPLC (Method D) afforded compound **3** as a mixture of α and β isomers (2.0 mg, 5.0 μmol, 40% over two steps).

RP-HPLC of 3 (ELSD trace, Method E, tR = 19.3 min)

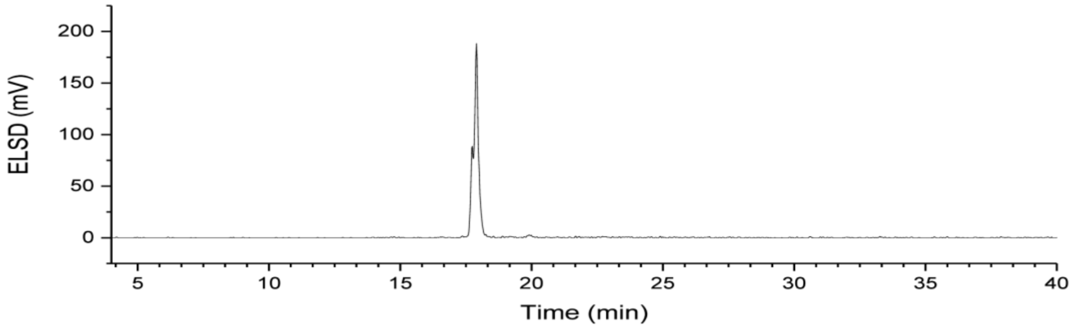

^{1}H NMR (400 MHz, D_2O) δ 5.12 (d, *J* = 1.8 Hz, 2H), 4.87 (s, 2H), 4.86 (s, 2H), 4.85 (d, *J* = 3.2 Hz, 10H), 3.99 – 3.92 (m, 13H), 3.89 (dq, *J* = 7.2, 4.7, 4.3 Hz, 9H), 3.84 (d, *J* = 1.6 Hz, 3H), 3.81 (dt, *J* = 3.7, 1.7 Hz, 8H), 3.79 – 3.76 (m, 10H), 3.76 – 3.72 (m, 7H), 3.71 – 3.67 (m, 11H), 3.65 (dd, *J* = 5.2, 1.5 Hz, 4H), 3.65 – 3.56 (m, 6H). **^{13}C NMR** (101 MHz, D_2O) δ 99.4, 99.4, 99.1, 99.1, 94.1, 93.7, 74.0, 73.1, 72.6, 71.0, 70.7, 70.6, 70.5, 70.5, 70.4, 70.3, 70.3, 69.8, 69.8, 66.6, 66.5, 66.5, 66.4, 66.3, 65.6, 65.4, 65.3, 60.8. **HRMS** $[M+Na]^+$ *m/z* Calcd for $C_{24}H_{42}O_{21}Na$, 689.2140; found, 689.2111.

α-D-Mannopyranosyl-(1→6)-α-D-mannopyranosyl-(1→6)-α-D-mannopyranosyl-(1→6)-α-D-mannopyranosyl-(1→6)-D-mannopyranose (4)

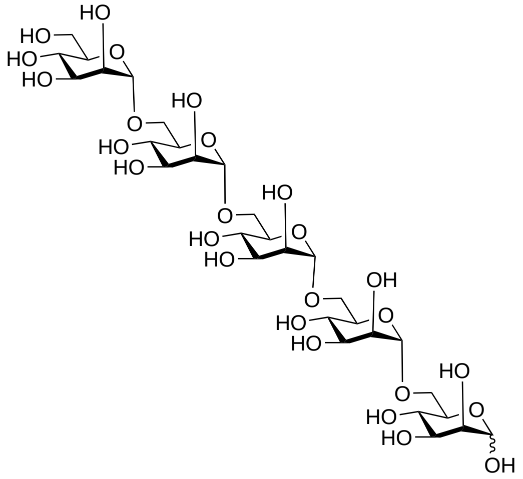

Cycles	Module	Conditions
	A. Resin Preparation	
**5**	B. Acidic wash with TMSOTf solution	
	C. Thioglycoside Glycosylation	**BB1** 6.5 eq., -20 °C, for 8 min, -10 °C for 10 min
	D. Capping	
	E. Fmoc Deprotection	

Cleavage from solid support as described in post-synthesizer manipulations section, followed by purification using preparative HPLC (Method B) afforded the protected derivative of **4** (5 mg, 31%). Deprotection as described in Module G and H, followed by purification using preparative HPLC (Method D) afforded compound **4** as a mixture of α and β isomers (2.4 mg, 2.9 µmol, 60% over two steps).

RP-HPLC of 4 (ELSD trace, Method E, tR = 19.8 min)

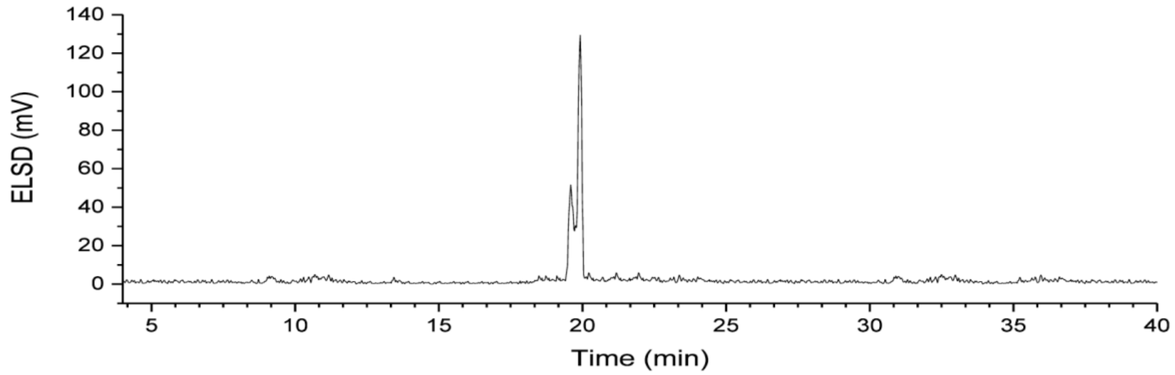

^{1}H NMR (600 MHz, D_2O) δ 5.18 (s, 1H), 4.94 – 4.89 (m, 8H), 4.01 (q, *J* = 4.2, 3.3 Hz, 8H), 3.96 (dq, *J* = 10.2, 6.4, 5.5 Hz, 11H), 3.92 (d, *J* = 2.0 Hz, 1H), 3.90 (d, *J* = 1.9 Hz, 1H), 3.91 – 3.82 (m, 16H), 3.81 (s, 2H), 3.79 (d, *J* = 5.8 Hz, 3H), 3.76 (d, *J* = 7.9 Hz, 3H), 3.73 (d, *J* = 10.0 Hz, 7H), 3.67 (s, 1H). **^{13}C NMR** (151 MHz, D_2O) δ 99.5, 99.4, 99.2, 99.2, 99.2, 94.1, 93.7, 81.6, 74.0, 73.1, 72.6, 71.1, 70.7, 70.7, 70.6, 70.6, 70.6, 70.6, 70.5, 70.4, 70.4, 70.4, 69.9, 69.8, 69.8, 69.8, 66.6, 66.5, 66.5, 66.5, 66.5, 66.3, 65.6, 65.5, 65.5, 65.4, 60.8. **HRMS** $[M+Na]^+$ *m/z* Calcd for $C_{30}H_{52}O_{26}Na$, 851.2644; found, 851.2639.

4-(Hydroxymethyl)-benzyl (2-O-benzoyl-3,4-di-O-benzyl-α-D-mannopyranosyl-(1→6)-2-O-benzoyl-3,4-di-O-benzyl-α-D-mannopyranosyl-(1→6)-2-O-benzoyl-3,4-di-O-benzyl-α-D-mannopyranosyl-(1→6)-2-O-benzoyl-3,4-di-O-benzyl-α-D-mannopyranosyl-(1→6)-2-O-benzoyl-3,4-di-O-benzyl-α-D-mannopyranosyl-(1→6)-2-O-benzoyl-3,4-di-O-benzyl-D- mannopyranoside (5)

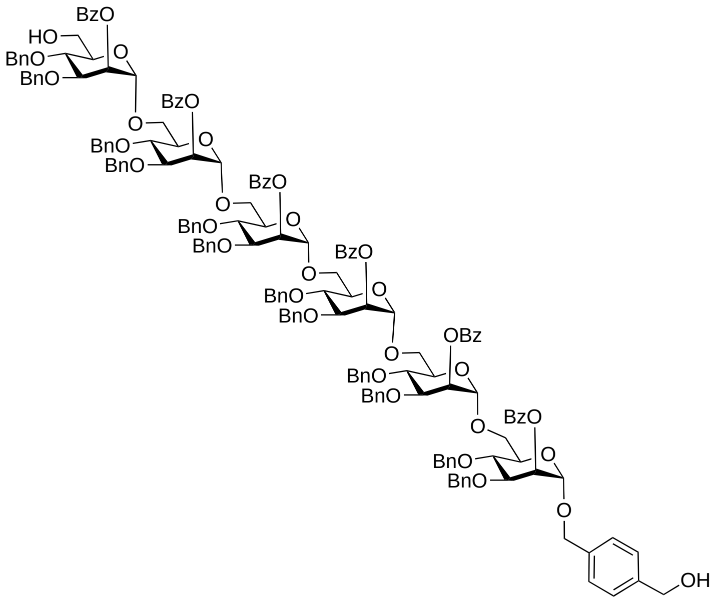

Cycles	Module	Conditions
	A. Resin Preparation	
**6**	B. Acidic wash with TMSOTf solution	
	C. Thioglycoside Glycosylation	**BB1** 6.5 eq., -20 °C, for 8 min, -10 °C for 10 min
	D. Capping	
	E. Fmoc Deprotection	

Cleavage from solid support as described in post-synthesizer manipulations section, followed by purification using preparative HPLC (**Method B**) afforded compound **5** (15 mg, 38% from resin).

^{1}H NMR (600 MHz, $CDCl_3$) δ 8.18 – 8.13 (m, 8H), 8.08 (dd, *J* = 8.0, 5.8 Hz, 5H), 7.58 (t, *J* = 7.4 Hz, 1H), 7.53 – 7.42 (m, 19H), 7.33 – 7.27 (m, 10H), 7.26 – 7.22 (m, 6H), 7.22 – 7.14 (m, 27H), 7.13 – 7.08 (m, 11H), 5.84 – 5.74 (m, 6H), 5.67 (t, *J* = 2.6 Hz, 1H), 5.10 – 5.01 (m, 6H), 4.97 – 4.94 (m, 1H), 4.92 – 4.84 (m, 7H), 4.84 – 4.75 (m, 7H), 4.69 (dd, *J* = 30.6, 11.6 Hz, 3H), 4.64 – 4.57 (m, 4H), 4.57 – 4.32 (m, 15H), 4.16 – 3.85 (m, 18H), 3.85 – 3.68 (m, 7H), 3.68 – 3.56 (m, 6H), 3.56 – 3.51 (m, 2H), 3.46 (dd, *J* = 11.1, 5.4 Hz, 2H). **^{13}C NMR** (151 MHz, $CDCl_3$) δ 165.8, 165.7, 165.6, 165.6, 165.5, 140.9, 138.6, 138.6, 138.6, 138.4, 138.3, 138.0, 137.7, 137.7, 137.7, 137.6, 137.6, 136.1, 133.4, 133.4, 130.1, 130.1, 130.0, 130.0, 129.9, 128.7, 128.6, 128.6, 128.4, 128.4, 128.4, 128.3, 128.3, 128.3, 128.2, 128.2, 128.1, 128.1, 127.8, 127.8, 127.7, 127.7, 127.7, 127.5, 127.5, 127.4, 127.4, 127.4, 127.3, 127.2, 127.2, 98.6, 98.6, 98.5, 98.3, 98.2, 97.0, 78.8, 78.4, 78.3, 78.3, 78.3, 77.8, 77.3, 77.1, 76.9, 75.3, 75.2, 75.2, 75.1, 75.1, 74.3, 74.1, 73.9, 73.9, 73.8, 72.2, 71.8, 71.5, 71.5, 71.4, 71.4, 71.3, 71.2, 71.1, 71.1, 71.0, 69.2, 69.1, 68.7, 68.6, 68.5, 68.5, 66.2, 65.9, 65.8, 65.6, 65.1, 61.9. **HRMS** $[M+Na]^+$ *m/z* Calcd for $C_{170}H_{116}O_{38}Na$, 2839.092; found, 2839.098.

α-D-Mannopyranosyl-(1→6)-α-D-mannopyranosyl-(1→6)-α-D-mannopyranosyl-(1→6)-α-D-mannopyranosyl-(1→6)-α-D-mannopyranosyl-(1→6)-D-mannopyranose (6)

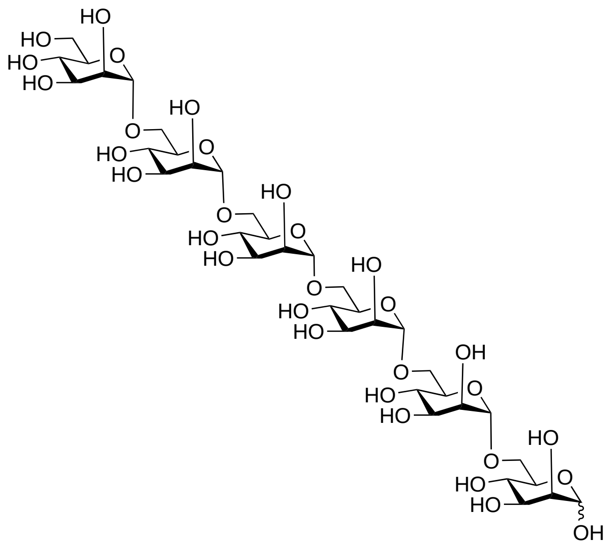

Deprotection of **5** as described in Module G and H, followed by purification using preparative HPLC (Method D) afforded compound **6** as a mixture of α and β isomers (3 mg, 3.6 μmol, 58% over two steps).

RP-HPLC of 6 (ELSD trace, Method A tR = 20.5 min)

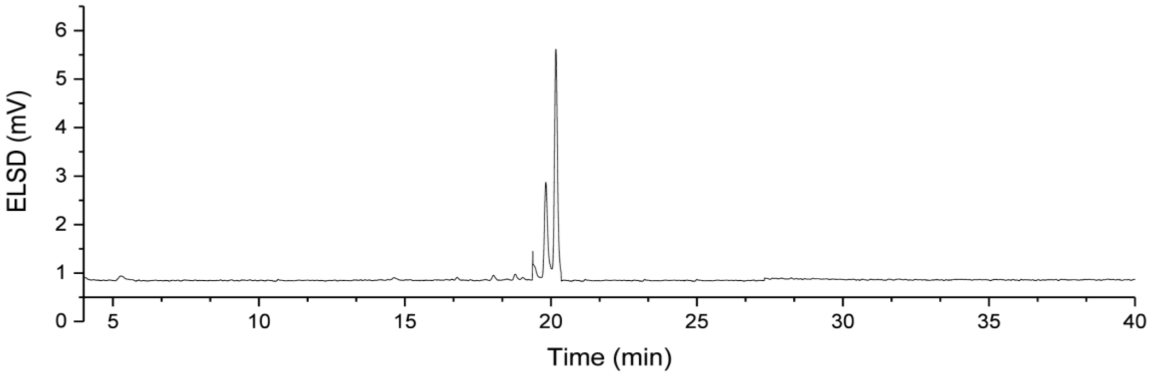

^{1}H NMR (700 MHz, D_2O) δ 5.31 (s, 1H), 5.07 – 5.02 (m, 10H), 4.15 – 4.12 (m, 10H), 4.12 – 4.05 (m, 13H), 4.05 – 4.00 (m, 3H), 4.00 – 3.95 (m, 21H), 3.95 – 3.90 (m, 6H), 3.92 – 3.88 (m, 3H), 3.90 – 3.84 (m, 10H), 3.83 – 3.77 (m, 4H). **^{13}C NMR** (176 MHz, D_2O) δ 99.7, 99.6, 99.4, 94.3,

93.9, 74.3, 73.3, 72.8, 71.3, 70.9, 70.9, 70.9, 70.8, 70.7, 70.6, 70.1, 70.0, 70.0, 69.3, 66.9, 66.86, 66.80, 66.6, 65.9, 65.86, 65.81, 65.75, 65.73, 63.3, 61.0, 55.5. **HRMS** $[M+Na]^+$ *m/z* Calcd for $C_{170}H_{116}O_{38}Na$, 1013.3182; found, 1013.3172.

4-(Hydroxymethyl)-benzyl (2-O-benzoyl-3,4-di-O-benzyl-α-D-mannopyranosyl)-(1→6)-2-O-benzoyl-3,4-di-O-benzyl-α-D-mannopyranosyl-(1→6)-2-O-benzoyl-3,4-di-O-benzyl-α-D-mannopyranosyl-(1→6)-2-O-benzoyl-3,4-di-O-benzyl-α-D-mannopyranosyl-(1→6)-2-O-benzoyl-3,4-di-O-benzyl-α-D-mannopyranosyl-(1→6)-2-O-benzoyl-3,4-di-O-benzyl-α-D-mannopyranosyl-(1→6)-2-O-benzoyl-3,4-di-O-benzyl-D-mannopyranoside (7)

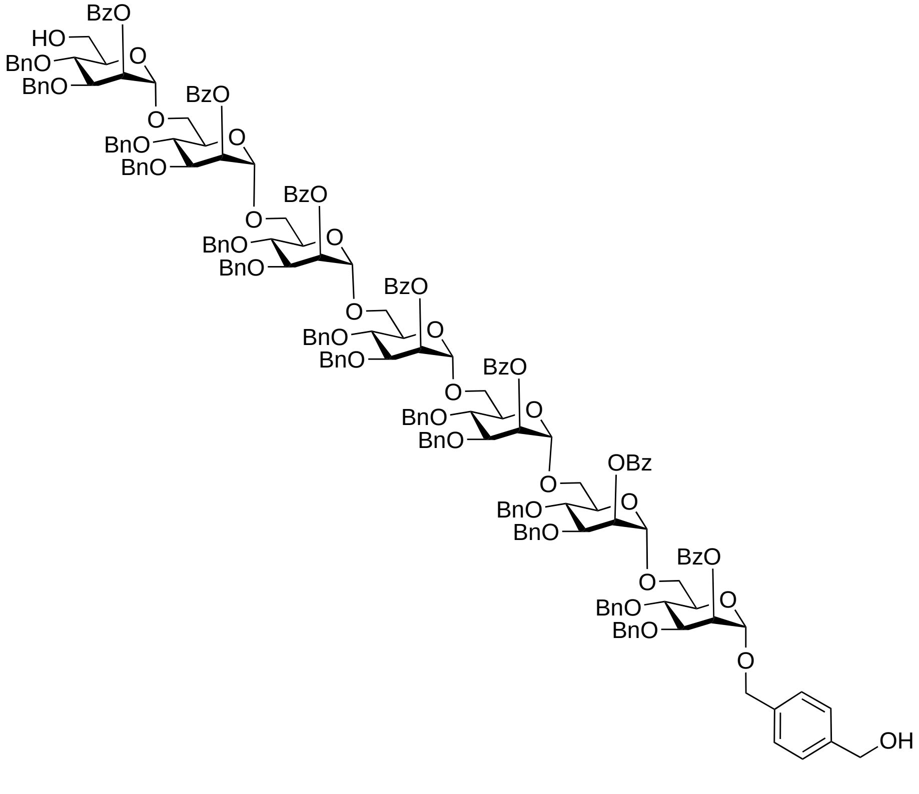

Cycles	Module	Conditions
	A. Resin Preparation	
7	B. Acidic wash with TMSOTf solution	
	C. Thioglycoside Glycosylation	**BB1** 6.5 eq., -20 °C, for 8 min, -10 °C for1 min
	D. Capping	
	E. Fmoc Deprotection	

Cleavage from solid support as described in the post-synthesizer manipulations section, followed by purification using preparative HPLC (**Method B**) afforded compound **7** (29 mg, 63% from resin).

^{1}H NMR (600 MHz, $CDCl_3$) δ 8.16 (ddt, *J* = 7.9, 4.9, 3.0 Hz, 9H), 8.08 (ddt, *J* = 8.0, 6.0, 2.5 Hz, 4H), 7.62 – 7.55 (m, 1H), 7.48 (dqd, *J* = 15.3, 7.6, 5.0 Hz, 19H), 7.34 – 7.03 (m, 55H), 5.82 (dq, *J* = 7.5, 2.5 Hz, 4H), 5.80 – 5.77 (m, 1H), 5.76 (t, *J* = 2.5 Hz, 1H), 5.67 (dd, *J* = 3.3, 1.8 Hz, 1H), 5.10 – 5.01 (m, 6H), 4.95 (d, *J* = 1.8 Hz, 1H), 4.92 – 4.69 (m, 15H), 4.69 – 4.57 (m, 4H), 4.57 – 4.31 (m, 15H), 4.16 – 3.86 (m, 17H), 3.85 – 3.70 (m, 5H), 3.73 – 3.62 (m, 5H), 3.64 – 3.50 (m, 5H), 3.46 (td, *J* = 11.8, 1.9 Hz, 3H). **^{13}C NMR** (151 MHz, $CDCl_3$) δ 165.8, 165.7, 165.6, 165.5, 140.9, 138.6, 138.6, 138.5, 138.4, 138.3, 138.0, 137.7, 137.7, 137.7, 137.6, 137.6, 136.1, 133.4, 133.3, 130.1, 130.1, 130.0, 130.0, 129.9, 128.7, 128.7, 128.6, 128.6, 128.6, 128.4, 128.4, 128.4, 128.3, 128.3, 128.2, 128.2, 128.1, 128.1, 127.8, 127.8, 127.7, 127.7, 127.7, 127.5, 127.5, 127.4, 127.4, 127.3, 127.2, 127.2, 127.2, 98.6, 98.5, 98.3, 98.2, 97.0, 78.8, 78.4, 78.3, 78.3, 78.3, 77.8, 77.3, 77.1, 76.9, 75.3, 75.2, 75.2, 75.1, 75.1, 74.3, 74.1, 73.9, 73.8, 73.8, 72.2, 71.8, 71.5, 71.4,

71.4, 71.3, 71.2, 71.1, 71.1, 71.0, 69.2, 69.1, 68.7, 68.6, 68.5, 66.2, 65.8, 65.5, 65.1, 61.9. **HRMS** $[M+Na]^+$ *m/z* Calcd for $C_{197}H_{192}O_{44}Na$, 3286.2752; found, 3286.2741.

α-D-Mannopyranosyl-(1→6)-α-D-mannopyranosyl-(1→6)-α-D-mannopyranosyl-(1→6)-α-D-mannopyranosyl-(1→6)-α-D-mannopyranosyl-(1→6)-α-D-mannopyranosyl-(1→6)-D-mannopyranose (8)

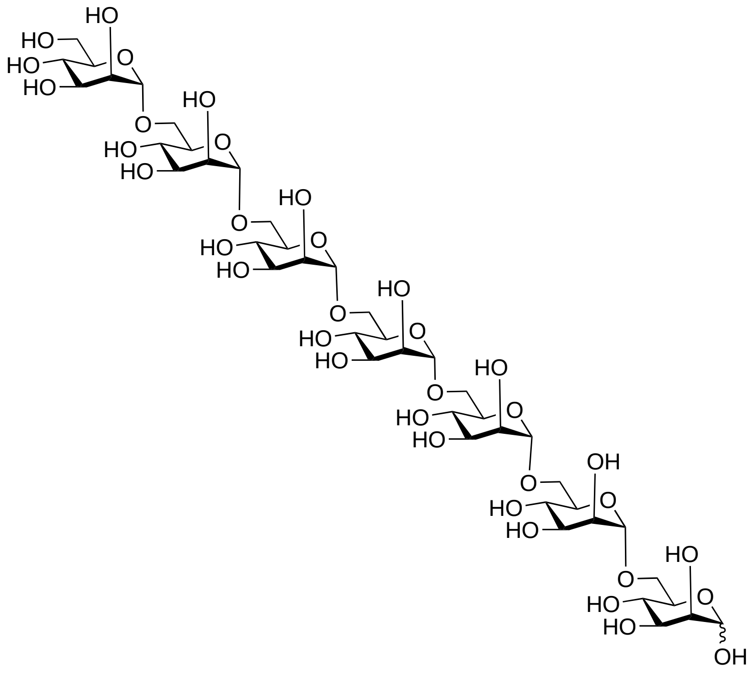

Deprotection of **7** as described in Module G and H, followed by purification using preparative HPLC (Method D) afforded compound **8** as a mixture of α and β isomers (2.8 mg, 3.3 µmol, 50% over two steps).

RP-HPLC of 8 (ELSD trace, Method A tR = 21.6 min)

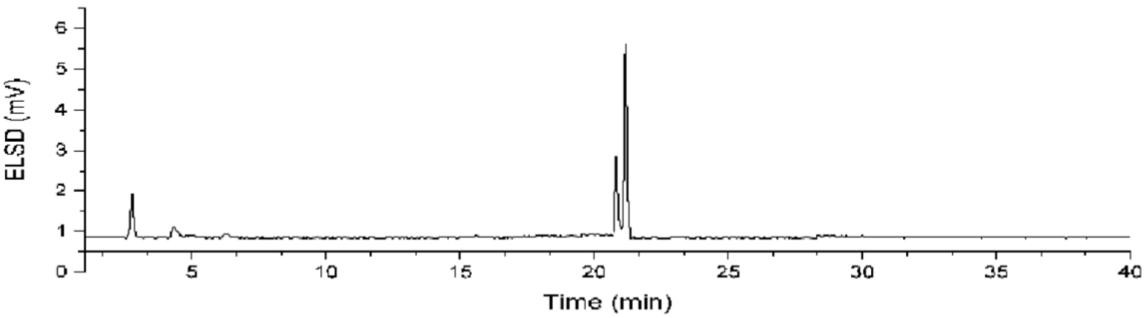

^{1}H NMR (700 MHz, D_2O) δ 5.20 – 5.14 (m, 2H), 4.94 – 4.90 (m, 14H), 4.03 – 3.99 (m, 21H), 4.00 – 3.93 (m, 28H), 3.93 – 3.84 (m, 35H), 3.85 – 3.74 (m, 27H), 3.73 (t, *J* = 10.0 Hz, 21H), 3.69 – 3.63 (m, 6H). **^{13}C NMR** (176 MHz, D_2O) δ 99.5, 99.5, 99.3, 99.2, 99.2, 94.1, 93.8, 74.1, 73.1, 72.6, 71.1, 70.8, 70.7, 70.6, 70.6, 70.5, 70.4, 69.9, 69.9, 69.8, 69.8, 66.7, 66.6, 66.6, 66.5, 66.5, 66.4, 65.7, 65.6, 65.5, 65.4, 60.9. **HRMS** $[M+Na]^+$ *m/z* Calcd for $C_{42}H_{72}O_{36}Na$, 1175.3652; found, 1175.3692.

NMR Data

Compound 1

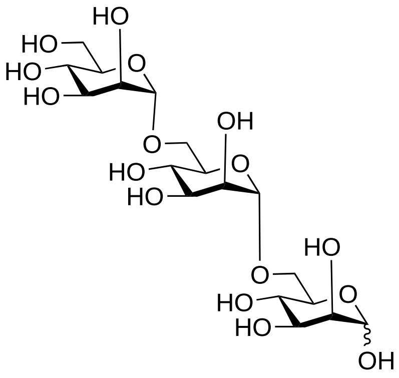

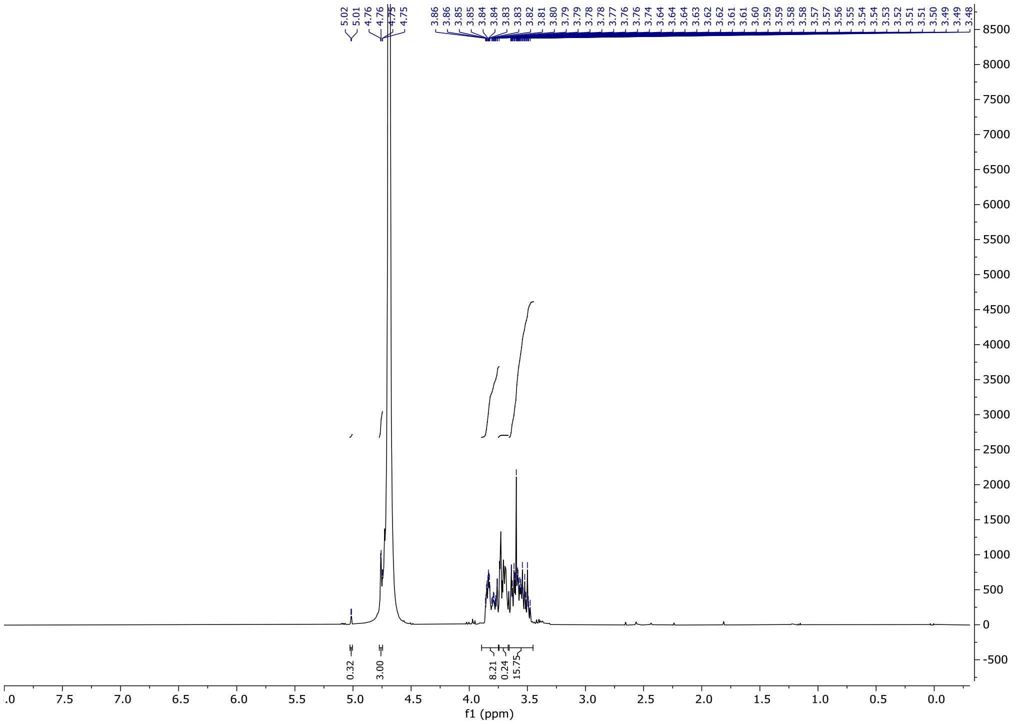

^{1}H NMR

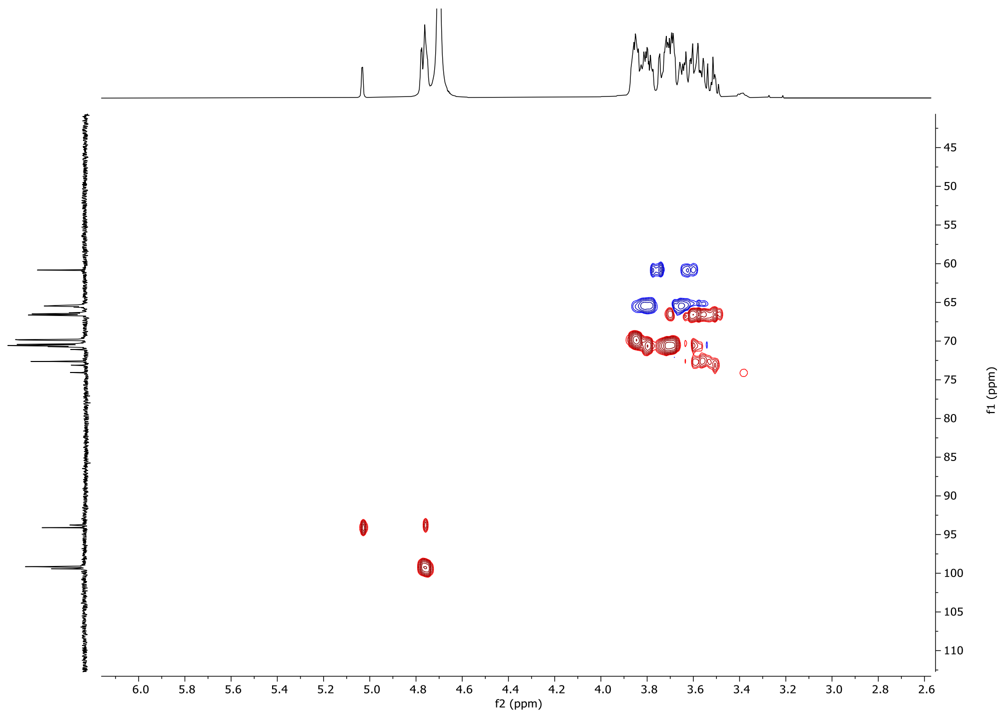

^{1}H-^{13}C HSQC

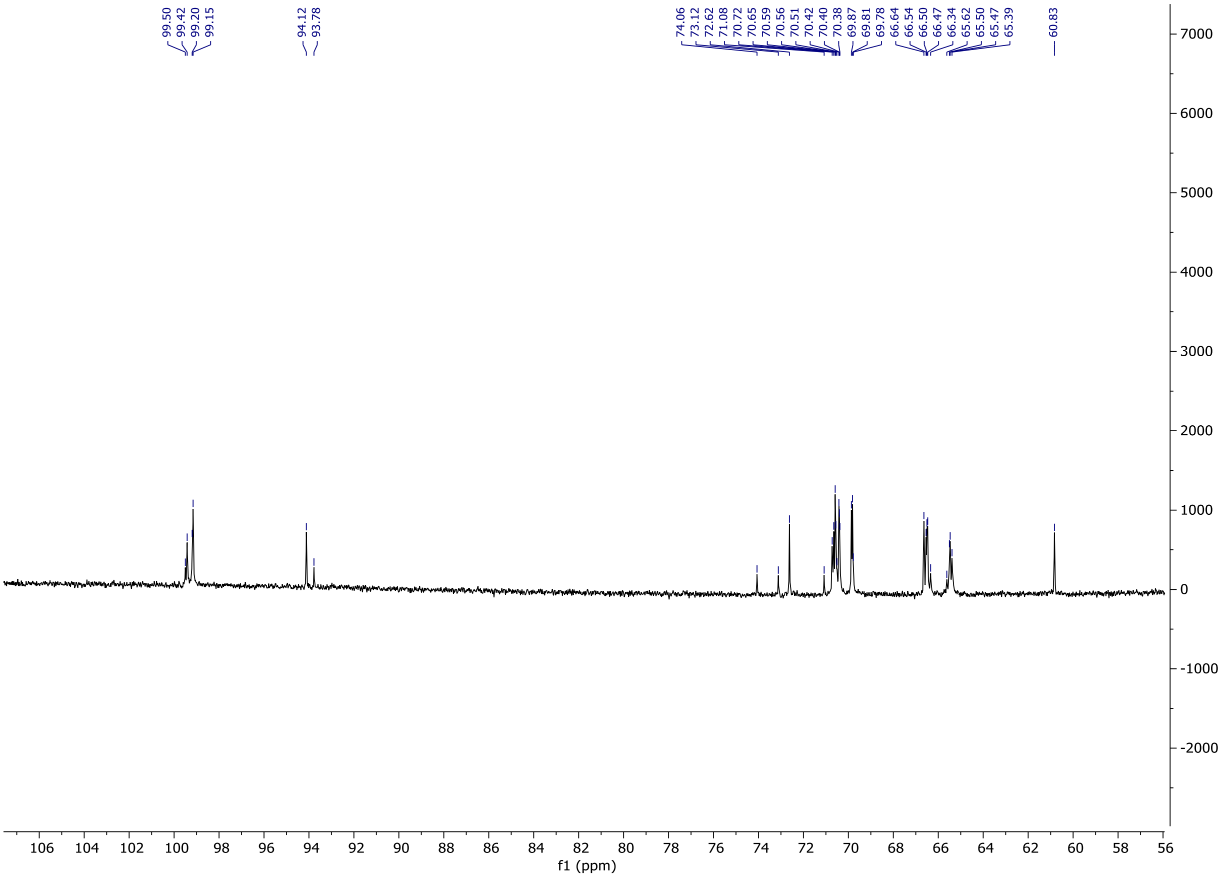
99.50
99.42
99.20
99.15
94.12
93.78
74.06
73.12
72.62
71.08
70.72
70.65
70.59
70.56
70.51
70.42
70.40
70.38
69.87
69.81
69.78
66.64
66.54
66.50
66.47
66.34
65.62
65.50
65.47
65.39
60.83
f1 (ppm)

^{13}C NMR

Compound 2

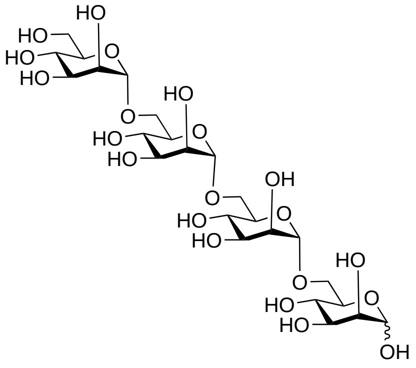

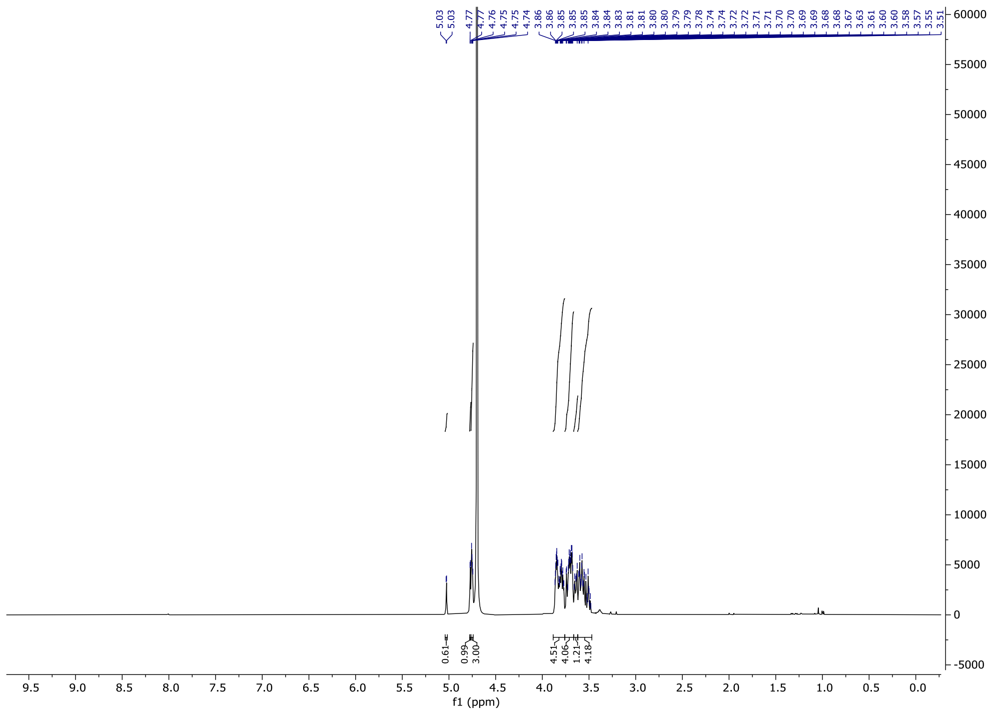

^{1}H NMR

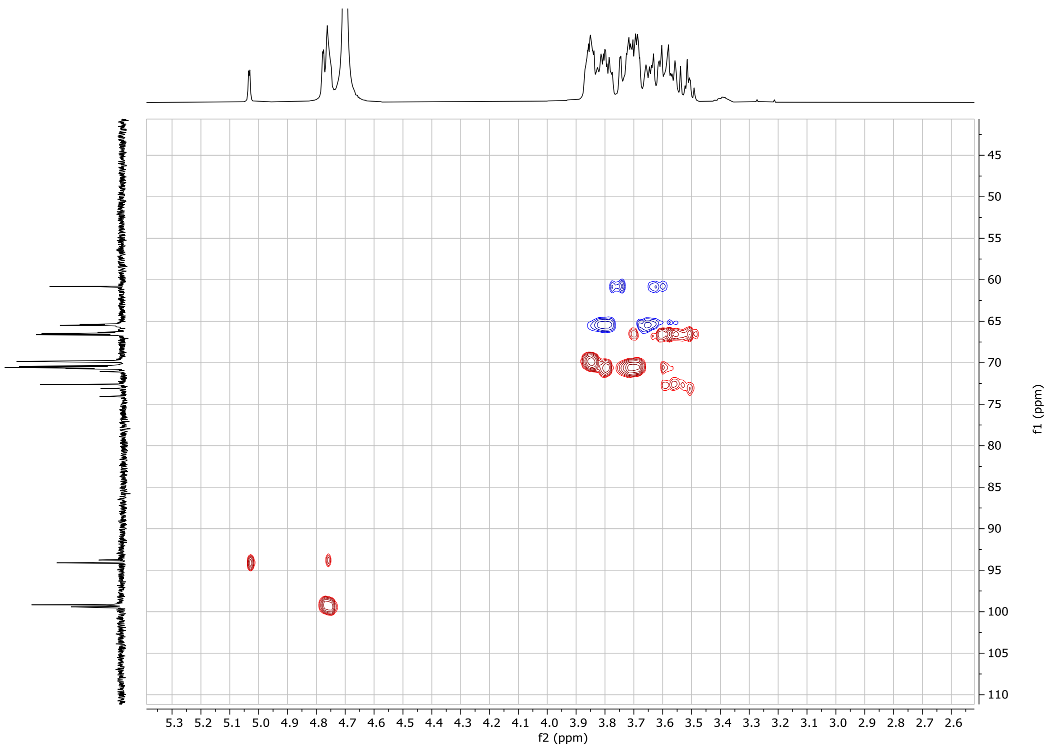

^{1}H-^{13}C HSQC

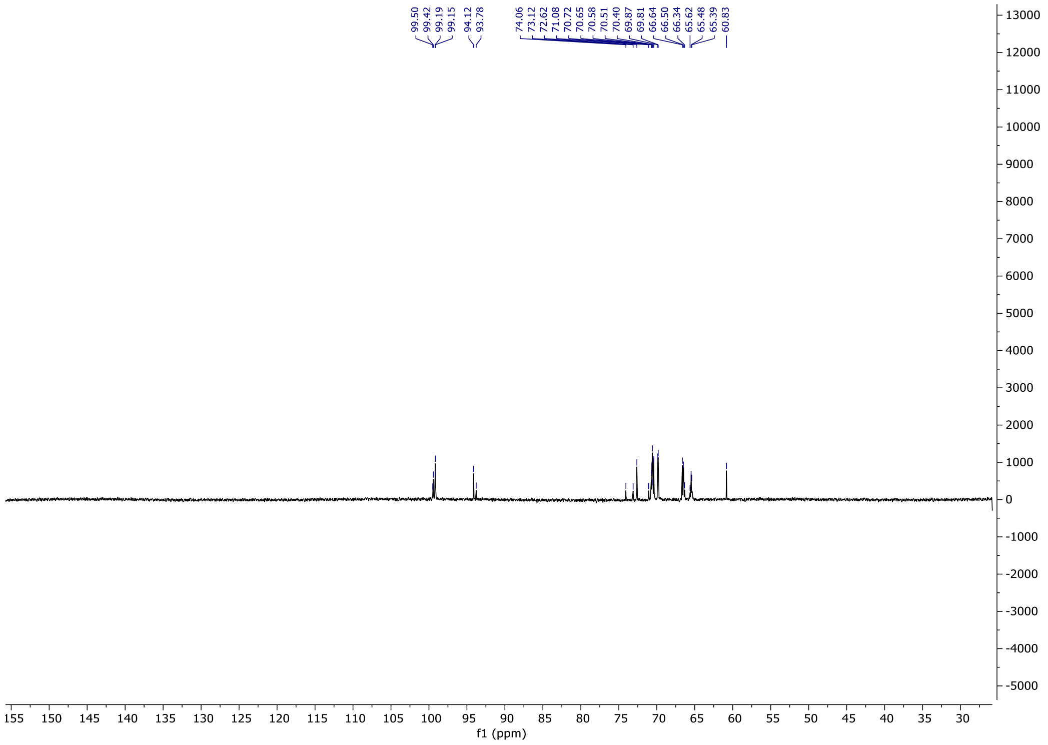

^{13}C NMR

Compound 4

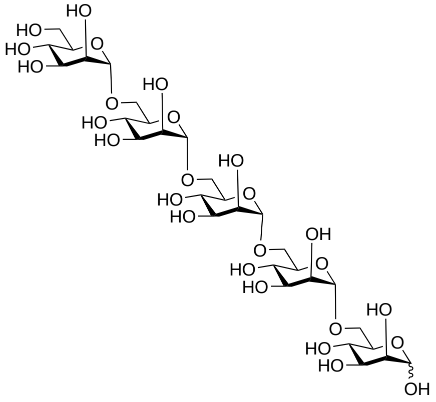

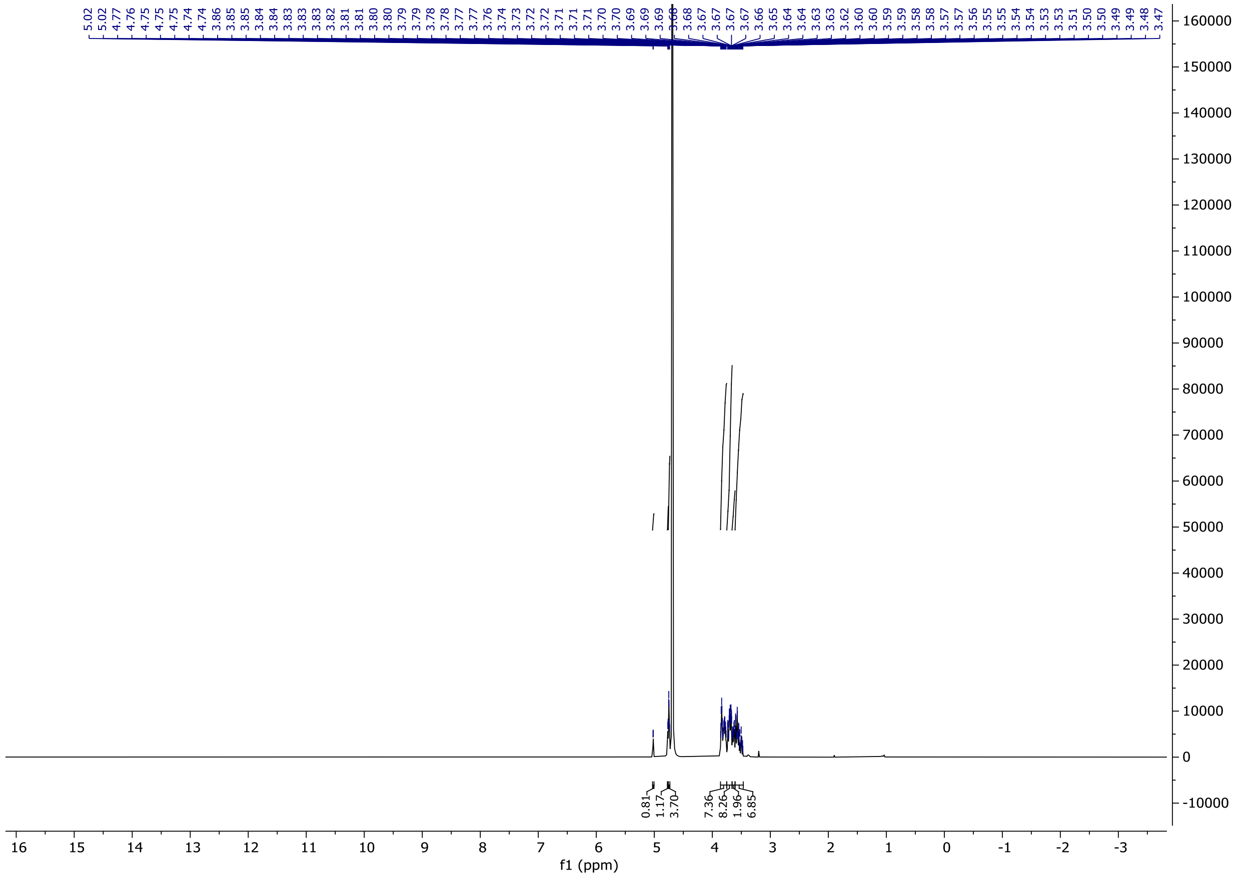

^{1}H NMR

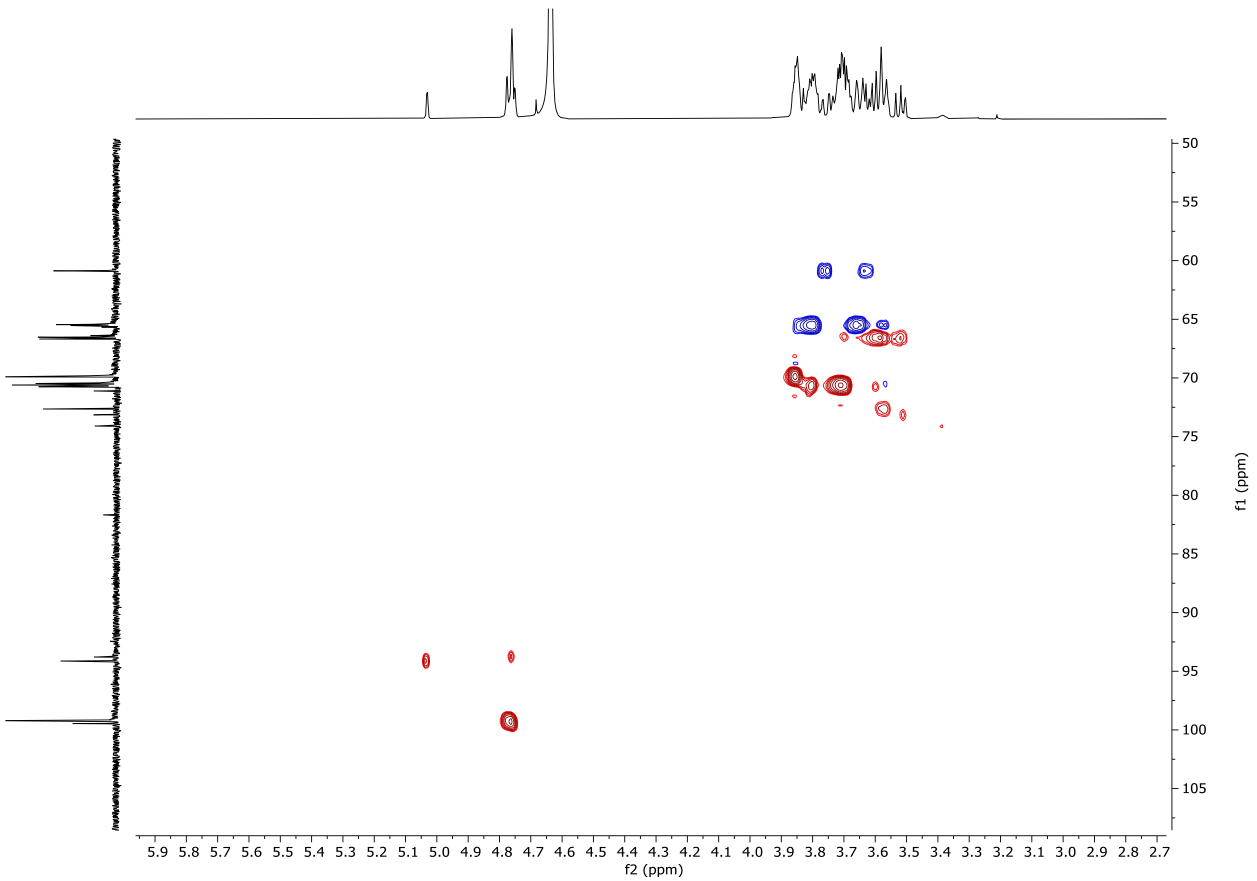

f1 (ppm)
f2 (ppm)
50
55
60
65
70
75
80
85
90
95
100
105
5.9 5.8 5.7 5.6 5.5 5.4 5.3 5.2 5.1 5.0 4.9 4.8 4.7 4.6 4.5 4.4 4.3 4.2 4.1 4.0 3.9 3.8 3.7 3.6 3.5 3.4 3.3 3.2 3.1 3.0 2.9 2.8 2.7

^{1}H-^{13}C HSQC

Compound 5

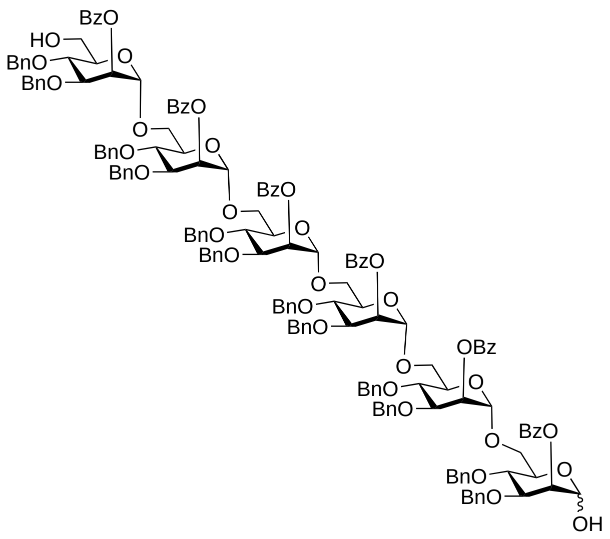

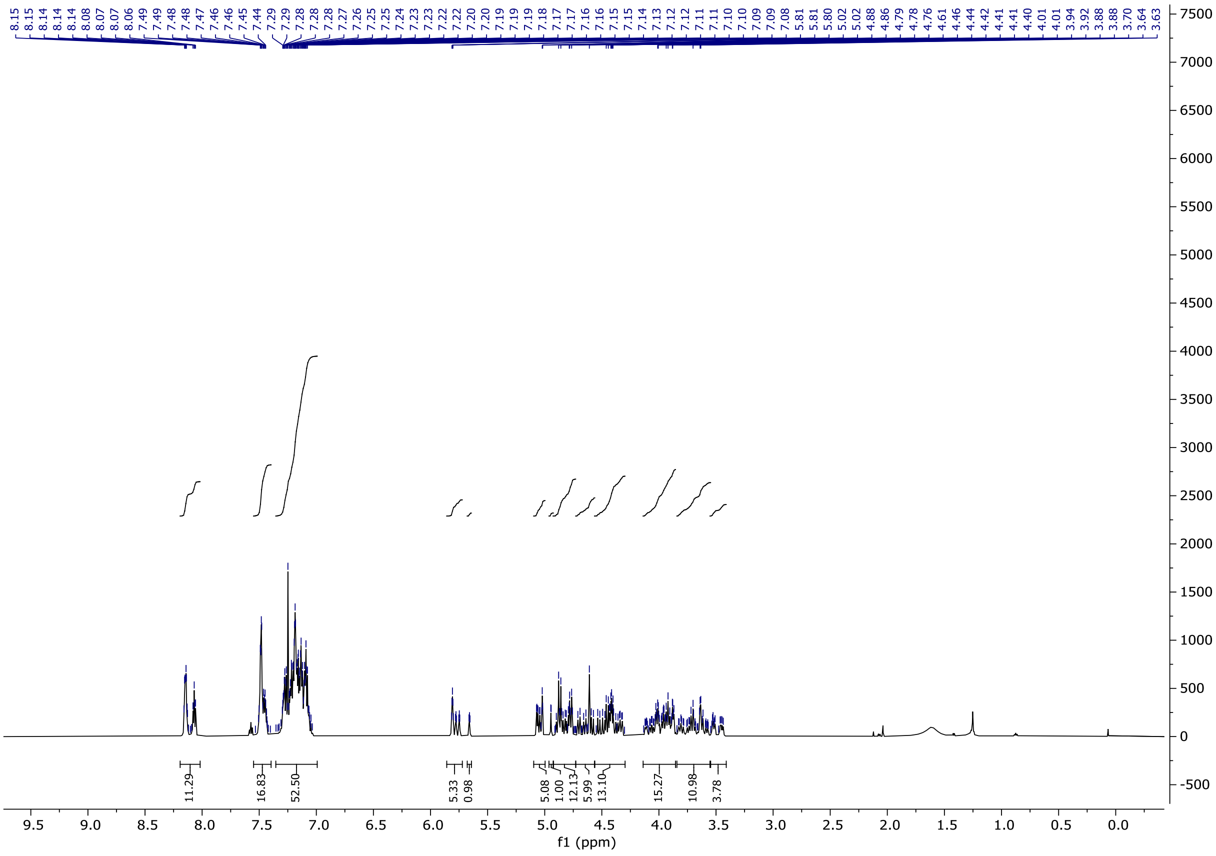

^{1}H NMR

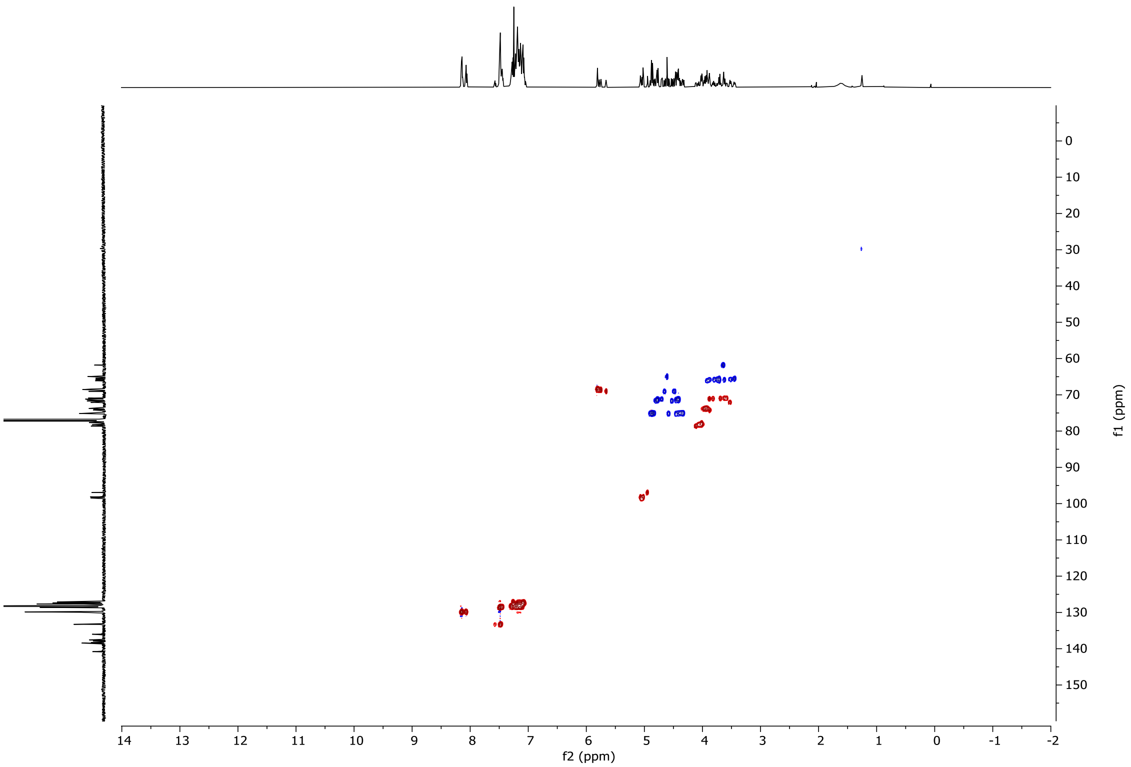

^{1}H-^{13}C HSQC

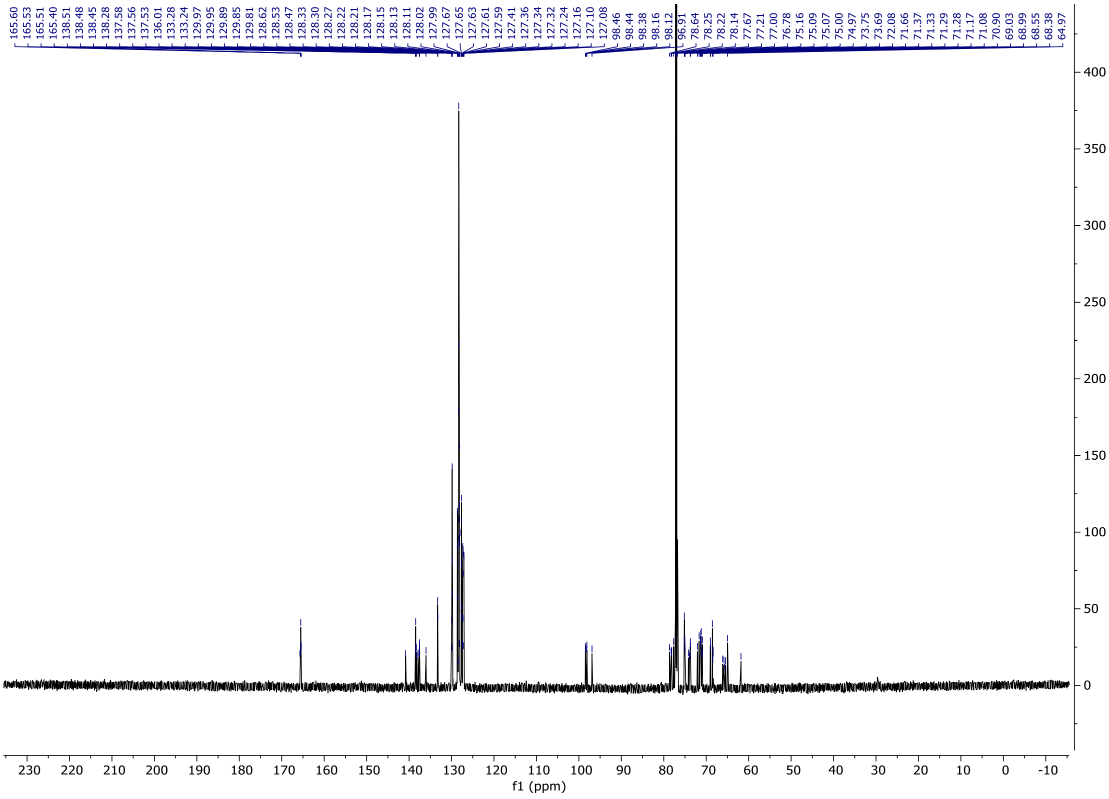

^{13}C NMR

Compound 6

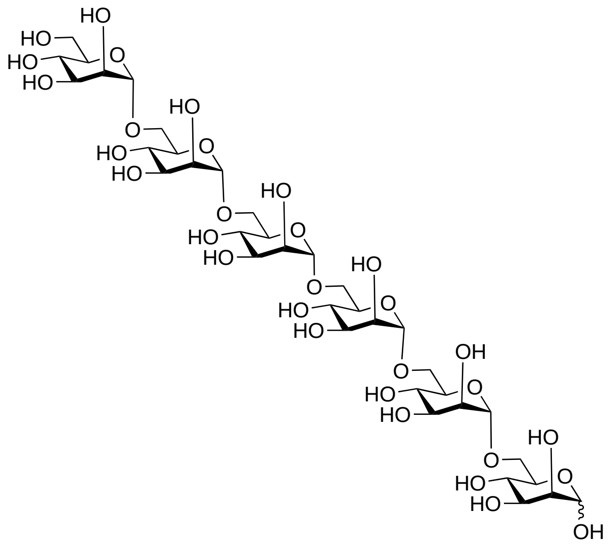

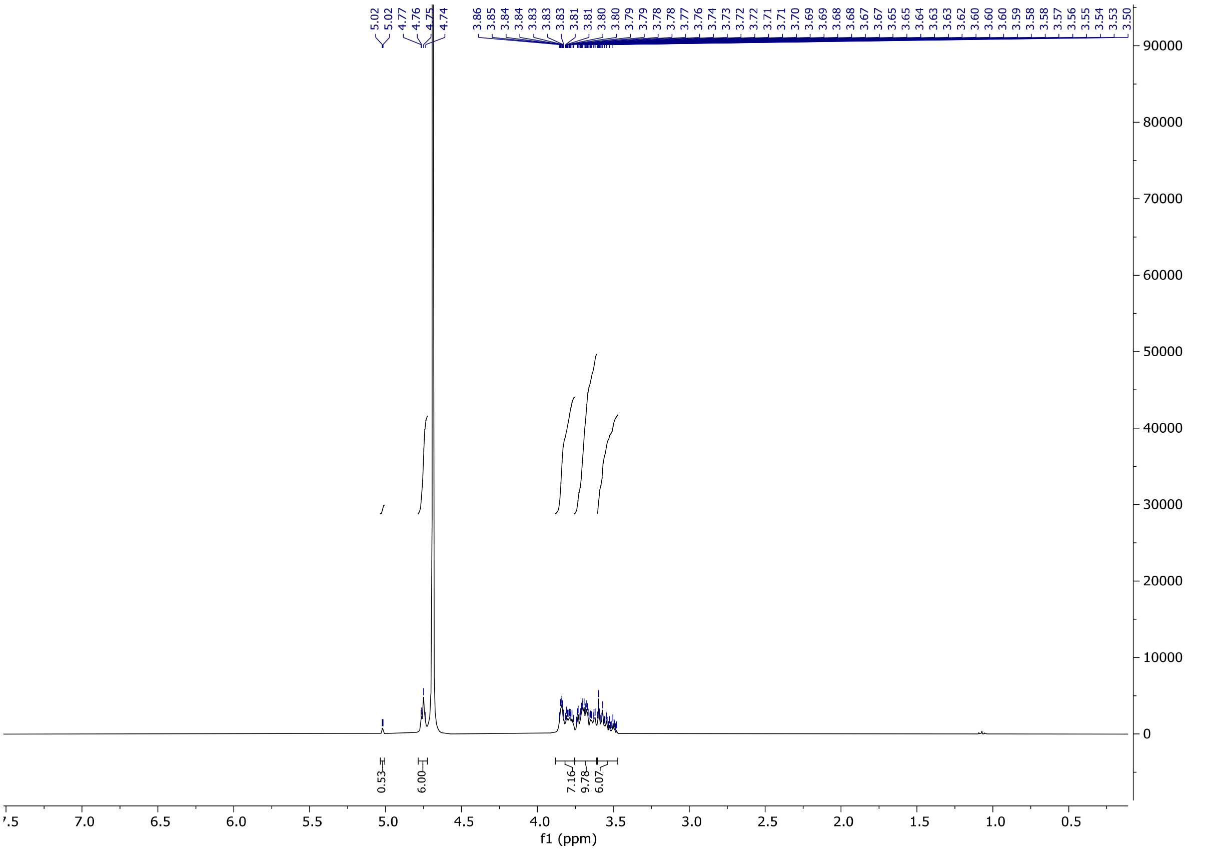

^{1}H NMR

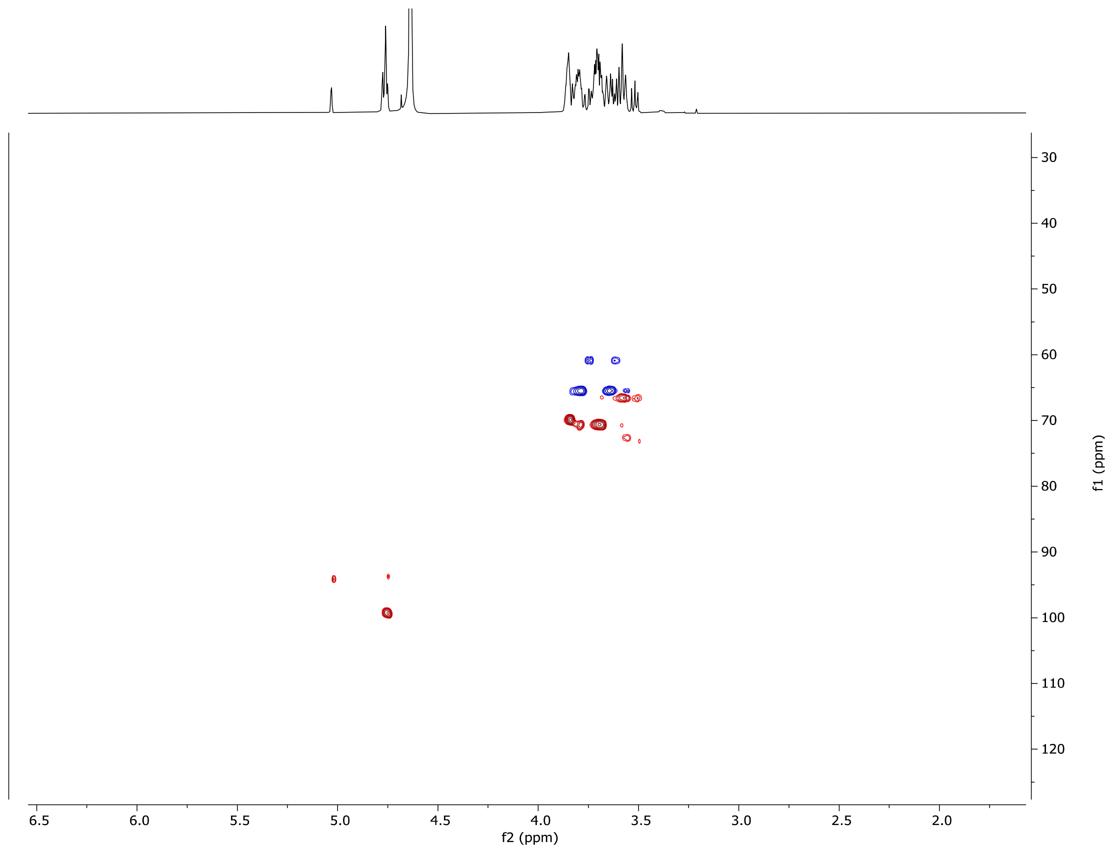

30
40
50
60
70
80
90
100
110
120
f1 (ppm)
6.5
6.0
5.5
5.0
4.5
4.0
3.5
3.0
2.5
2.0
f2 (ppm)

^{1}H-^{13}C HSQC

Compound 7

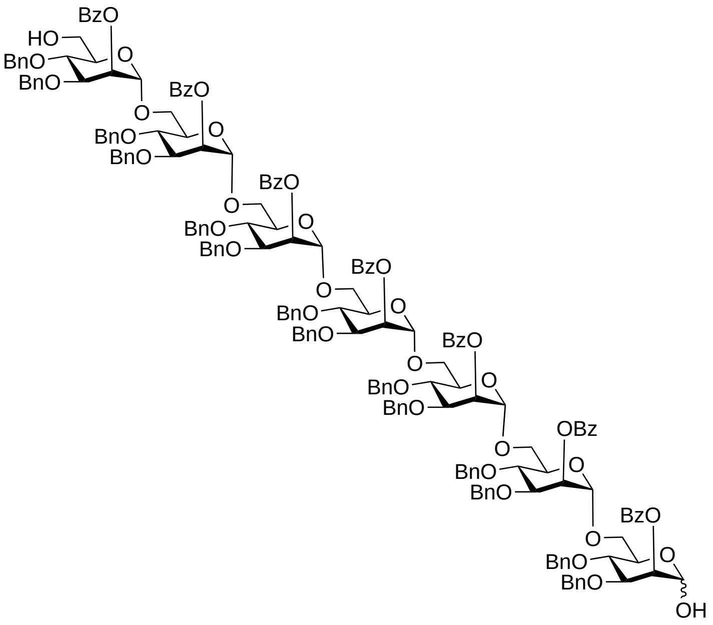

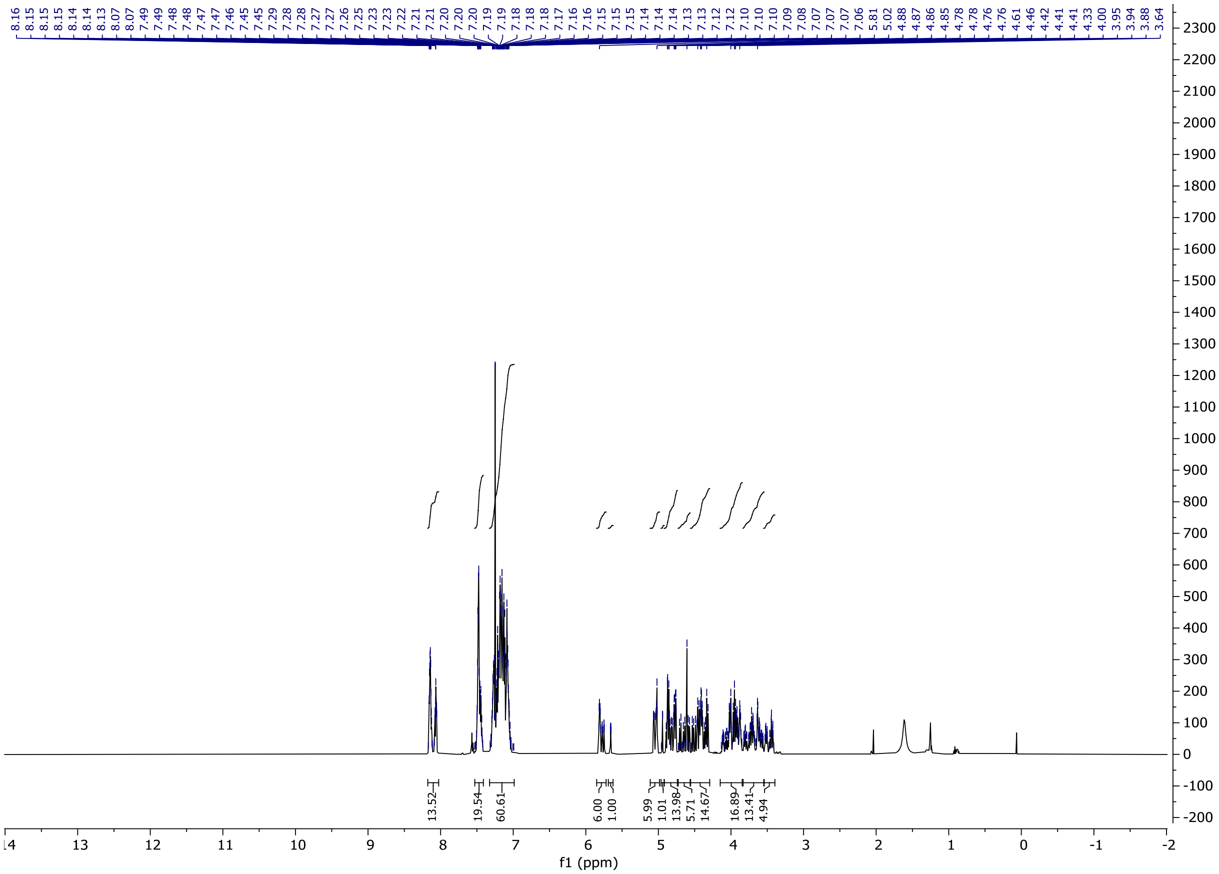

^{1}H NMR

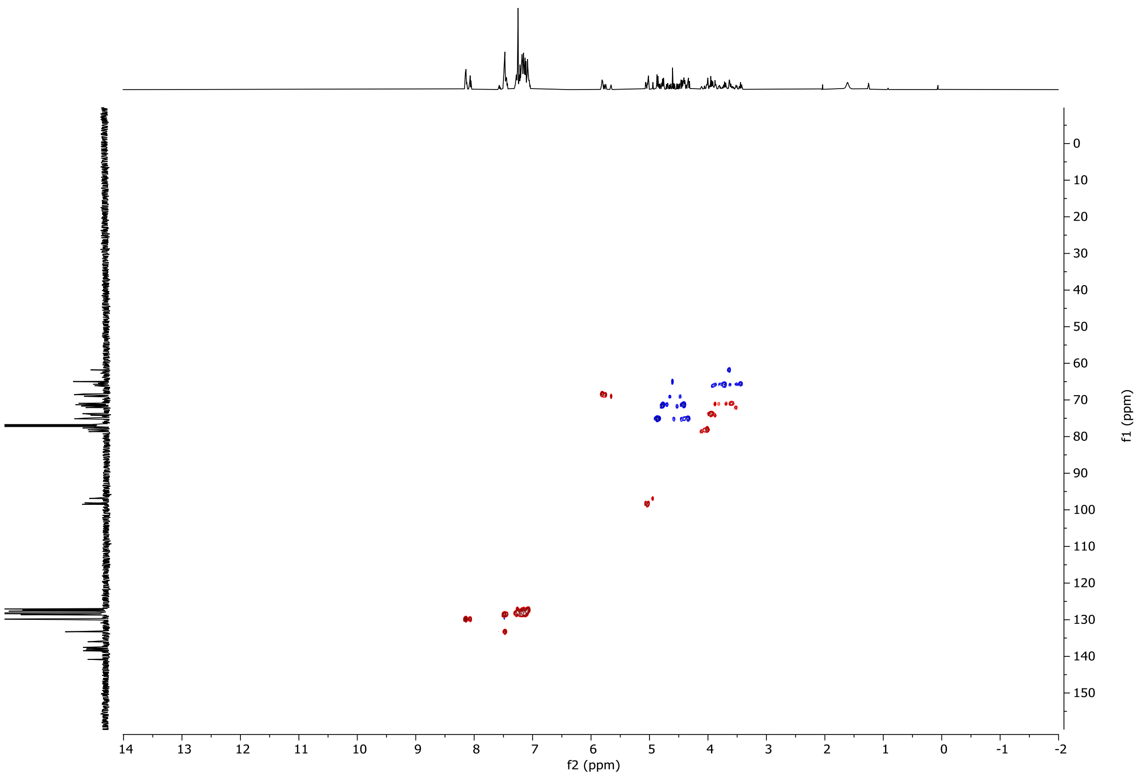

^{1}H-^{13}C HSQC

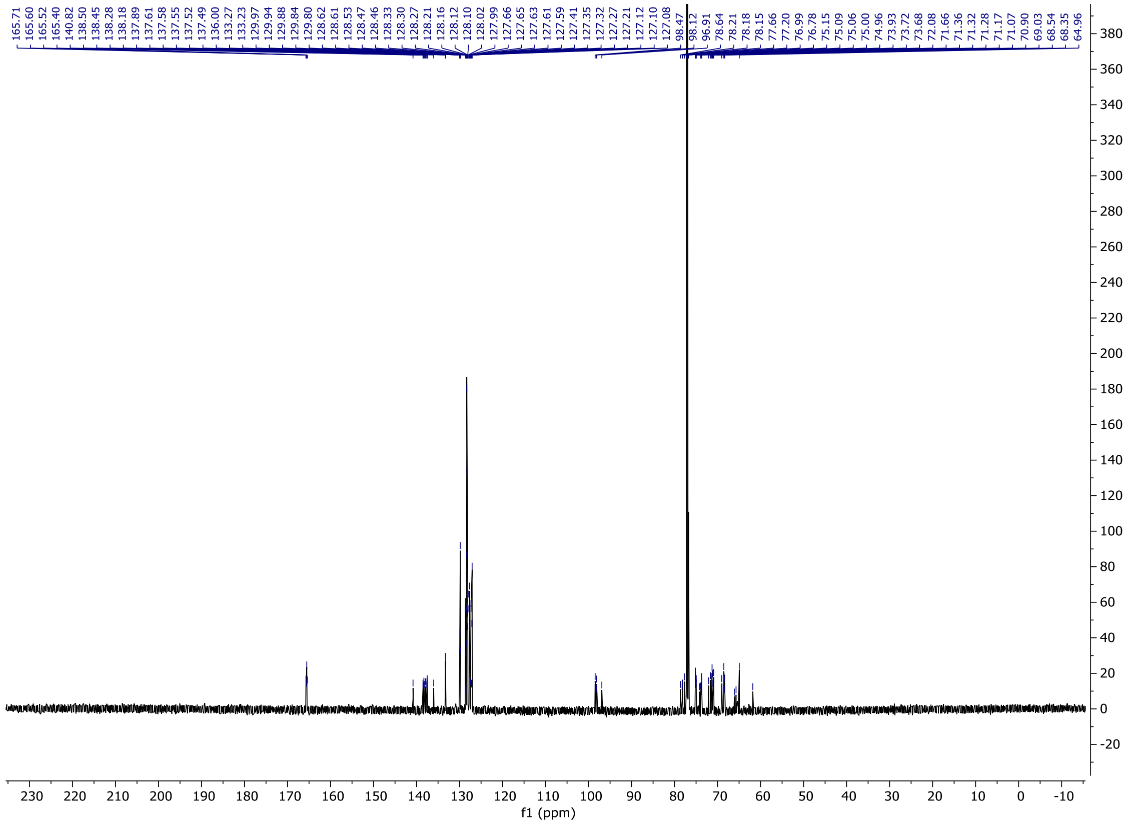
165.71
165.60
165.52
165.40
140.82
138.50
138.45
138.28
138.18
137.89
137.61
137.58
137.55
137.52
137.49
136.00
133.27
133.23
129.97
129.94
129.88
129.84
129.80
128.62
128.61
128.53
128.47
128.46
128.33
128.30
128.27
128.21
128.16
128.12
128.10
128.02
127.99
127.66
127.65
127.63
127.61
127.59
127.41
127.35
127.32
127.27
127.21
127.12
127.10
127.08
98.47
98.12
96.91
78.64
78.21
78.18
78.15
77.66
77.20
76.99
76.78
75.15
75.09
75.06
75.00
74.96
73.93
73.72
73.68
72.08
71.66
71.36
71.32
71.28
71.17
71.07
70.90
69.03
68.54
68.35
64.96
f1 (ppm)

^{13}C NMR

Compound 8

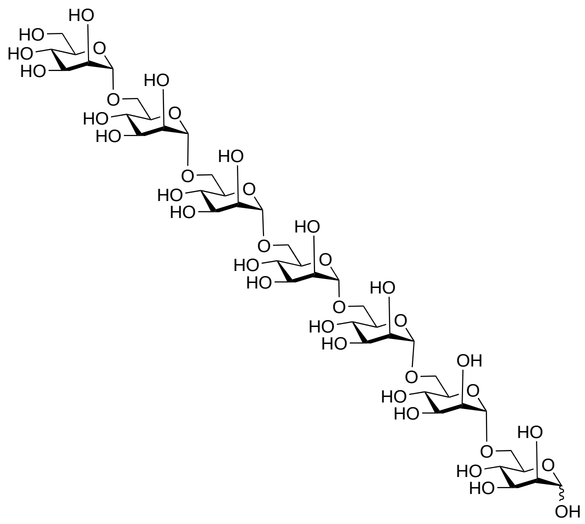

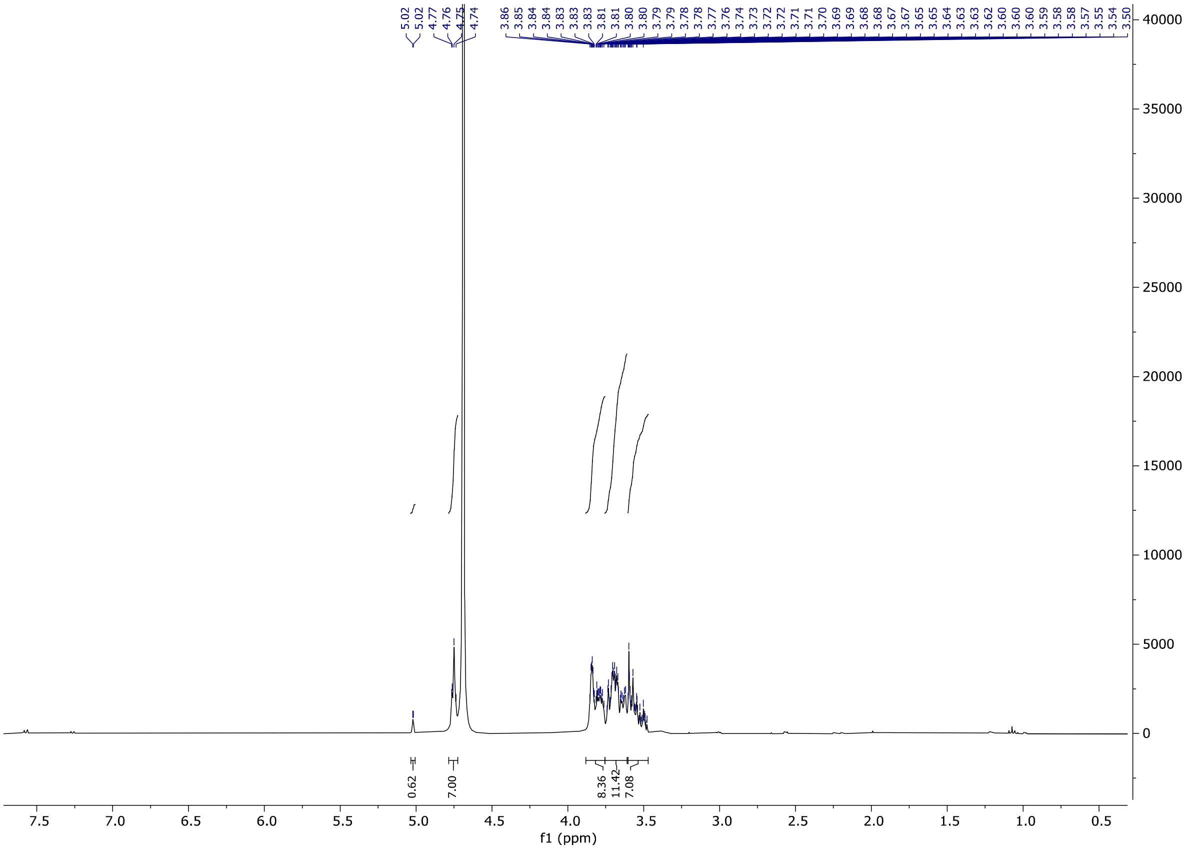

^{1}H NMR

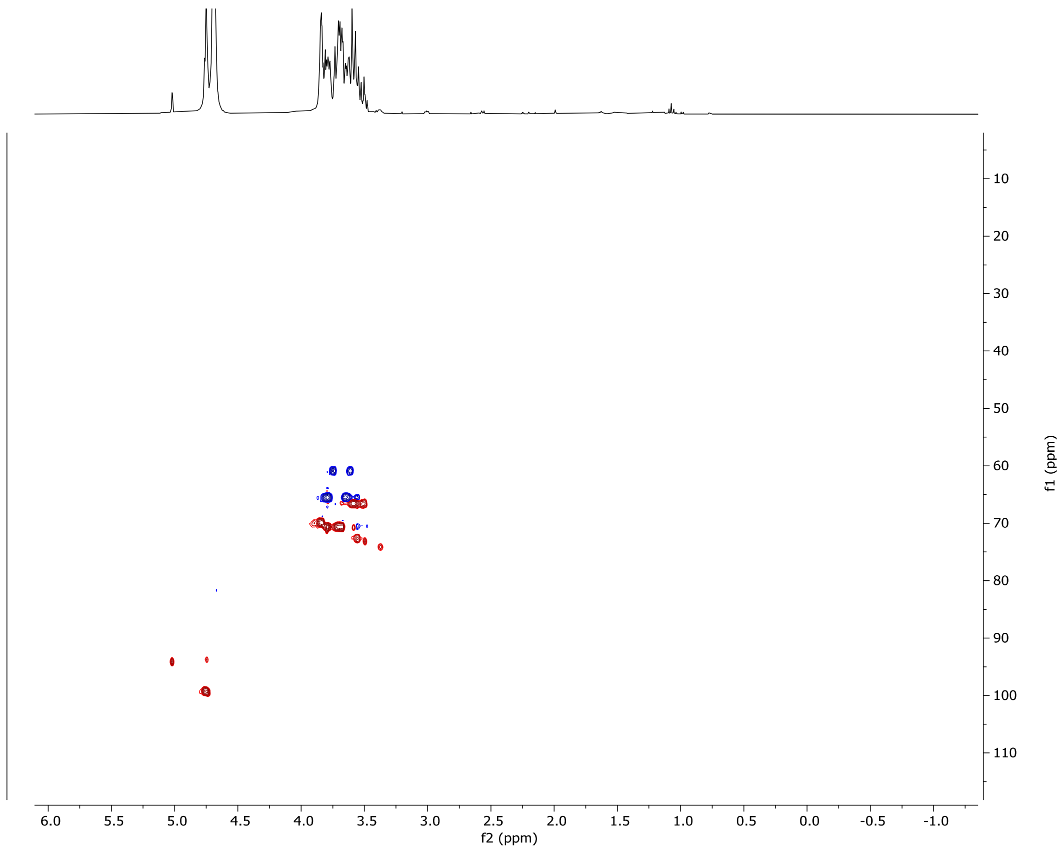
6.0
5.5
5.0
4.5
4.0
3.5
3.0
2.5
2.0
1.5
1.0
0.5
0.0
-0.5
-1.0
f2 (ppm)
10
20
30
40
50
60
70
80
90
100
110
f1 (ppm)

^{1}H-^{13}C HSQC

Supplementary methods

Determination of enzyme activity by high-performance anion-exchange chromatography- pulsed amperometric detection (HPAEC-PAD)

100 µL reactions with final concentration of 5 µg/mL protein and 0.5 mg/mL of oligosaccharide were prepared and incubated at 37 ºC, for 30 min and overnight. The enzymatic reaction was inactivated by heating at 95 ºC for 15 min. Inactivated reactions were subsequently centrifuged at 21 000 g for 30 min. The supernatant was diluted 10-fold with Milli-Q water. Aliquots of 10 µL were then injected into the HPAEC-PAD System (Dionex ICS-5000+, Thermo Fischer Scientific Inc., Waltham, MA, USA). For yeast- α-mannan and linear yeast α-mannan, the hydrolysis reactions were setup with final enzyme concentration of 10 µg/mL and 1 mg/mL of substrate. The enzymatic reaction was inactivated by heating at 95 ºC for 15 min. Inactivated reactions were subsequently centrifuged at 21000 g for 30 min. The 10 µL supernatant was then injected into the HPAEC-PAD System. Separation was performed with the eluent 1: 0.15 M NaOH (HPLC grade, VWR) and eluent 2: 0.15 M NaOH and 1 M sodium acetate (HPLC grade, Sigma-Aldrich, Munich, Germany), which were dissolved in Milli-Q water and degassed with helium for 10 min. Eluent 2 was filtered through a 0.2 µm nylon filter membrane and then degassed. Samples from each time point were analyzed separately by a linear gradient from 100% eluent 1 to 50% eluent 1 and 50% eluent 2 for 19.5 min, afterward an increase of the eluent 2 concentration to 100% over 120 s was performed. At the end, the concentration of eluent 1 was restored to 100% for 30 s. For electrochemical detection a gold working electrode and a pH reference electrode (Ag/AgCl) were used. Samples were separated at 25 °C on a 2 x 250 mm CarboPac PA100 analytical column (Dionex, Sunnyvale, CA, USA) coupled with a 2 x 50 mm CarboPac PA100 guard column (Dionex). Corresponding synthetic linear α-1,6-mannooligosaccharides were used as reference at

concentrations of 0.3-10 µg/mL. The entire separation was conducted at an isocratic flow rate of 0.25 mL·min^{-1}. The optimum pH for α-mannanase activity was confirmed by a p-hydroxybenzoic acid hydrazide (PAHBAH) colorimetric assay (3).

ShGH76 mutant dot-blots

Nitrocellulose blotting membrane with pore size of 0.45µm (Cytiva, Freiburg, Germany) was cut into 2.5 by 2.5 cm pieces and sectioned into twelve equal squares by pencil. The squares were then spotted with either yeast α-mannan (unmodified) or linear yeast α-mannan, galactomannan (Megazyme Ltd., Bray County. Wicklow, Ireland), and starch (Sigma-Aldrich) by pipetting duplicates of 1 µL of 4 mg/mL and 15 mg/mL solutions on distinct squares. After drying for 10 min under a lamp, the paper was put in a 6-well plate. Blocking was done by adding 5% w/v milk phosphate buffered saline (MPBS, 2.5 mL per well) and subsequent incubation on a vertical shaker for 2 h at ~50 RPM (room temperature). After discarding the MPBS, wells were washed four times with 1x PBS (2 mL per well, gently shaking). The last washing step was performed for 10 min on a vertical shaker at ~50 RPM (room temperature). In the following steps, all proteins and antibodies were diluted with 5% w/v MPBS for their final concentrations. The proteins ShGH76^{Ala} and ShGH76^{Asn} (20 µg/mL) and concanavalin A conjugated with peroxidase (20 µg/mL, protein is known to bind to terminal mannosyl residue as a control) (Sigma-Aldrich) and LM21 antibody (1:10 dilution, specific antibody raised in rat for galactomannan, Biosupplies, Bundoora, Australia) were added to the wells (2 mL per well) and incubated overnight at 4 ºC. As control we used a protein buffer of 20 mM HEPES, pH 7.5, with 250 mM NaCl diluted with 5% MPBS. After discarding of liquids, wells were washed four times with 2 mL PBS (gently shaking). The last washing step was performed for 10 min on a vertical shaker at ~50 RPM (room

temperature). The washing solution was discarded and anti-His-HRP-conjugated antibody (1:1 500 dilution in protein wells, Sigma-Aldrich), anti-rat-HRP-conjugated antibody (1:5 000 dilution, in LM21-Ab well, Sigma-Aldrich) and 5% MPBS (in buffer control well) was added. The plate was then incubated for 2 h at RT at ~50 RPM. Again, liquids were discarded and wells were washed four times with 2 mL PBS. Finally, 2 mL tetramethylbenzidine (Sigma-Aldrich) were added to each well, and afterwards wells were covered for light protection. Within 2-5 min, blue dots showed up, which were then washed with sterile distilled water. The nitrocellulose papers were dried on filter paper and photographed for analysis. In the assay with linear yeast α-mannan, two blots with ShGH76^{WT} pre-treatment were included to determine epitope deletion. Galactomannan (linear ß-1,4-mannan backbone) and starch (branched α-1,4/6 backbone) were used as controls. Yeast α-mannan was able to bind to nitrocellulose membranes where it remained throughout the assays. This was confirmed by addition of concanavalin A as control protein, which is known to bind D-mannose residues (4).

Initial rates determination and Kinetics of linear yeast α-mannan

The initial rates on linear yeast α-mannan by the endo-mannanase *Sh*GH76 were determined with 2.5 mg/mL substrate in 20 mM Tris-HCl pH 8.0 at 37 °C. The hydrolytic activity was determined after 30 min in a 100 µL reaction volume. Released reducing sugars were measured with the 4-hydroxybenzoic acid hydrazide (PAHBAH) method described by Lever (3), with mannose as standard. All hydrolysis assays were carried out at 9 different endo-mannanase concentrations ranging from (0.032nM to 20 µM). Initial rates were calculated in the initial linear range of the hydrolysis.

The kinetic constants for linear yeast α-mannan hydrolysis were determined by assaying the initial endo-mannanase rates at different substrate concentrations (2 to 0.1 mg/mL) using the PAHBAH assay as described above. The enzyme concentration used for linear yeast α-mannan hydrolysis was 50 nM. The initial hydrolysis rate, Vi, was plotted as a function of the substrate concentration, [S]. Non-linear regression using the Michaelis-Menten equation was used to determine the values for k_{cat}, K_M and k_{cat}/K_M.

Supplementary Results

Additional structural features of ShGH76

During crystallization, some of the crystallization buffer components were co-crystallized with $ShGH76^{Ala}$ and resulted in interesting crystallization interactions. The $ShGH76^{Ala}$ structure has additional 2-(N-morpholino)ethanesulfonic acid (MES) and glycerol ligand molecules, which are components of the crystallization conditions (Fig. 6B). MES is in close proximity to the Asn336, Gln337 and Glu340 side chains. $ShGH76^{Asn}$ protein carries three calcium ions on its surface (Fig. 6C), that mediate crystal-contacts with neighboring $ShGH76^{Asn}$ symmetry-mates.

To accommodate the large and branched yeast α-mannan, the ShGH76 barrel motif appears less decorated, with face of the barrel covered with major loops connecting short strands, linking consecutive helices (Fig. S6B). This loop-decorated region forming natural cavity at the center of the barrel structure forms a large negatively charged cleft (Fig. 7B) consistent with a binding site adapted for complex carbohydrate molecules (5). This is additionally supported by observation of the high degree of sequence conservation within this extended binding pocket (Fig. S6C). However, the structural comparison of $ShGH76^{WT}$ with human gut bacterial homologs (BT2949-GH76, BT3792-GH76, BT3782-GH76) revealed variations in loop conformations and in loops

connecting helices α2-α3, α3-α4, α10-α11 as well as the extended β-strands connecting helices α9-α10 (Fig. S7A) (6, 7). The recently resolved structure of BtGH76-MD40 from bovine-adapted *B. thetaiotaomicron* MD40 has an additional N-terminal domain, consisting entirely of β-strands (Fig. S7B) (8). Superimposition of ShGH76^{WT} with *Bacillus circulans* BcGH76, *Listeria innocua* LiGH76 and BtGH76-MD40 displayed similar extended β-strands between helices α9-α10 as in human gut bacterial GH76 homologs, extended loops between helices α3-α4 and large loop conformational changes in the loops connecting helices α7-α8 and helices α1-α2 (Fig. S7B) (7, 9).

All gut bacterial GH76 homologs from *B. thetaiotaomicron* possess signal peptides and are either surface or periplasmic enzymes (6-8) (Table S8). Based on LipoP (10) and SignalP (11) predictions, ShGH76 has similar signal peptide at N-terminal (Table S8) that was removed during cloning for this study.

Auxiliary catalytic residues assist in hydrolysis

Glycosidase active sites typically involve a carboxylate pair as core catalytic residues. These are assisted by coordinating hydrogen-bond donors and aromatic side chains that position the substrate (12, 13). Within the ShGH76 active pocket, three conserved solvent-exposed carboxylates, Asp136, Asp137 and Asp239, are in close proximity to four conserved aromatic residues, Trp90, Trp140, Tyr258 and Phe313 (Fig. 8A and 8B). Further conserved residues contribute to the active site, namely Phe134, Trp182, Phe256, Asn259 and Asp309, (6-8, 14) (PDB IDs: 5AGD, 6U4Z, 4MU9, 4C1S, 4V1S and 3K7X) (Figs. 8A and S4). Comparison of substrate-bound mutants with the apo-form structure of ShGH76^{WT} revealed that Asp239 and Asp309 play a role in substrate binding (Figs. 8A, S5A and S5B), as their carboxylate groups point towards the glycosidic bond at a distance of less than 3 Å (Fig. S6B).

Active sites of ShGH76 mutants revealed involvement of aromatic, charged and hydrophobic residues in the catalytic pocket (Figs. S5A and S5B). Comparison of the two previously solved homologs from *B. circulans* TN-31 having α-1,6-linked-manno-oligosaccharides i.e mannobiose (PDB: 4BOJ) (unpublished) and mannopentaose (PDB:5AGD) (14) in their active sites showed that ten out of twelve amino acids are conserved and do not possess significant conformational variations. The remaining two amino acids Phe256 (Thr241 in 4BOJ and 5AGD) and Phe313 (Gly298 in 4BOJ and 5AGD) in ShGH76 affect binding of the non-reducing terminal mannose residues in Man3 and Man4. Phe256 and Phe313 sharply bent Man3 and Man4 conformations (Figs. S7C and S7D).

The supplementary Table S1 is available as separate Microsoft Excel file. Legend is given below:

Supplementary Table S1: Overview of metagenomes used in this study.

Supplementary Table S2: *Salegentibacter* strains showing presence and absence of GH76 gene.

Strain	Assembly [Mbp]	GH76	Isolation source	Reference
S. agarivorans DSM 23515	4.30	WP_093304467.1, SFF83034.1	Sponge *Artemisina sp*	(15)
S. echinorum DSM 24579	3.97	Not present	Sea urchin *Hemicentrotus pulcherrimus*	(16)
S. flavus DSM 17794	3.69	Not present	Sediment sample collected in Chazhma Bay (Sea of Japan)	(17)
S. holothuriorum DSM 23405	3.59	Not present	Edible holothurian *Apostichopus japonicus*	(18)
S. mishustinae DSM 23404	3.82	Not present	Sea urchin *Strongylocentrotus intermedius*	(19)
S. mishustinae KCTC 1226	3.78	Not present	Not available	(19)
S. mishustinae DSM 23404	3.77	Not present	Sea urchin *Strongylocentrotus intermedius*	(19)
S. mishustinae KCTC 12263	3.78	Not present	Not available	(19)
S. salarius KCTC 12974	3.32	Not present	Not available	(20)
S. salarius DSM 23401	3.33	Not present	Marine solar saltern	(20)
S. salarius KCTC 12974	3.32	Not present	Not available	(20)
S. salegens ACAM 48	4.01	Not present	Antarctica: Organic Lake	(21)
S. salegens DSM 5424	3.87	Not present	Not available	(21)
S. salinarum KCTC 12975	4.31	PKD16392.1, PKD16394.1, PKD20402.1	Not available	(22)
S. salinarum DSM 23400	4.31	SKB63511.1 (WP_079712844.1), SKB63521.1 (WP_079712846.1), SKB85168.1 (incomplete)	Marine solar saltern	(22)
S. sediminis K5023	3.26	Not present	Marine sediment, China: the coast of Weihai	(23)
S. sp. 24	4.15	TDN95057.1 (WP_133546714.1)	Susquehanna River, Pennsylvania, USA	Unpublished
S. sp. BLCTC	3.65	Not present	Seawater, China	Unpublished

S. sp. F60176	3.59	Not present	Sediment, China	Unpublished
S. sp. F63223	3.89	Not present	Sediment, China	Unpublished
S. sp. Hel_1_6	4.21	WP_037317868.1	Surface seawater, Helgoland, Germany	Unpublished
S. sp. R32	4.08	Not present	Marine sediment	Unpublished
S. sp. T436	4.09	APS37752.1, WP_075325016.1	Arctic ocean, Fram Strait	Unpublished
S. sp. UBA1130	4.05	Not present	Hydrothermal vent metagenome	Unpublished

Supplementary Table S3: Protein sequences of GH76s from *Salegentibacter* strains and GH76s from CAZyme database.

Sr. no.	Protein detail	Protein sequence
1	>WP_037317868.1 hypothetical protein [*Salegentibacter sp.* Hel_1_6]	MLFLISCSADDDGIDDVQEEEEEQPVEPGEEEDDEVADWGEVAENLQEQTYNIYLTSNGTFRQDNEGNENFNYWWNA HMLDVLIDGYERTGDESYLPKMKSLLEGIEVRNGNKYENVFIDDMEWLGIACLRTYKLTNDQQYKEVADLLWEETKQ GWSDVHGGGIAWKTDTPNSKNACSNGPAAIFALYLYEIDQDEEDLEWAKKIYHWLKDTLVDPESGLVWDNIDYHDG EAIINRDWIFTYNVGTYIGAANLLHQATGEGMYLDDAIKSASSVVAPGELTTGGVLKNEGQGDGGLFKGILVRYFTQL ALNPDLPDGKRNEFEEFVLFNAETLYHNGLTSAGLAGPNWNDEPSGRVDLSTQLSGVMLMEAKALLE
2	>WP_133546714.1 glycosyl hydrolase family 76 [*Salegentibacter sp.* 24]	MKIVRLGVLFLTLFFLNSCSTDGENLPEVEEKENETPSEPDEDEEDFEEEVSDWGEVSQKFQQITYDTYLSSAGTFGQDN LGNQNFNYWWNAHMLDVLVDGYKRSGNDAYLPRMKALLEGIKTRNGNRYKNDFIDDMEWLGIASLRAYEETGDES YKEVADLLWVEVKKAWSDVHGGGIAWKTDTPNSKNACSNGPAAIFALYLYQIDQDPEDLQWAKDIYHWLKNTLVDP DTGLVWDNINFQDGQAVINRDWIFTYNVGTYIGAANLLHQTTGNSVYLQDAMKSANSVISSGELTTGGILKNEGQGD GGLFKGILVRYLTQLALNPELAVEKQEQYKEFIQFNAETLYSNGRSDQGLFGPNWSDQPSGRVDLSTQLSGVMLMEAN ALLNGS
3	>WP_075325016.1 hypothetical protein [*Salegentibacter sp.* T436]	MKFTSSNLSLAGLLKPFAPIFILITLGAHSLVFGKEKPTSAPSKLVTLENNNKWSHLANSLQESTYKNYLGENGVFTQDN KEQNKFHYWWNAHMVDILIDGYLRTKDESYLPKIKDLVRGIKNSNQDNFQIYFNDDMEWLGIACVRAYRATGDEEY KQVAEYLWEEVKKGWTDVHGGGIMWRTDTPEEKNACSNGPGALLALNLYAINSNEDELEWAKKIFEWQKNTLVDPL TGLVWDNISYQDEEAVINKDLVLSYNQGTYIGAATQLFNHTGNKEYLEEAWKTTNSLMRSPKLTFEGILRSEGQGDGG LFKGILVRNLTLLAENPALANQRKELLLDFLAFNANTLRTFGLDKKEMVVSPNWAQKPKNQTDLSTQLSGVMMMEM AARLNLKQENVKRYCQQNEAIYKKPHFTTLSKEEIVIVVLGSSTAEGIGPTKKENAWVPRFIDYLQESDTNFKVINLAK GGFTTADLLPDGHPDKNISKALSYNPEAIIINLPSNDAAEGRNTPEQLENYQKIFNSIKNEIPVWVTTPQPRNFEKAKVNI QKDMVDATYAYFDKEFIIDLWTCFATEKNLIKKDYDSGDGIHLNDEAHKLIFERVVLSLSNFLKQG
4	>APS37750.1 hypothetical protein [*Salegentibacter sp.* T436]	MRHKNYVIMRILVFGIFFLSSLLFLISCSTDNDNLEDIQEEEEEEQPVDPGDEEEDEVDNWAEVAENLQEETYSVYLASN GTFRQDNEGNENFNYWWNAHMLDVLIDGYQRTGDESYLPKMKSLLEGIKIKNGNKYENVFIDDMEWLGIACLRAYK LTNDEQYKEVADLLWEEVKKGWSDVHGGGIAWKTDTPNSKNACSNGPAAIFALYLYEIDQDQEDLEWAKKIYQWLN DTLVDPESGLVWDNIDYQDGEAVINRDWIFTYNVGTYIGAANLLHQATGEEMYLEDAIKSASSVVGPGQITTGGVLKN EGQGDGGLFKGILVRYFTQLTLNPNTPDDKQEEFKEFIQFNAETLYHNGLTTAGLAGPNWNDEPSGRVDLSTQLSGVM LMEAKALLDRQ
5	>WP_079712846.1 hypothetical protein [*Salegentibacter salinarum*]	MKSISSNLSLAGLLKPYLPIFILFTLGFHSLVFGKEKRSSAPSLVTVENNNKWSQLANSLQESTYKNYLGEKGVFIQDNK GQDKFHYWWNAHMVDILIDGYLRTKDKSYLPKIKDLVRGIKISNQDNFQIFFNDDMEWLGIACVRAYQATGDEEYKQ VAEYLWEEIKKGWTEVHGGGIIWRTDTPEEKNACSNGPGALLALNLYNINNTQAELEWAKKIFEWQKNTLVDPVTGL VWDNISYQNAEPVINKELVLSYNQGTYIGAATQLFNHTGNKEYLEEAWKTTKSLITSPKLTYEGILRSEGQGDGGLFKG ILVRNLTLLAENPSLENQRKESLLNFLTYNANTLITFGLDKEEMVVGPNWAQKPKEHTDLSTQLSGVMMMEMAARLD LKQKKIKRYCQKNDKIHEKPHFINLSKEEVVIAVLGSSTAEGIGPSKKENAWVPRFMDYLQKSDTNFKIINLAKGGFTT ADLLPGGHPDKNISKALSYNPDAIIINLPSNDAAQGRSATDQIENYRKIFSSIKNEIPVWVTTPQPRNFEKAKVNIQKDMV DATYAFFDEEFIIDLWTYFSTEESLIKKEYDSGDGIHLNDEAHQLIFKRVVFSLSNFLKQA

6	>WP_079712844.1 hypothetical protein [*Salegentibacter salinarum*]	MRILNFGVFFFSSLLFLISCSSDDDSLEEIQEEEEEEQQPVDPGEEEKEEVGDWGEVAENLQEQTYSIYLTSNETFRQDNE<br>GNENFNYWWNAHMLDVLIDGYERTGDESYLSKMKSLLEGIKIKNGNKYENVFIDDMEWLGIACLRAYKLTDDEEYK<br>EVADLLWEEVKKGWSEVHGGGIAWKTDTPNSKNACSNGPAAIFALYLYEIDQDQEDLEWAKKIYHWLKDTLVDPES<br>GLVWDNINYHDGEAVINSDWIFTYNVGTYIGAANLLHQATGDDMYLDDALKSASSVVGPGQLTTGGVLKNEGQGDG<br>GLFKGILVRYFTQLTLNTDVPDDKQEEFEEFIQFNAETLYHNGLTNAGLAGPNWNDEPSGRVDLSTQLSGVMLMEAQA<br>LLERQ
7	>SSF83034.1 hypothetical protein [*Salegentibacter agarivorans*]	MRHKNYVIMRILVFGIFFLSSLLFLISCSTDNDNLEDIQEEEEEEQPVDPGDEEEDEVDNWAEVAENLQEETYSVYLASN<br>GTFRQDNEGNENFNYWWNAHMLDVLIDGYQRTGDESYLPKMKSLLEGIKIKNGNKYENVFIDDMEWLGIACLRAYK<br>LTNDEQYKEVADLLWEEVKKGWSDVHGGGIAWKTDTPNSKNACSNGPAAIFALYLYEIDQDQEDLEWAKKIYQWLN<br>DTLVDPESGLVWDNIDYQDGEAVINRDWIFTYNVGTYIGAANLLHQATGEEMYLEDAIKSASSVVGPGQITTGGVLKN<br>EGQGDGGLFKGILVRYFTQLTLNPNTPDDKQEEFKEFIQFNAETLYHNGLTTAGLAGPNWNDEPSGRVDLSTQLSGVM<br>LMEAKALLDRQ
8	>WP_093304467.1 hypothetical protein [*Salegentibacter agarivorans*]	MKFTSSNLSLAGLLKPFAPIFILITLGAHSLVFGKEKPTSVPSKLVTLENNNKWSHLANSLQETTYKNYLGENGVFTQDN<br>KEQNKFHYWWNAHMVDILIDGYLRTKDESYLPKIKDLVRGIKNSNQDNFQIYFNDDMEWLGIACVRAYRATGDEEY<br>KQVAEYLWEEVKKGWTDVHGGGIMWRTDTPEEKNACSNGPGALLALNLYAINSNEDELEWAKKIFEWQKNTLVDPL<br>TGLVWDNISYQDEEAVINKDLVLSYNQGTYIGAATQLFNHTGNKEYLEEAWKTTNTLMRSPKLTFEGILRSEGQGDGG<br>LFKGILVRNLTLLAENPALANQRKELLLDFLAFNANTLRTFGLDKKEMVVSPNWAQKPKNQTDLSTQLSGVMMMEM<br>AARLNLKQENVKRYCQQNEAIYKKPHFTTLSKEEIVIVVLGSSTAEGIGPTKKENAWVPRFIDYLQESDTNFKVINLAK<br>GGFTTADLLPDGHPDKNISKALSYNPEAIIINLPSNDAAEGRNTPEQLENYQKIFNSIKNEIPVWVTTPQPRNFEKAKVNI<br>QKDMVDATYAYFDKEFIIDLWTCFATEKNLIKKDYDSGDGIHLNDEAHKLIFERVVLSLSNFLKQG
9	>BAA75632.1 alpha-1,6-mannanase [*Bacillus circulans* TN31]	MSLRSGQLFRFLAVPLAIALMLGSMPGIGTSKAYAYTASDGDTAMKAFNDTFWDPNAKMFWKDSKREKHQDFWVEA<br>ELWELVMDAYQHTSDPALKAELKTQIDDVYDGTVAKYGQDWTNNPFNDDIMWWAMGSARAYQITGNPRYLEAAR<br>DHFDFVYDTQWDEEFANGGIWWLNSDHNTKNACINFPAAQAALYLYDITKDEHYLNAATKIFRWGKTMLTDGNGKV<br>FDRIEIEHGAVPDATHYNQGTYIGSAVGLYKATGNAVYLDDAVKAAKFTKNHLVDSNGVLNYEGPNGDLKGGKTILM<br>RNLAHLQKTLDETGQYPEFSAEFDEWLAFNIEMAWSHRNSDHIVDGNWAGQLLSGTYESWSSAAAVQALNGIKPME<br>AELHYGVKNPFDKIEAERYNIGSGFVLEGAFEGSLQLGGIQHGSYAAYKNVDFGSDGAIGFIARASSGTGGGNIEIRLDS<br>KDGPKVGTLNVEGTGDWNQYIDAVTLLKDDQGAPSTITGVHDVYLVFTKTNDDYLFNLNWVKFTTTDPTETDAYAK<br>LKAGNYDSSEGLSKHAEFGYLDAIHHNAYASYEGIDFGSINRRHLRAPWQHRGR
10	>CAC95995.1 Lin0763 protein [*Listeria innocua* Clip11262]	MKWSEYANLAQQSLEKFYLADTKEQFLNNFYPTENPEEDNKVFNYWWLAHLVEVRLDAYLRTKKQADLEVAEKTY<br>LHNKNRNGGTLIHDFYDDMLWNALAAYRLYKATGKSIYLEDAQLVWQDLVDTGWNDIMGGGFAWRRPQMYYKNT<br>PVNAPFIILSCWLYNELNETKYLEWAMKTYEWQTKVLVREDGFVEDGINRLEDGTIDYEWKFTYNQGVYIGANLELY<br>RITKEAKYLDTANKTAAISLKELTEDGIFKDEGNGGDEGLFKGIFYRYFTDLIEETANKTYRDFVLNSCQILVENAKLDG<br>YLLMGMNWKEKPSGKIPYSAELSGMIALEMAAKLE
11	>AAO78055.1 alpha-1,6-mannanase [*Bacteroides thetaiotaomicron* VPI-5482] (BT2949)	MIRISNKIKTLLAMLSFVQVTSGCDATVQDIIIDTDPGVEIGNNDYYTWCKETLSVIDKDLKISGTHSYYENQDRSQVSFI<br>WGNIFLLYTYTEGISLSKSEWSDALMNCFLNFDNYWHPNYKGIAGYATLPTSAEKVPDRFYDENGWTAIGLCDAYLA<br>TQNNSYLEKAKGALAFSLSGEDNVLGGGIYFQETFVSLPVQKNTICSAVTMLSCMKLYEITQDRQYLDAAIRINDWTV<br>ENLLDKSDNLLWDAKMVADGSVNTQKWSYNAGFMIRSWLKMYQATKDEKYLSQAKATLASSEAKWYNSINGALN<br>DPGYFAFSIIDSWFDMYDTDKNTVWLTKAFHAINFIHNKLRDGNGRYPEHWGTPTTSNLEKYDLRFSTVAAYMYMRA<br>ANYKRILND

12	>AAO78887.1 Glycoside hydrolase family 73 [*Bacteroides thetaiotaomicron* VPI-5482] (BT3782)	MRNICFVACMLFCLASASGKTVKNHPFVSIADSILDNVLNLYQTEDGLLTETYPVNPDQKITYLAGGAQQNGTLKASFL WPYSGMMSGCVAMYQATGDKKYKTILEKRILPGLEQYWDGERLPACYQSYPVKYGQHGRYYDDNIWIALDYCDYY RLTKKADYLKKAIALYEYIYSGWSDELGGGIFWCEQQKEAKHTCSNAPSTVLGVKLYRLTKDKKYLNKAKETYAWT RKHLCDPDDFLYWDNINLKGKVSKDKYAYNSGQMIQAGVLLYEETGDKDYLRDAQKTAAGTDAFFRSKADKKDPSV KVHKDMSWFNVILFRGFKALEKIDHNPTYVRAMAENALHAWRNYRDANGLLGRDWSGHNEEPYKWLLDNACLIELF AEIEK
13	>AAO78897.1 alpha-1,6-mannanase [*Bacteroides thetaiotaomicron* VPI-5482] (BT3792)	MKAIFKLLILNFLTLFIFPSCSDDDKSKSELNDPISGNISPVGSFAVEATNNENELLVKWTNPSNRDVDMVELSYRDVEA SLSRATDFSPGHIIIQVERDVTQEYMLKVPYFATYEVSAVAISKAGKRSVPESRVVMPYHEKVDEPELKLPEMLDRAHS YMTSVIGYYFGKSSRSCWRSNYPYDGKGYWDGDALVWGQGGGLSAFVAMRDATKESEVENLYGAMDDMMFKGIQ YFCQLDRGILAYSCYPAAGNERFYDDNVWIGLDMVDWYTETKEMRYLTQAKVVWRYLIDHGWDETCGGGVHWRE LNEHTTSKHSCSTGPTAVMGCKMYLATQEQEYLDWAIKCYDYMLDVLQDKSDHLFYDNVRPNKDDPNLPGDLEKNK YSYNSGQPLQAACLLYKITGEQKYLDEAYAIAESCHKKWFMPYRSKELNLTFNILAPGHAWFNTIMCRGFFELYSIDN DRKYIDDIEKSMIHAWSSSCHQGNNLLNDDDLRGGTTKTGWEILHQGALVELYARLAVLERENR
14	>AAO77738.1 Glycoside hydrolase family 73 [*Bacteroides thetaiotaomicron* VPI-5482] (BT2631)	MRNICFVACMLFCLTSAVGKTPGNTRYLSIADSILSNVLNLYQTNDGLLTETYPVNPDQKITYLAGGTQQNGTLKASFL WPYSGMMSGCVALYKATGNKKYKKILEKRILPGMEQYWDNSRLPACYQSYPTKYGQHGRYYDDNIWIALDYCDYY QLTHKPASLEKAVALYQYIYSGWSDEIGGGIFWCEQQKEAKHTCSNAPSTVLGVKLYRLTKDAKYLEKAKETYAWTK KHLCDPTDHLYWDNINLKGKVSKEKYAYNSGQMIQAGVLLYEETGDEQYLRDAQQTAAGTDAFFRTKADKKDPTVK VHKDMAWFNVILFRGLKALYKIDKNPAYVNAMVENALHAWENYRDENGLLGRDWSGHNKEQYKWLLDNACLIEFF AEI
15	>AAO77730.1 alpha-1,6-mannanase [*Bacteroides thetaiotaomicron* VPI-5482] (BT2623)	MKKVIKKYFFLALAIIMYSCNEDEKYDILERYTPETITSDEIAPVLNLQAQYMDSNSEIVLVTWMNPEDDFLSKVEISCC SANDNLLGEPVLLDAVSTKVGSYQTSLSVEERGYVKIVAINEKGVRSEARTAEILSSQQDFVYRADCLMSSVIELFFGG RYNAWNENYPNATGPYWDGIAAVWGQGAAYSGFVTMYKVTKETNNEKLRAKYAEKEETFLNSIDIFLNNGSGRKSF AYGTYIGPNDERYYDDNVWIGIEMANLYELTGNEVYLQHANTVWNFILEGIDDVTGGGVYWKEGAVSKHTCSTAPA AVMALKLYQLSKNESYLEIAKSLYSYCKDVLQDPNDYLFYDNVRLSDPSDKNSELKVSKDKFTYNSGQPMLAAAMLY RITKEEQFLKDAQNIAQSIYKKWFKNYHSSILDRDIMILSDPNTWFNAVMFRGFVELYKIDKNDVYVKAVKNTMEHA WQSNCRNRLTNLMSDDYAGDKKEGKWNIKTQGAFVEIFSLIGELEQLGCFQE
16	>ADO68190.1 alpha-L-arabinofuranosidase b [*Stigmatella aurantiaca* DW3]	MRQAKVLLTTLLASVAMTRCGGANETADVATASTRTVSVRVNGVGTGPVHVAPASADAPPGKAALSWVPLRLNAY YSFGVTTPNYTNRVLRHYESLARTDVLGTSPVEKADSSFRVVPGLADSGCYSLQSQNYPEKYLRHASSRVRIDSRDNT RGFDEDATWCTRPGLSGQGVSLESYNFPGRYMRHANSEVWLAQRGGPLPSDTEYSFNDDATWKAISQAGSDFRAWG EETLSKIEQDFRKPGSNLYFEGADRQSTAFHWGAGVQLHALIAGGKTQQAEAYANEMHQAYWCNTKGRWAYNAVA YSCGDRYYDDNAWVAKALMELHQKTNNATYLNRAKEVLAFSMSGENSAGSNPNGGIRWHEGDTGGQCLCATAPTA VANLMVYRATGTQQYLNDGLRLYNWVKANRFGYGPGYRGYENAVMTQAAILLFRITGNYAYLEDARHLALAMEST YIDWQTHALKETGQWGGHDMTNAYVDLYETDGDINWLNIVAGYLQFLRDNGKDANGRYPEVWSDVGKPGNPFLLY QASAARAFARMGNTRGGTAKLRDPVAVFQDCNYSGIWGAGFLMGRYTLSDLLFHGITGKDISSVRVQPGYKVTFYEN DNFGGASLVKTADDGCLVGAGWNDRVSSMVVEAVSPTVVVYKDCNFTNPGFNLPVGSYNEDTLRTLGLSPDVLSSLQ AADGYEAVLYDGGQFDQASYTTGTTSCLVGAGWNDKAASIVIRKKASP
17	>EAA30434.1 endo-1,6-alpha-	MRTTSSPRGATWLTALFAAAACLLPAANAQGYAIDTTDNIRASAKTLAFDLMKFYNGNQSGQIPGILPGPPSDGKGDY YWWEGGALMGTMIDYWHLTGDTTYNDVITQGILHQVGDNRDFQPLNFTASLGNDDQGFWGMTAMLAAENKFPNPP

	mannosidase [*Neurospora crassa* OR74A]	ADQPQWLALAQAVWATQAAPDRHDDTCNGGLRWQIPPTNNGYDYKNTIANAIFFNMGARLARYTRNDTYATWATK<br>QFQWIYDVNYIDHDSWKVYDGGHVEHNCTDINKAQFSYSAAILVQGAAFMYNYTEGDAATQDMWKTRIEKLTEGLF<br>RDFFPKGIAFELACEGRQGACTPDMVSFKGYVHRWMAMVTQIAPFTRDTILPVLKTSAEAAAKQCTGGATGRVCGFY<br>WSGGVFVDPAVDKTTGAGEAMDVLAAVSSLLIDEADPPVTNTTGGTSKGDPNAGTGSRHATEPAKPITTADKAGAAM<br>CTILLIAGGIAIWIFMNLGD
18	>CAC28653.1 endo-1,6-alpha-mannanase [*Neurospora crassa* OR74A]	MRWNVAVCGLMGLLAQSATAITMDIDDTQSVKDAAATIAYGMLKYYTGNNTGDTPGNLPDPYYWWEAGAMFGAM<br>VDYWWVTGDTSYVEVTTQAIVHQAGDARDFNPANQSRTSSNDDVGFWTITAMMAAEDAFPDPPPDQPQWLALVQA<br>VFNQMASRWDDLNCGGGLRWAINDFQTGKDYKNSISNGIFFNLGARLARFTGNSSYGEWASRTWDWERSINLITDEY<br>DVKDGAHFDVTTHVCRNDSGPHVWSYNIGVFLQGAAFMYNVSTGAEQETWKTRVDGLLGAVEAKFLTNDTKIIKEW<br>YCESGFSDRGHPYQCNIDQQTFKGYLLRWLSSTSQVAPYTYERINPWIRATAAAAVATCTGPVGAAAPQVDSGGIQPG<br>FKGIDGTACGFKWTQTFDGWAGVGAQMNALSAVMYTLTHKGVGKAAKGPVTTAQGGTSKGDPGAGVTDPASRGGL<br>AALKPITMADRVGAGIVTAILAISIVGGSVFLTI
19	> WP_070750786.1 [*Bacteroides thetaiotaomicron* MD40_GH76]	MKRLNIIFISLLAMVCTVLSCSDDSVEDKDIQKIDPVGQLEVYKTSREKEILVKFIRTNYVKDIQIEIAYRNTESGENGEW<br>TTIVLNGDNYKYGGNYLLQVPAEGTYEVAITLIGANELRSESKSQLASTFEYVKTSMFDCAHSMMTCVIKYYYHKGPR<br>TCWQTYYPKEQGYWDGDAVVWGQGGGLSAFVALREASVDTEQEEYYRSLEDDMFKGIQHFWVTDHGRTAYSVYPD<br>SGNDRFYDDNVWIGLDMAKWYAISKDVRYLNQAKAVWDYLSQYGWDNTCGGGVHWKELNEPSKSKHTCSTAPTG<br>VLSCKLYQLTHEQKYLDKAIECFNWLQAYMQDPSDHLYYDNVSPDPEDPTQPGRMETNKYSYNSGQPLQLACLLYKI<br>TKNESYLTVAHQIAEACHKKWFTSYHSEVLQRDFNILAPGHAWFNTVMCRGFFELYSIDKNPSYLEDVRNTMLHAWF<br>GKAHHISGLINDEDLSGAVSMNKWEILRQASLVELYALLAIWESGKDQTVLL

Supplementary Table S4: Primers used in this study for mutagenesis experiments of ShGH76 and cloning of ShGH92. The mutagenic codons are highlighted in bold. The primers were designed with SnapGene® software (from GSL Biotech; available at snapgene.com).

**Name of primer**	**Primer sequence (5' → 3')**
ShGH76_D136N_F	GTTTTTATC**AAT**GATATGGAATGGCTGGGTATTGCGTGCCT
ShGH76_D136N_R	CATTCCATATC**ATT**GATAAAAACGTTCTCATACTTGTTACCGTTACGC
ShGH76_D137N_F	TTTATCGAC**AAC**ATGGAATGGCTGGGTATTGCGTGCCT
ShGH76_D137N_R	CATTCCAT**GTT**GTCGATAAAAACGTTCTCATACTTGTTACCGTTAC
ShGH76_D136N_D137N_F	GTTTTTATC**AACAAT**ATGGAATGGCTGGGTATTGCGTGCCTGCGT
ShGH76_D136N_D137N_R	CATTCCAT**ATTGTT**GATAAAAACGTTCTCATACTTGTTACCGTTACGC
ShGH76_D136A_F	TATC**GCG**GATATGGAATGGCTGGGTATTGCGTGCCT
ShGH76_D136A_R	CATTCCATATC**CGC**GATAAAAACGTTCTCATACTTGTTACCGT
ShGH76_D137A_F	TATCGAC**GCC**ATGGAATGGCTGGGTATTGCGTGCCTG
ShGH76_D137A_R	CATTCCAT**GGC**GTCGATAAAAACGTTCTCATACTTGTTACCGTTAC
ShGH76_D136A_D137A_F	TTTTATC**GCGGCC**ATGGAATGGCTGGGTATTGCGTGCCTGCGT
ShGH76_D136A_D137A_R	CATTCCAT**GGCCGC**GATAAAAACGTTCTCATACTTGTTACCGTTACGCACTTCAA
ShGH92.1_F_NdeI	CGAGCTCATATGTTCCTGTTGTTTTTTTCCTG
ShGH92.1_R_XhoI	CGTGCGTCCTCGAGCTACTTATTTTCTCTATAAATTTCTTTATTTGGCT
ShGH92.2_F_NheI	CTGACTGCTAGCGATTACCTTGTAGAAAAGGTTGAAAATC
ShGH92.2_R_XhoI	GCTACTCTCGAGTTATAATTCATTAGTCAAAGAATACGGGAAATC
ShGH92.3_F_NheI	CTGGATGCTAGCTCTCATCCTGAAAGGGATTTG
ShGH92.3_R_NotI	ATCGTCGCGGCCGCTTAATTTGTCTTGAATTGCGGAGAT
ShGH92.4_F_NheI	ATCGTCGCTAGCAAATTAACGGAAAGGGTAAATGTTTT
ShGH92.4_R_XhoI	CTAGCTCTCGAGTCATTGTTCTGTTCTTTCTGGCA
ShGH92.5_F_NdeI	GCTACTCATATGTCCTCTAAGAAAGAAAAAGAGAGCG
ShGH92.5_R_XhoI	CGTAGCCTCGAGCTATTTGGTAATGACCTCTACCTC
ShGH92.6_F_NdeI	ATCGTCCATATGGCACCTTCGGAAAAACCG
ShGH92.6_R_XhoI	CAGTCGCTCGAGTTAGACATCCTTAAATAGACTTTTATTAGGAGC

Supplementary Table S5. Data collection and refinement statistics.

	GH76 (WT)	GH76^{ala} mutant	GH76^{asn} mutant
**X-ray source**	EMBL P14	DESY P11	DESY P11
**Wavelength (Å)**	0.9763	1.0332	1.0332
**Space group**	I 1 2 1	P2 21 21	P 1 21 1
**Unit cell**<br>**a, b, c (Å)**<br>**α, β, γ (º)**	 187.26, 37.51, 187.46<br>90, 100.95, 90	 37.15 85.31 95.03<br>90 90 90	 37.31 82.77 49.70 90<br>94.44 90
**Resolution range, (Å)[a]**	92.02 - 2.0 (2.04 - 2.0)	63.48 - 1.90 (1.94 - 1.90)	82.77-1.47 (1.50-1.47)
**R-merge [a]**	0.100 (0.845)	5.44 (13.60)	0.083 (0.371)
**Completeness (%)[a]**	99.4 (99.8)	99.2 (97.8)	99.88 (99.72)
**Multiplicity [a]**	6.6 (6.9)	9.3 (7.0)	5.6 (5.1)
**Mean I/sigma(I) [a]**	11.3 (2.6)	7.3 (1.0)	10.1 (3.3)
**No. of reflections [a]**	574248 (30691)	226365 (10694)	287715 (13225)
**No. of unique reflections [a]**	87258 (4422)	24400 (1522)	50967 (5083)
**Mosaicity**	0.11	0.94	0.38
Refinement			
**R_{work}/R_{free}**	0.152/0.203	0.118/0.172	0.162/0.199
**Number of non-hydrogen atoms**	8627	3103	3166
**Macromolecules**	8070	2689	2690
**Water**	557	351	439
**Ligands**	-	63	37
**Protein residues**	1014	339	338
**B factors**			
**Overall**	39.05	14.80	19.94
**Protein**	35.85	13.01	17.85
**Water**	41.73	27.32	32.89
**Ligands**	-	21.07	17.76
**R.m.s deviations**			
**Bond lengths (Å)**	0.008	0.017	0.007
**Bond angles (º)**	1.21	1.79	1.16
**Ramachandran statistics (%)**			
**Favored**	99.01	99.11	99.11
**Allowed**	0.99	0.89	0.89
**PDB accession code**	6SHD	6SHM	6Y8F

[a]Statistics for the highest-resolution shell are shown in parentheses.

Supplementary Table S6: Protein sequence identity (%) amongst GH92s from *Salegentibacter* sp. Hel_1_6 and *B. thetaiotaomicron* VPI-5482^{T}

ShGH92	BT2629	BT3773	BT3784	BT3858
WP_051935951.1 (GH92.1) (FG27DRAFT_1660)	33%	41%	33%	27%
WP_037317824.1 (GH92.2) (FG27DRAFT_1661)	62%	34%	63%	28%
WP_037317855.1 (GH92.3) (FG27DRAFT_1671)	48%	31%	49%	31%
WP_081912656.1 (GH92.4) (FG27DRAFT_1673)	23%	24%	24%	25%
WP_051935789.1 (GH92.5) (FG27DRAFT_1679)	28%	32%	28%	26%
WP_037317878.1 (GH92.6) (FG27DRAFT_1686)	29%	30%	28%	25%

Supplementary Table S7: Sequence and structural-guided similarity between GH76 from *Salegentibacter* sp. Hel_1_6 and its homologs.

**Microorganism (Protein name)**	**Sequence identity (%)**	**Matched secondary structure elements (%$_{SSE}$)**	**3D-Structural superimposition (Å, RMSD) (C^{α} atoms)**
Bacillus circulans (Aman6)	31	72	1.72 (305)
Listeria innocua (Lin0763)	37	78	1.41 (310)
B. thetaiotaomicron (BT3782)	27	72	1.96 (301)
B. thetaiotaomicron (BT3792)	25	72	1.68 (301)
B. thetaiotaomicron (BT2949)	19	78	2.11 (301)
B. thetaiotaomicron (BtGH76-MD40)	22	67	1.71 (304)

Supplementary Table S8: Prediction of localization of GH76s from *Salegentibacter sp*. Hel_1_6 and its homologs.

**Microorganism (Protein name)**	**SignalP 5.0 prediction**	**LipoP prediction**
Salegentibacter sp. Hel_1_6 (ShGH76)	Lipoprotein signal peptide (Sec/SPII)	SPII signal peptide
Bacillus circulans (Aman6)	Signal peptide (Sec/SPI)	SPI signal peptide
Listeria innocua (Lin0763)	No signal peptide	No signal peptide
B. thetaiotaomicron (BT3782)	Signal peptide (Sec/SPI)	SPI signal peptide
B. thetaiotaomicron (BT3792)	Lipoprotein signal peptide (Sec/SPII)	SPII signal peptide
B. thetaiotaomicron (BT2949)	Lipoprotein signal peptide (Sec/SPII)	SPII signal peptide
B. thetaiotaomicron (BtGH76-MD40)	Lipoprotein signal peptide (Sec/SPII)	SPII signal peptide

Supplementary figures legends

Supplementary Figure S1. Synthetic mannan-oligosaccharides (from Dr. Seeberger's group, Germany)

Supplementary Figure S2. Despite of speciation resulted in modification active site, *Salegentibacter sp.* Hel_1_6 PUL has high gene synteny with *S. salinarum* PUL. The substrate binding in ShGH76^{Ala} mutant (yellow) active site is compared with Phyre2 modelled structures of two GH76s from *S. salinarum* (SsGH76) (cyan and magenta). The modelled substrate has steric clash in active site pocket of SsGH76s.

Supplementary Figure S3. ShGH76^{Ala} mutant can be probable candidate for probing α-1,6-mannan backbone polysaccharide. (A) FACE gels of ShGH76^{WT} digested linear yeast α-mannan (left) and yeast α-mannan (right). The digested products were observed forming ladder like pattern at different time intervals, suggesting endo-acting mannanase activity for ShGH76^{WT} on linear yeast α-mannan substrate (left). On the other hand, there is very faint bands present in lane with 30 min and 16 h digestion of yeast α-mannan (right). **(B)** The Dot-Blot assay showed ShGH76^{Ala} mutant cannot interact with high affinity and thus did not show color development with yeast α-mannan in (first blot from left) nitrocellulose paper. Galactomannan (β-1,4-mannose backbone) control showed development of color with its specific antibody LM21 (fourth blot from left). Starch (α-1,4 and α-1,6-glucose backbone) is negative control. Concanavalin A has a high affinity for terminal mannose residue of polysaccharide hence presence of spot in the same blot indicates binding of polysaccharides to nitrocellulose membrane (third from left). **(C)** Dot-Blot

assay using linear yeast α-mannan with ShGH76^{Ala} and ShGH76^{Asn} mutants. ShGH76^{Ala} (first blot from left) has higher intensity color spot than ShGH76^{Asn} (second blot from left) with linear yeast α-mannan. For the epitope deletion experiment, ShGH76^{WT} was pre-incubated to digest the spotted linear yeast α-mannan and then the same blots were treated with ShGH76^{Ala} (third from left) and ShGH76^{Asn} (fourth from left). No color was observed when blots were spotted with the linear yeast α-mannan that was pre-incubated with ShGH76^{WT}. The controls are similar to the yeast α-mannan experiment in panel A.

Supplementary Figure S4. The primary sequence-based analysis of GH76 homologs revealed conservation of catalytic residues. Multiple sequence alignment of Full-length of GH76 (from *Salegentibacter* sp. Hel_1_6) with its homologs. A 3D structure-guided sequence alignment shows that the catalytic residues, two aspartate residues DD, which are conserved in the family GH76 (shown as blue color triangle). The active site-forming residues are shown with red color star. *Li*GH76: *Listeria innocua* GH76; *Bc*GH76: *Bacillus circulans* GH76; BT3782_GH76, BT3792_GH76 and BT2949_GH76: *Bacteroides thetaiotaomicron* GH76s.

Supplementary Figure S5. The highly charged and hydrophobic active sites have kinked oligomannans, not observed in previous homologs. Close view of active site of **(A)** ShGH76^{Ala} with Man4 substrate (yellow; stick representation) and **(B)** ShGH76^{Asn} with Man3 substrate (yellow; stick representation). **(C)** Comparison of mannopentaose (Man5: yellow stick representation) binding in B*c*GH76 (pink) with ShGH76^{WT} (grey). Linear Man5 has a strong steric clash with W254 of GH76^{WT} (red color), indicating a different mode or conformation of the substrate in the active site. **(D)** Active-site of ShGH76^{Ala} mutant displaying mannotetrose (Man4)

(ball-and-stick representation) with its electron density (green). Electron density maps are REFMAC maximum-likelihood/σ_A weighted $2F_{obs}$-F_{calc} syntheses contoured at 1σ for Man4. **(E)** Active-site of the ShGH76^{Asn} mutant with mannotriose (Man3) (ball-and-stick representation) electron density (green). Electron density maps are REFMAC maximum-likelihood/σ_A weighted $2F_{obs}$-F_{calc} syntheses contoured at 1σ for Man3.

Supplementary Figure S6. GH76 has no global structural changes upon binding of substrate into its highly negative charged and conserved active site pocket. (A) Putty representation of GH76 WT and two mutants to highlight global structural similarity. The size of the tube is proportional to the mean r.m.s. deviation per residue between Cα pairs, indicating areas of weak and strong structural conservation. **(B)** Close-up of active site of ShGH76s superimpositions to show all Aspartate involved in substrate binding, and **(C)** ShGH76^{WT} ConSurf surface representation highlighting modelled Man4 from mutant structure.

Supplementary Figure S7. Despite conserved fold of GH76 family, all α-1,6-linked oligomannans adapted different binding in active site. (A) Structural differences (highlighted circles) between ShGH76 and human gut bacterial GH76s (BT2949-GH76: PDB 4V1S, BT3792-GH76: PDB 4C1S, BT3782-GH76: PDB 4MU9) represented in cartoon format with a rainbow color scheme (NT = blue; CT = red). The loops that connect helices or strands have different conformations. **(B)** Superimposition of ShGH76 represented in cartoon format with a rainbow color scheme (NT = blue; CT = red) on other bacterial GH76s (*Listeria innocua Li*GH76: PDB 3K7X, *Bacillus circulans Bc*GH76: 5AGD, Bovine *B. thetaiotaomicron Bt*GH76-MD40: PDB 6U4Z) showing not only loops conformational changes, but also extended loops in the 6U4Z

structure. ShGH76 has additional NTD in its 6U4Z structure, which has not been observed in any other GH76 so far. **(C)** α-1,6-linked oligomannans Man2 (PDB 4BOJ from *B. circulans*), Man3 (ShGH76^{Asn} mutant), Man4 (ShGH76^{Ala} mutant) and Man5 (PDB 5AGD from *B. circulans*) with active site residues are superimposed. It suggested conformational variation in terminal mannose monomers at non-reducing at due to presence of two different amino acids. **(D)** Ligands were superimposed to show Man3 and Man4 has kinked arrangement in ShGH76 mutants.

Supplementary Figure S8. Poorly active on yeast α-mannan ShGH76 has efficient activity on linear α-1,6-mannan revealed by kinetic parameters and three *Sh*GH92s present in same PUL exhibited endo-/exo-mannosidase activities. (A) HPAEC-PAD chromatographs showing ShGH76^{WT} activity on yeast α-mannan (YM) after 30 min and after 16 h incubations. The heat-killed enzyme is control. **(B)** Determination of Kinetic parameters on Linear yeast α-mannan (LYM) of the ShGH76^{WT}. LYM is yeast mannan, isolated from a *S. cerevisiae* mutant, that comprises an undecorated α-1,6-Man-linked polysaccharide. Kinetic parameters were determined from technical duplicates non-linear (k_{cat} and K_M shown) or linear (k_{cat}/K_M only) regression plots due to limited substrate availability. **(C)** FACE gel of *Sh*GH92s digested yeast α-mannan (YM) showing exo-α-mannosidase activity of GH92.1 and GH92.3 while GH92.2 acts as α-mannanase from the pattern of the bands of products upon digestion. Standard lane comprises of manno-oligosaccharide mixture containing Man1, Man2, Man3, Man4, Man5 and Man7.

Supplementary Figure S9. The minimum substrate for ShGH76 endo-α-mannanase is mannotetrose. HPAEC-PAD chromatographs showing ShGH76^{WT} activity on **(A)** mannobiose

(Man2) **(B)** Mannotriose (Man3) **(C)** Mannotetrose (Man4) **(D)** Mannopentose (Man5) **(E)** Mannohexose (Man6) after 30 min and after 16 h incubations. The heat-killed enzyme is control.

References

1. Crawford C, Oscarson S. Optimized conditions for the palladium-catalyzed hydrogenolysis of benzyl and naphthylmethyl ethers: preventing saturation of aromatic protecting groups. *Eur J Org Chem*. 2020;2020(22):3332-7.
2. Crawford CJ, Qiao Y, Liu Y, Huang D, Yan W, Seeberger PH, et al. Defining the qualities of high-quality palladium on carbon catalysts for hydrogenolysis. *Org Process Res Dev*. 2021;25(7):1573-8.
3. Lever M. A new reaction for colorimetric determination of carbohydrates. *Anal Biochem*. 1972;47(1):273-9.
4. Fontaniella B, Millanes AM, Vicente C, Legaz ME. Concanavalin A binds to a mannose-containing ligand in the cell wall of some lichen phycobionts. *Plant Physiol Biochem*. 2004;42(10):773-9.
5. Davies G, Henrissat B. Structures and mechanisms of glycosyl hydrolases. *Structure*. 1995;3(9):853-9.
6. Cuskin F, Lowe EC, Temple MJ, Zhu Y, Cameron E, Pudlo NA, et al. Human gut *Bacteroidetes* can utilize yeast mannan through a selfish mechanism. *Nature*. 2015;517(7533):165-9.
7. Thompson AJ, Cuskin F, Spears RJ, Dabin J, Turkenburg JP, Gilbert HJ, et al. Structure of the GH76 α-mannanase homolog, BT2949, from the gut symbiont *Bacteroides thetaiotaomicron*. *Acta Crystallogr D: Biological Crystallography*. 2015;71(2):408-15.
8. Jones DR, Xing X, Tingley JP, Klassen L, King ML, Alexander TW, et al. Analysis of active site architecture and reaction product linkage chemistry reveals a conserved cleavage

substrate for an endo-alpha-mannanase within diverse yeast mannans. *J Mol Biol*. 2020;432(4):1083-97.

9. Belz T, Jin Y, Coines J, Rovira C, Davies GJ, Williams SJ. An atypical interaction explains the high-affinity of a non-hydrolyzable S-linked 1,6-alpha-mannanase inhibitor. *ChemComm*. 2017;53(66):9238-41.

10. Juncker AS, Willenbrock H, Von Heijne G, Brunak S, Nielsen H, Krogh A. Prediction of lipoprotein signal peptides in Gram-negative bacteria. *Protein Sci*. 2003;12(8):1652-62.

11. Almagro Armenteros JJ, Tsirigos KD, Sonderby CK, Petersen TN, Winther O, Brunak S, et al. SignalP 5.0 improves signal peptide predictions using deep neural networks. *Nat Biotechnol*. 2019;37(4):420-3.

12. Nerinckx W, Desmet T, Claeyssens M. A hydrophobic platform as a mechanistically relevant transition state stabilising factor appears to be present in the active centre of all glycoside hydrolases. *FEBS lett*. 2003;538(1-3):1-7.

13. Vocadlo DJ, Davies GJ. Mechanistic insights into glycosidase chemistry. *Curr Opin Chem Biol*. 2008;12(5):539-55.

14. Thompson AJ, Speciale G, Iglesias-Fernandez J, Hakki Z, Belz T, Cartmell A, et al. Evidence for a boat conformation at the transition state of GH76 alpha-1,6-mannanases--key enzymes in bacterial and fungal mannoprotein metabolism. *Angewandte Chemie*. 2015;54(18):5378-82.

15. Nedashkovskaya OI, Kim SB, Vancanneyt M, Shin DS, Lysenko AM, Shevchenko LS, et al. *Salegentibacter agarivorans* sp. nov., a novel marine bacterium of the family Flavobacteriaceae isolated from the sponge *Artemisina sp*. *Int J Syst Evol Microbiol*. 2006;56(Pt 4):883-7.

16. Xia HF, Li XL, Liu QQ, Miao TT, Du ZJ, Chen GJ. *Salegentibacter echinorum* sp. nov., isolated from the sea urchin *Hemicentrotus pulcherrimus*. *Antonie Van Leeuwenhoek*. 2013;104(3):315-20.

17. Ivanova EP, Bowman JP, Christen R, Zhukova NV, Lysenko AM, Gorshkova NM, et al. *Salegentibacter flavus* sp. nov. *Int J Syst Evol Microbiol*. 2006;56(Pt 3):583-6.

18. Nedashkovskaya OI, Suzuki M, Vancanneyt M, Cleenwerck I, Zhukova NV, Vysotskii MV, et al. *Salegentibacter holothuriorum* sp. nov., isolated from the edible holothurian *Apostichopus japonicus*. *Int J Syst Evol Microbiol*. 2004;54(Pt 4):1107-10.

19. Nedashkovskaya OI, Kim SB, Lysenko AM, Mikhailov VV, Bae KS, Kim IS. *Salegentibacter mishustinae* sp. nov., isolated from the sea urchin *Strongylocentrotus intermedius*. *Int J Syst Evol Microbiol*. 2005;55(Pt 1):235-8.

20. Yoon JH, Jung SY, Kang SJ, Jung YT, Oh TK. *Salegentibacter salarius* sp. nov., isolated from a marine solar saltern. *Int J Syst Evol Microbiol*. 2007;57(Pt 12):2738-42.

21. Dobson SJ, Colwell RR, McMeekin TA, Franzmann PD. Direct sequencing of the polymerase chain reaction-amplified 16S rRNA gene of Flavobacterium *gondwanense* sp. nov. and Flavobacterium *salegens* sp. nov., two new species from a hypersaline Antarctic lake. *Int J Syst Bacteriol.* 1993;43(1):77-83.

22. Yoon JH, Lee MH, Kang SJ, Oh TK. *Salegentibacter salinarum* sp. nov., isolated from a marine solar saltern. *Int J Syst Evol Microbiol.* 2008;58(Pt 2):365-9.

23. Liang QY, Xu ZX, Zhang J, Chen GJ, Du ZJ. *Salegentibacter sediminis* sp. nov., a marine bacterium of the family Flavobacteriaceae isolated from coastal sediment. *Int J Syst Evol Microbiol.* 2018;68(7):2375-80.
