# Supplementary figures and images for: Glycoside hydrolase from the GH76 family indicates that marine Salegentibacter sp. Hel_I_6 consumes alpha-mannan from fungi
Source: ISME J. 2022 Apr 12;16(7):1818–30. doi: 10.1038/s41396-022-01223-w (PMC9213526; doi:10.1038/s41396-022-01223-w)

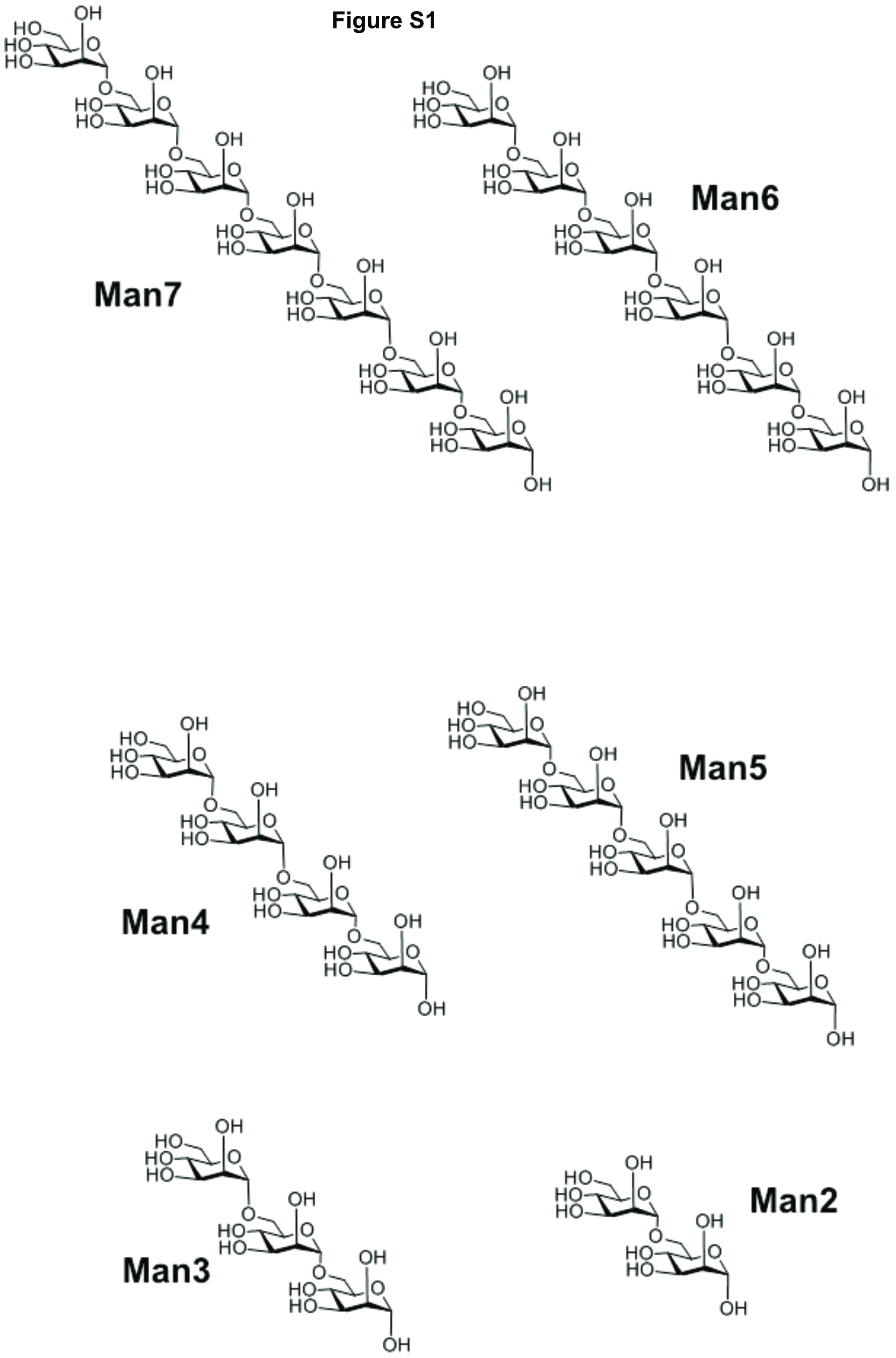

Supplement: Supplementary file 2 — Figure S1 [file 41396_2022_1223_MOESM2_ESM.tif]

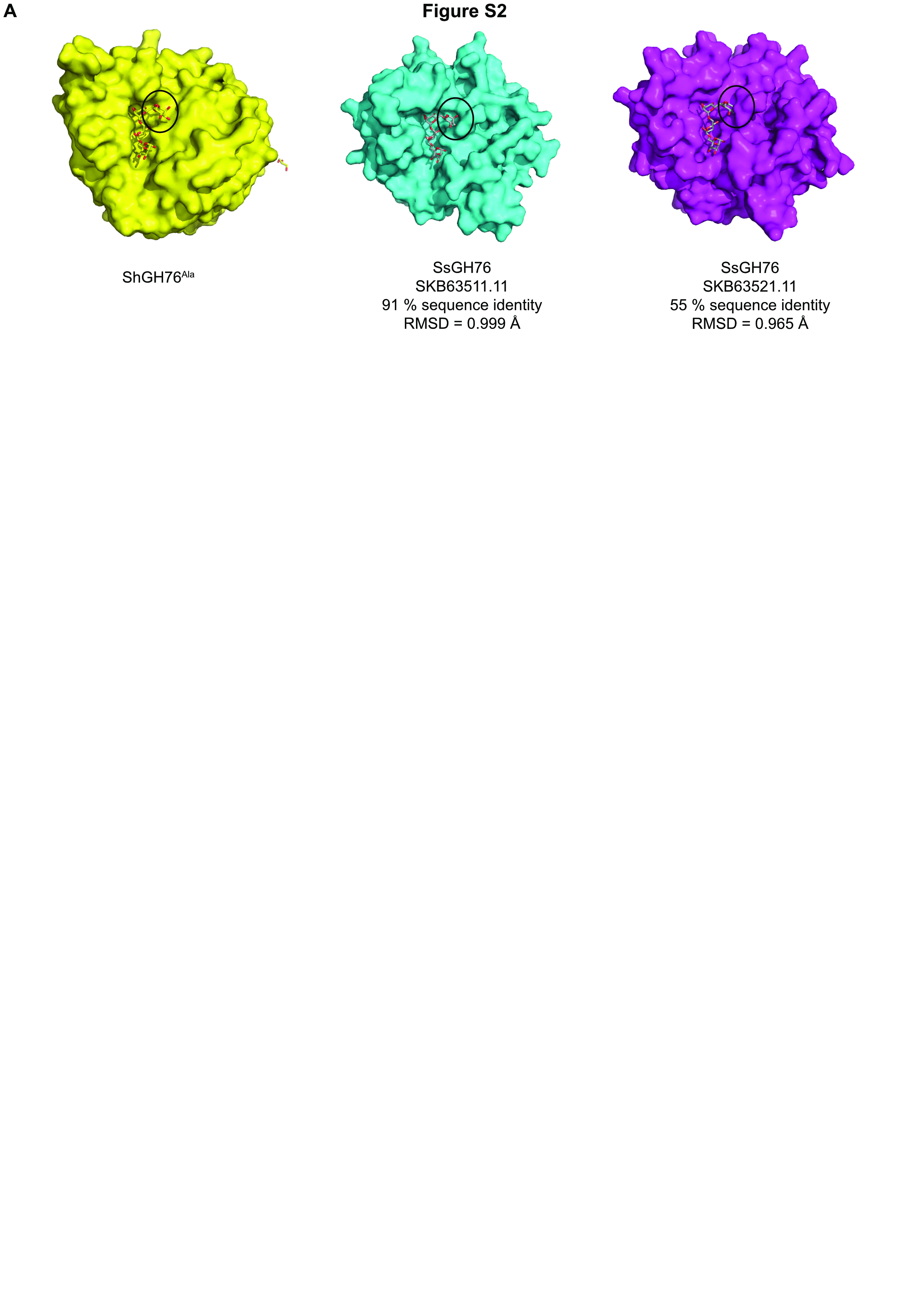

Supplement: Supplementary file 3 — Figure S2 [file 41396_2022_1223_MOESM3_ESM.tif]

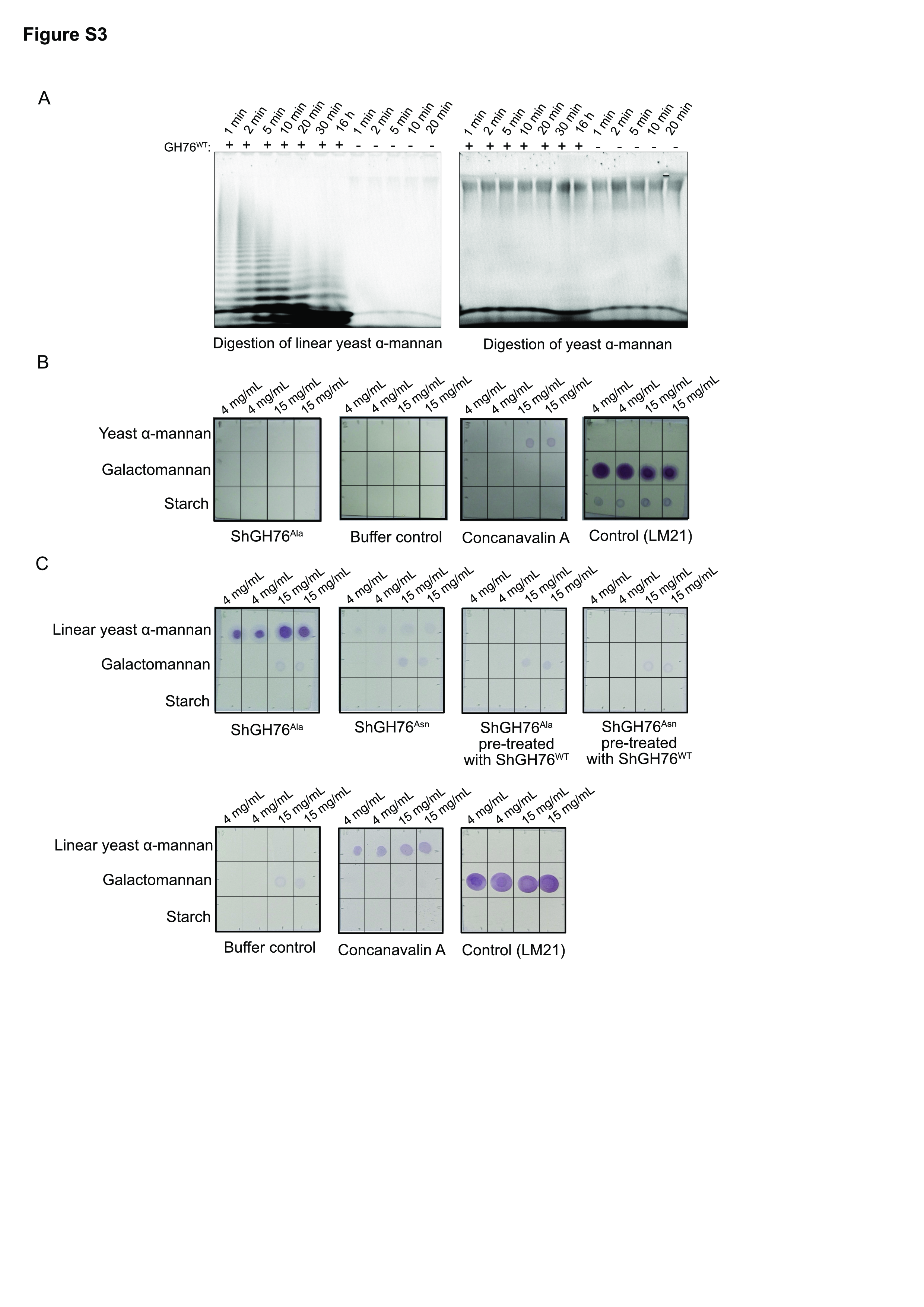

Supplement: Supplementary file 4 — Figure S3 [file 41396_2022_1223_MOESM4_ESM.tif]

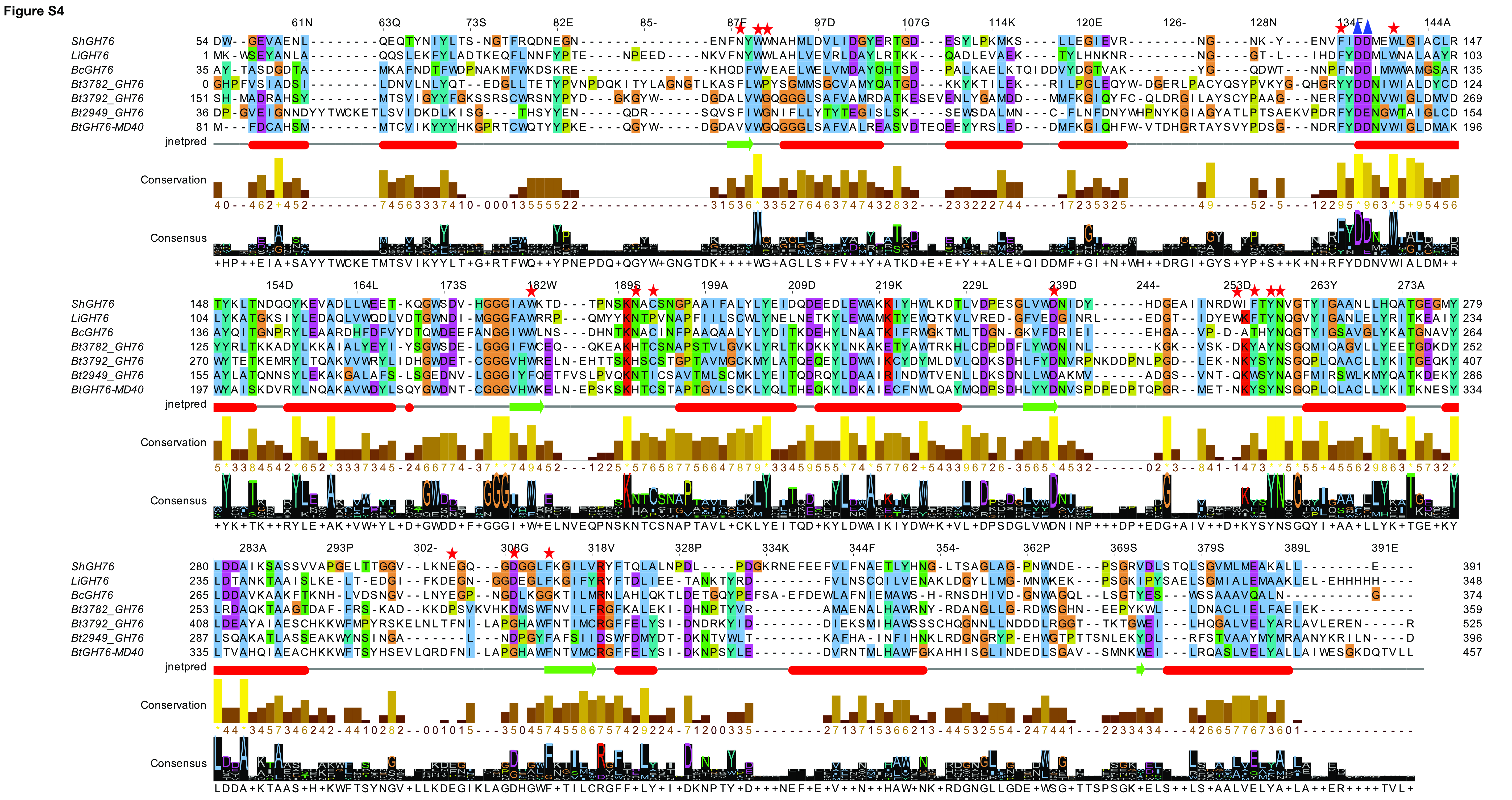

Supplement: Supplementary file 5 — Figure S4 [file 41396_2022_1223_MOESM5_ESM.tif]

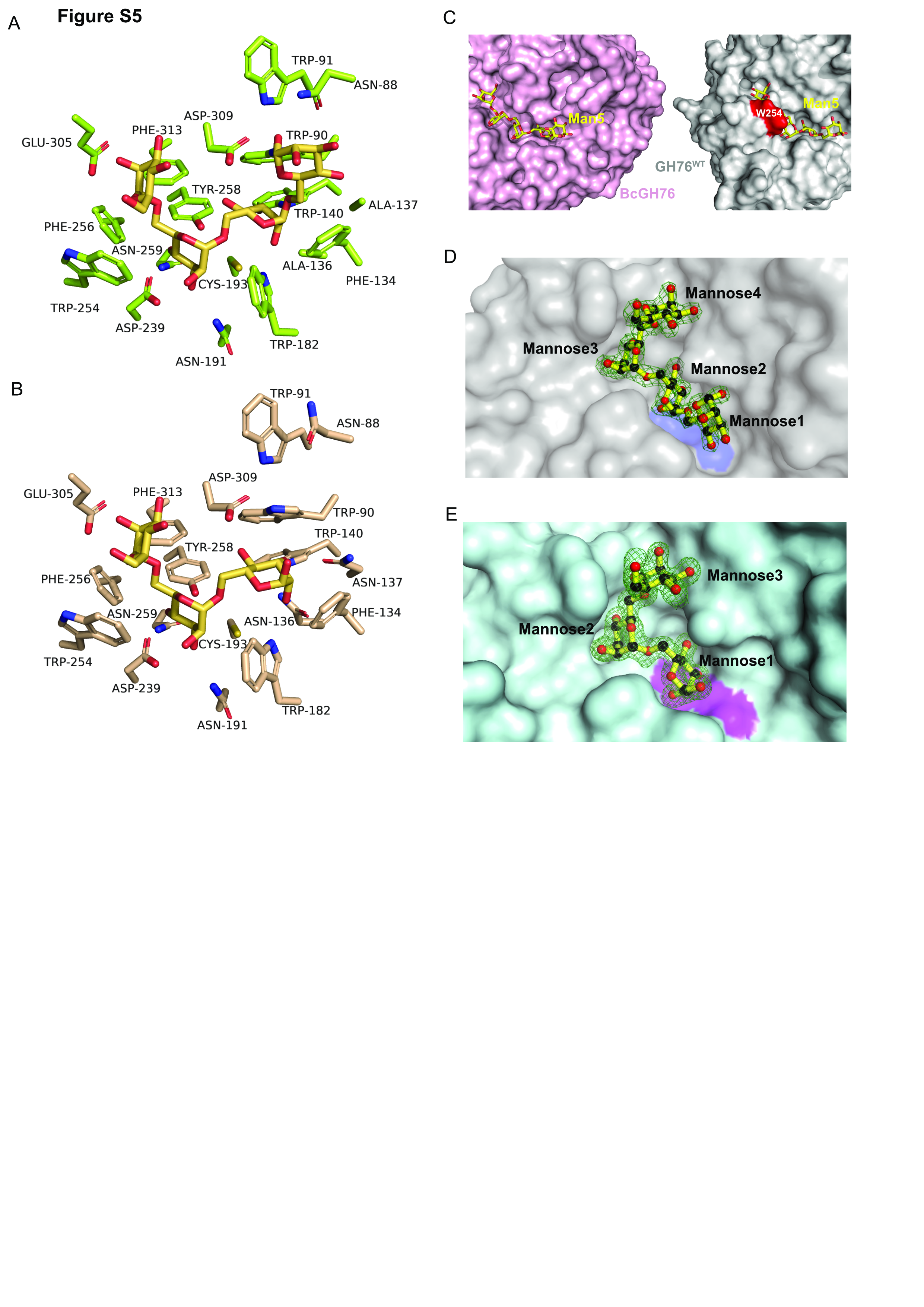

Supplement: Supplementary file 6 — Figure S5 [file 41396_2022_1223_MOESM6_ESM.tif]

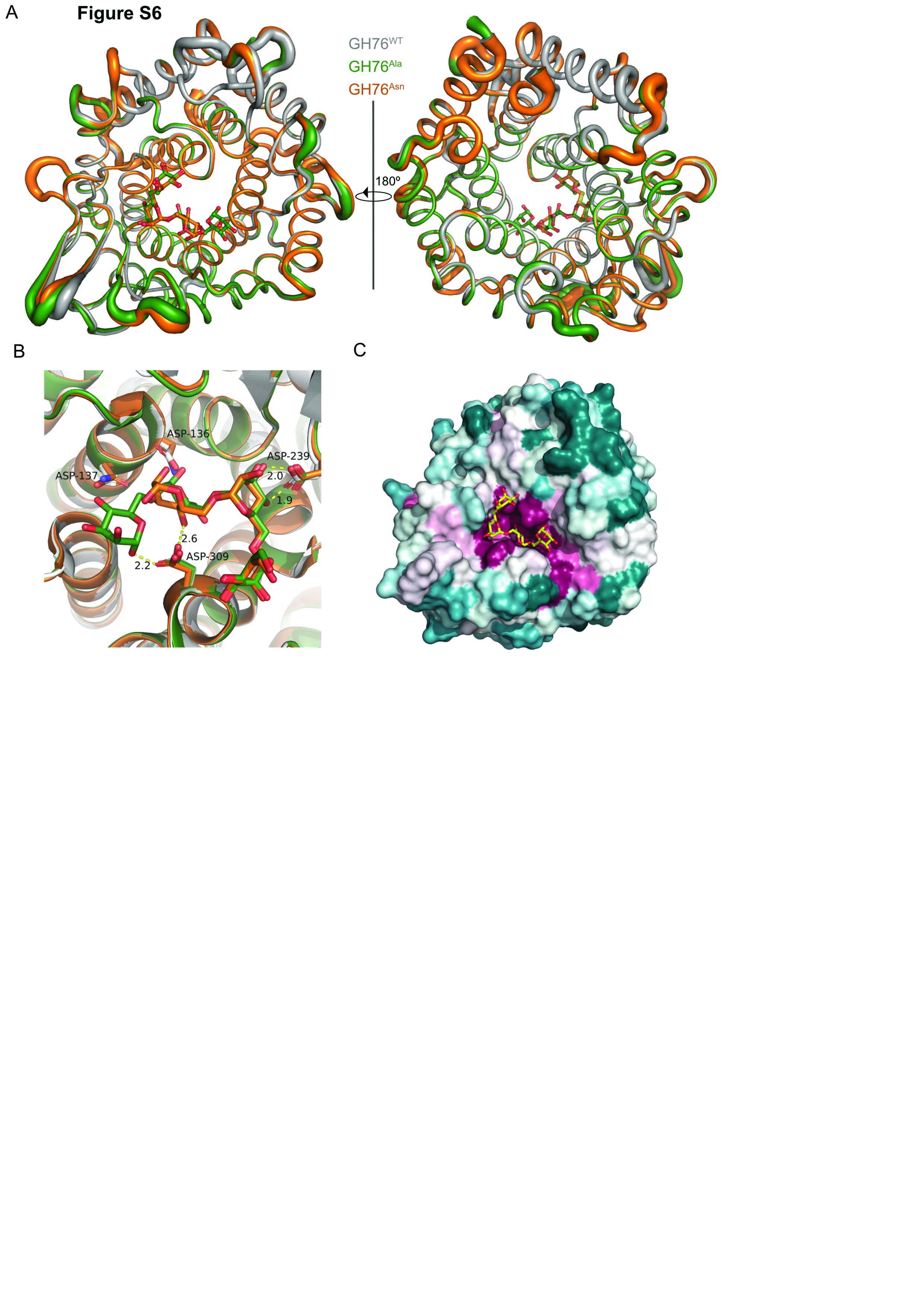

Supplement: Supplementary file 7 — Figure S6 [file 41396_2022_1223_MOESM7_ESM.tif]

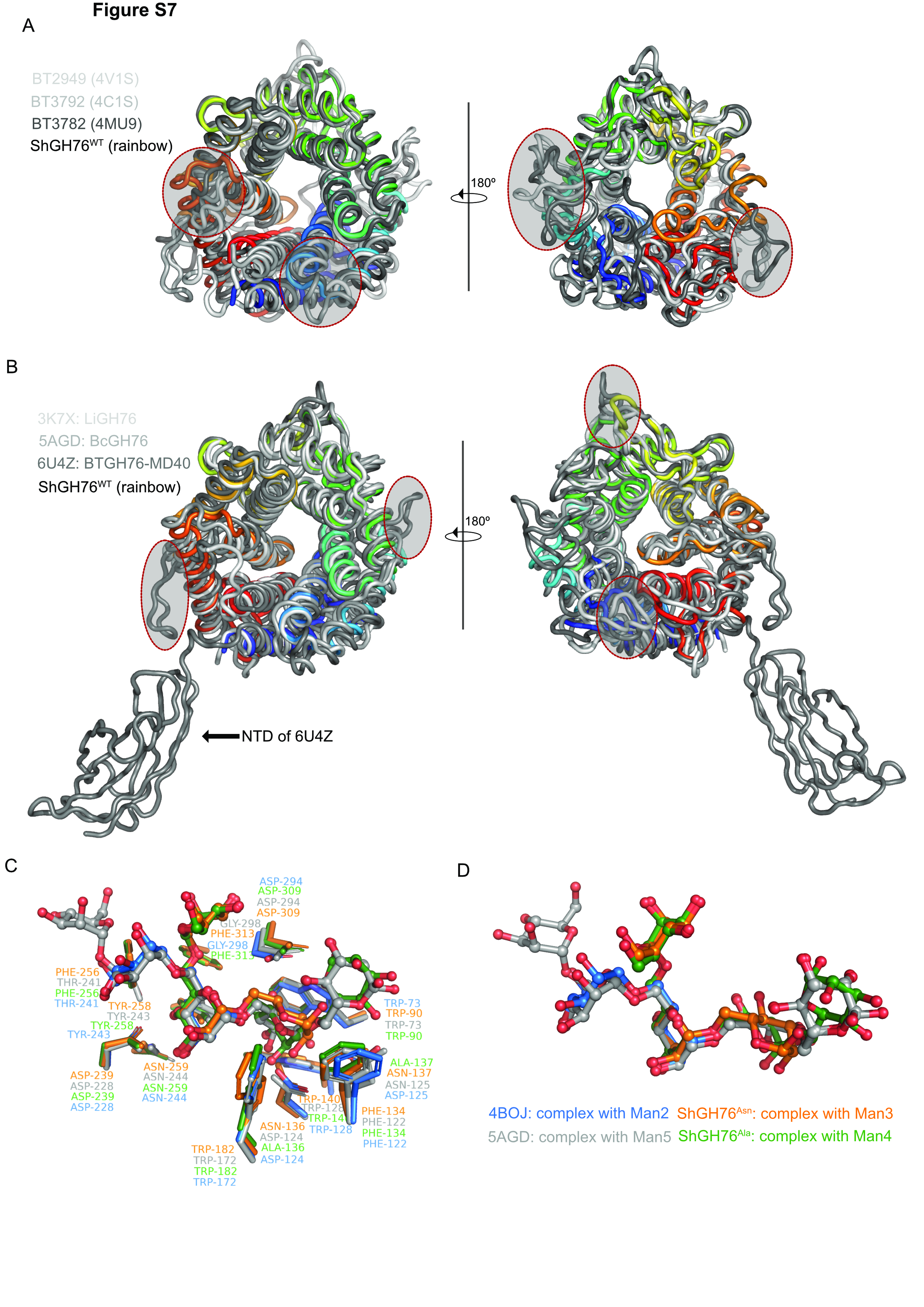

Supplement: Supplementary file 8 — Figure S7 [file 41396_2022_1223_MOESM8_ESM.tif]

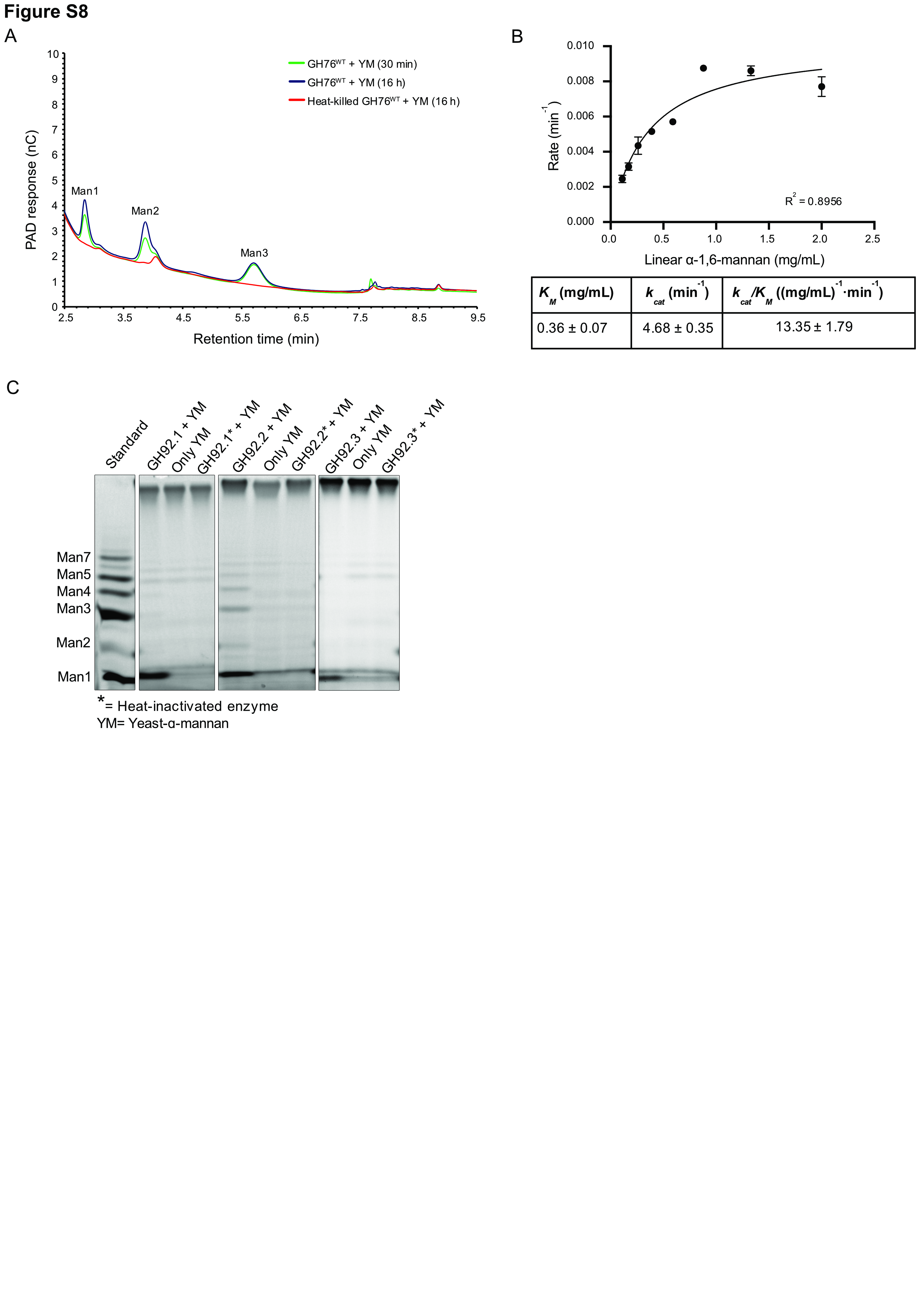

Supplement: Supplementary file 9 — Figure S8 [file 41396_2022_1223_MOESM9_ESM.tif]

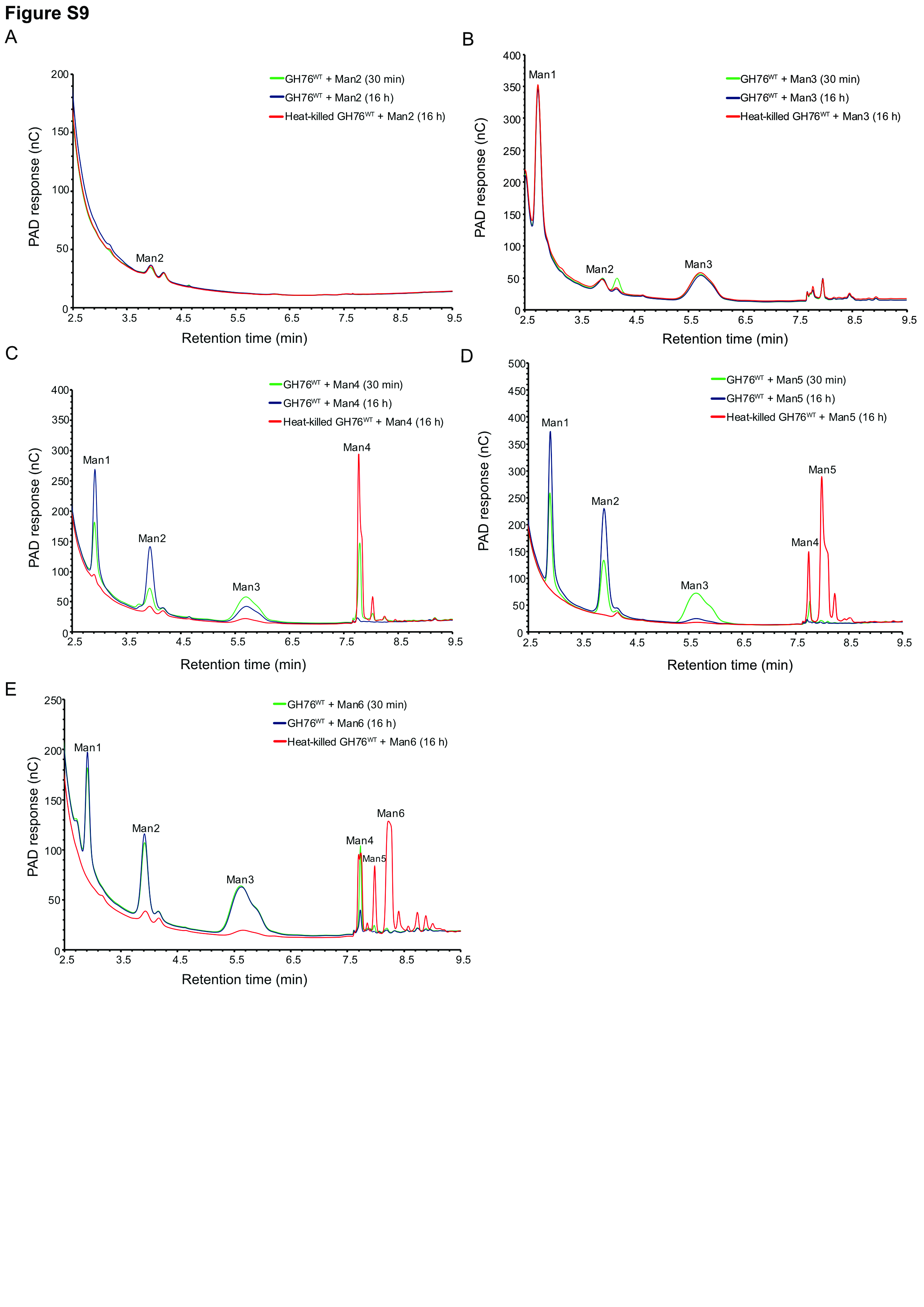

Supplement: Supplementary file 10 — Figure S9 [file 41396_2022_1223_MOESM10_ESM.tif]
